# Genetic Structure of a Worldwide Germplasm Collection of *Prunus armeniaca* L. Reveals Three Major Diffusion Routes for Varieties Coming From the Species’ Center of Origin

**DOI:** 10.3389/fpls.2020.00638

**Published:** 2020-05-25

**Authors:** Hedia Bourguiba, Ivan Scotti, Christopher Sauvage, Tetyana Zhebentyayeva, Craig Ledbetter, Boris Krška, Arnaud Remay, Claudio D’Onofrio, Hiroyuki Iketani, Danilo Christen, Lamia Krichen, Neila Trifi-Farah, Weisheng Liu, Guillaume Roch, Jean-Marc Audergon

**Affiliations:** ^1^LR99ES12, Laboratoire de Génétique Moléculaire, Immunologie et Biotechnologie, Faculté des Sciences de Tunis, Université Tunis El Manar, Tunis, Tunisia; ^2^INRA Centre PACA, UR 629 URFM, Avignon, France; ^3^INRA Centre PACA, UR 1052 GAFL, Montfavet, France; ^4^Schatz Center for Tree Molecular Genetics, The Pennsylvania State University, University Park, PA, United States; ^5^San Joaquin Valley Agricultural Sciences Center, Crop Diseases, Pests & Genetics, Parlier, CA, United States; ^6^Department of Fruit Growing, Faculty of Horticulture, Mendel University, Lednice, Czechia; ^7^Pôle de Génotypage BioGeves, Surgères, France; ^8^Dipartimento di Scienze Agrarie, Alimentari e Agro-Ambientali, Università di Pisa, Pisa, Italy; ^9^National Agriculture and Food Research Organization (NARO) Institute of Fruit Tree Science, Tsukuba, Japan; ^10^Département Fédéral de L’économie DFE, Station de Recherche Agroscope Changins-Wädenswil ACW, Centre de Recherche Conthey, Conthey, Switzerland; ^11^Liaoning Institute of Pomology, Yingkou City, China; ^12^CEP Innovation, Lyon, France

**Keywords:** apricot, *Prunus armeniaca* L., diversity pattern, population structure, SSR markers, core collection, history of diffusion, Approximate Bayesian Computation

## Abstract

The characterization of the largest worldwide representative data set of apricot (*Prunus armeniaca* L.) germplasm was performed using molecular markers. Genetic diversity and structure of the cultivated apricot genetic resources were analyzed to decipher the history of diffusion of this species around the world. A common set of 25 microsatellite markers was used for genotyping a total of 890 apricot accessions in different collections from the center of origin to the more recent regions of apricot culture. Using a Bayesian model-based clustering approach, the apricot genotypes can be structured into five different genetic clusters (F_ST_ = 0.174), correlated with the geographical regions of origin of the accessions. Accessions from China and Central Asia were clustered together and exhibited the highest levels of diversity, confirming an origin in this region. A loss of genetic diversity was observed from the center of origin to both western and eastern zones of recent apricot culture. Altogether, our results revealed that apricot spread from China and Central Asia, defined as the center of origin, following three major diffusion routes with a decreasing gradient of genetic variation in each geographical group. The identification of specific alleles outside the center of origin confirmed the existence of different secondary apricot diversification centers. The present work provides more understanding of the worldwide history of apricot species diffusion as well as the field of conservation of the available genetic resources. Data have been used to define an apricot core collection based on molecular marker diversity which will be useful for further identification of genomic regions associated with commercially important horticultural traits through genome-wide association studies to sustain apricot breeding programs.

## Introduction

Evaluation of the extent and structure of genetic variation in germplasm collections has important implications for plant breeding programs and the conservation of genetic resources.

In fact, the domestication process of crop species involves the production of populations with modified traits selected according to human pressure compared to ancestral material (Zohary et al., 2012).

Apricot (*Prunus armeniaca* L.) which belongs to the *Rosaceae* family, is cultivated worldwide. It is an important fruit in the Northern hemisphere, representing the third most planted stone fruit species after peach and plum. Among all temperate fruits, apricot represents the seventh in terms of worldwide production. Apricots are native to China and Central Asia, arising following two successive domestication events, one in Western Central Asia (Fergana valley, at the borders of Uzbekistan, Tajikistan, and Kyrgyzstan) and one in China (Vavilov, 1951; Faust et al., 1998). In these regions, apricot production is focused on the development of cultivars for fresh market, kernel production and ornamental use. Apricots are mainly self-incompatible, with fruits without specific aroma (Zhebentyayeva et al., 2012).

Based on morphological and physiological traits, Kostina (1964) recognized four main eco-geographical groups: Central Asian, Irano-Caucasian, European, and Dzhungar-Zailij. Apricots from Central Asia and the Xinjing Province of China are genetically related to wild forms of *P. armeniaca* and are distinguished from the East Asian apricots which are related to East Asian wild species.

From the center of origin, apricot culture spread to the Irano-Caucasian region which constitutes the secondary center of apricot diversification following the Silk Road (Vavilov, 1951). Generally, the apricots from this region have lower chilling requirements than in Central Asia, most of them are self-incompatible and the fruits are mainly consumed dried. From the Irano-Caucasian area, apricot was brought to the Mediterranean region around the first century BC (Zohary et al., 2012) following two diffusion routes: the first one following Southern Europe and the second one through Northern Africa (Bourguiba et al., 2012b). The Mediterranean countries have the highest level of apricot production in the world. In the Mediterranean basin, apricots were exclusively cultivated for fresh fruits and exhibited rather low chilling requirements. Bourguiba et al. (2012b) revealed three major apricot gene pools throughout the Mediterranean Basin: the “Irano-Caucasian,” the “North-Mediterranean Basin” and the “South-Mediterranean Basin.” This latter is composed of apricots from the Maghreb region (Tunisia, Algeria and Morocco) in North-Africa, encompassing both grafted and seed-propagated apricot accessions presenting the same level of genetic diversity probably due to the existence of extensive gene flow among them during the Andalusian period (Bourguiba et al., 2012a; Mamouni et al., 2014). Seed-propagated apricots in North-Africa are specific to traditional oasis agroecosystems, and well-adapted to arid and Saharan climates. Focusing on European apricot germplasm, Faust et al. (1998) suggested that apricot accessions from Eastern Europe were clearly distinguished from other accessions of European origin, suggesting that this region contained specific genetic variability. In fact, apricots are self-compatible and specifically adapted to winter frost through high chilling requirements. More recently, apricot was brought to growing regions in the world like North America, South Africa, Australia and New Zealand. Improvement strategies have been engaged all over the world with hybridization programs to overcome the lack of regionally-adapted cultivars. As an example, the breeding history of North American apricot germplasm, which was characterized by highly desirable fruit appearance with poor flavor and a natural resistance to disease, started from a limited number of European cultivars which were later enriched through introgression of genetic material originating from elsewhere (Faust et al., 1998).

A gradient of decreasing genetic diversity was revealed from the Eastern to the South-Western Mediterranean Basin using different molecular markers (Hagen et al., 2002; Bourguiba et al., 2012b). In fact, it is widely argued that the genetic diversity of crops has suffered an overall reduction with time and selection, due to the bottlenecks experienced during the domestication process. Domestication syndrome traits in vegetative crops represent tendencies in human-mediated plant evolution that reflect a combination of permanent genetic changes and impermanent plastic responses to cultivation practices. Thus, vegetative propagation enables more controlled selection of favored phenotypic characteristics than under sexual reproduction (Denham et al., 2020). Conversely, no bottlenecks were identified from the wild genetic pool in Central Asia to European, Southern Central Asia and Chinese cultivated apricots (Decroocq et al., 2016; Liu et al., 2019). This is probably explained by the limited number of generations since domestication due to long juvenile phases, ongoing gene flow between domesticated and wild accessions (Miller and Gross, 2011; Gaut et al., 2015) and, to a lesser extent, to clonal propagation (because it is difficult to carry out on apricot species in comparison with plum, peach, apple and grape). Recently, Denham et al. (2020) attested that spontaneous sexually reproduced progeny may also be incorporated into clonally reproduced crop assemblages thereby enabling gene flow and potentially prolonging the period of domestication.

Molecular markers are particularly useful for the evaluation of genetic diversity and structure in *Prunus* species. Until now, simple sequence repeat (SSR) markers that rely on genomic sequences have been proven to be the most widely used type of DNA marker in characterizing germplasm collections, because of their easy use, relatively low cost, high degree of polymorphism and informativeness provided by the large number of alleles per locus (Van Inghelandt et al., 2010).

In recent years, the estimation of the genetic diversity and population structure of apricot species have been assessed and understanding increased on the domestication of this fruit crop. However, to date, because of the lack of representativeness of worldwide germplasm collection in previous studies, the overall view of cultivated apricot demographic history remains to be characterized. In the present study, we investigated an apricot collection composed of 890 diverse accessions issued from seven different regions around the world, genotyped for 25 neutral microsatellite markers.

The objectives were to: *i*) evaluate the genetic diversity *ii*) describe worldwide apricot population structure, *iii*) elucidate the demographic history of diffusion for the apricot species from the center of origin to the recent regions of culture, and iv) estimate the domestication bottlenecks within cultivated apricot.

## Materials and Methods

### Plant Material and DNA Extraction

Apricot germplasm included 890 cultivated accessions from different geographic origins. The accessions were collected from the worldwide distribution of apricot species to cover the broadest genetic diversity possible. The strategy was to select accessions reflecting the local variability in each country, excluding accessions derived from breeding programs. Using passport data, we discerned seven geographical groups from the East to the West: Group 1 “Eastern Asia,” Group 2 “Central Asia,” Group 3 “Irano-Caucasian,” Group 4 “Continental Europe,” Group 5 “Mediterranean Europe,” Group 6 “North Africa,” and Group 7 “America.” Detailed information on the 890 accessions used in this study is provided in Additional File S1.

Genomic DNA was extracted following the protocol described in Bourguiba et al. (2012b).

### Microsatellite Genotyping

The 890 apricot accessions were genotyped using a set of 25 neutral SSR markers (Cipriani et al., 1999; Testolin et al., 2000; Aranzana et al., 2002; Dirlewanger et al., 2002; Yamamoto et al., 2002; Hagen et al., 2004), which were selected according to their location on the *Prunus* reference genetic map and the ease of amplification in apricot species. Microsatellite markers were amplified with the same multiplex panels and thermal profiles as described by Bourguiba et al. (2012b). Fragment analysis and sizing were carried out using GeneMapper v3.7 software (Applied Biosystems, Foster City, CA, United States). When SSR marker data were already available and obtained at different laboratories, SSR allele sizes were carefully adjusted between collections, both by use of reference accessions known to be common between collections and by re-genotyping a subset of accessions with the full set of 25 SSR markers to confirm the allele adjustment. MICROCHECKER v2.2.3 software (Van Oosterhout et al., 2004) was used to estimate the proportion of null alleles at each locus and for each geographical group.

### Genetic Diversity Analysis

Summary statistics were obtained for each SSR marker using POPGENE v1.32 software (Yeh et al., 1999) to estimate the number of alleles (N_A_), the number of effective alleles (N_E_), the major allele frequency, and the Shannon’s Information index (I). The observed (H_O_) and expected (H_E_) heterozygosities, and the population inbreeding coefficient F_IS_ were calculated using the GENETIX v4.05 program (Belkhir et al., 2004). F_IS_ values were also verified with the program GENEPOP v4.0 (Raymond and Rousset, 1995b; Rousset, 2008) according to the formula of Weir and Cockerham (1984). The software FSTAT v2.9.3 (Goudet, 2003) was applied to compute the allelic richness (A_R_) after scaling down to the smallest partitioning level to avoid a group size-dependent bias on results.

### Genetic Structure Analysis

The model-based Bayesian clustering method implemented in the software package STRUCTURE v2.3.4 (Pritchard et al., 2000) was applied to infer the ancestral population structure. Ten independent replicate runs of STRUCTURE were carried out by setting the number of clusters (*K*) from 1 to 10. Each run consisted of a burn-in period of 100,000 steps followed by 1,000,000 Markov Chain Monte Carlo (MCMC) iterations, assuming an admixture model and correlated allele frequencies. No prior information was used to define the clusters. For the choice of the most likely number of clusters (*K*) supported for our dataset, the plateau criterion proposed by Pritchard et al. (2000), and the Δ*K* method, described by Evanno et al. (2005) and implemented in STRUCTURE HARVESTER v.0.6.93 website (Earl and VonHoldt, 2012) were used. To obtain optimal alignment of the independent runs, CLUMPP v1.1 software (Jakobsson and Rosenberg, 2007) was used with greedy algorithms, 10,000 random input orders and 10,000 repeats, to calculate the average pairwise similarity (*H’*) of runs. CLUMPP output was used directly as input for DISTRUCT v1.1 (Rosenberg, 2004) in order to graphically display the results. Accessions with probability of membership greater than 80% were assigned to corresponding clusters; otherwise they were considered as “admixed.”

To illustrate the genetic structure revealed by the Bayesian model-based clustering, an unweighted Neighbor-Joining tree constructed using a simple matching dissimilarity matrix and bootstrap values over 1000 replicates and a multivariate Principal Coordinate Analysis (PCoA) were assessed using the DARWin software package v6.0.14 (Perrier and Jacquemoud-Collet, 2006).

In order to take into account the historical admixture events between the identified clusters, we adopted the tree-based approach implemented in TreeMix v1.13 (Pickrell and Pritchard, 2012). TreeMix software models the relationship between the sample populations and their ancestral population using genome-wide allele frequency data and a Gaussian approximation of genetic drift. Migration (M) can subsequently be fitted between populations that fit poorly to the tree model and for which the admixture is inferred. Finally, the M value that reached an asymptote and simultaneously provided the smallest residual variance was selected as the most predictive model.

### Genetic Differentiation Analysis

Summary statistics were calculated for the material grouped according to the geographic regions of origin as well as for each cluster identified by the model-based Bayesian clustering method, including the mean number of alleles per locus (N_A_), number of effective alleles (N_E_), number of private alleles (i.e., those only found in one level), number of unique alleles (i.e., those only detected in one unique accession), allelic richness (A_R_), observed heterozygosity (H_O_), expected heterozygosity (H_E_), and inbreeding coefficient (F_IS_).

Pairwise F_ST_ estimates for the different partitioning levels considered in each case were obtained using the GENEPOP v4.0 program, and Fisher’s method was applied to test the significance of obtained values by running 1000 permutations (Raymond and Rousset, 1995a). Pairwise standard genetic distances of Nei (1972) between apricot geographic groups were computed and used to conduct cluster analysis with the Neighbor-joining algorithm and to construct an unrooted tree with 1000 bootstraps over SSR loci as implemented in PHYLIP v3.69 program package (Felsenstein, 2008). Analysis of molecular variance (AMOVA) implemented in the GenAlEx v6.503 program (Peakall and Smouse, 2012) was conducted to estimate hierarchical differentiation at two levels: (*i*) the apricot geographic groups according to the origin of the material; and (*ii*) the genetic clusters defined by STRUCTURE analysis.

### Searching for Evidence of a Recent Bottleneck

In recent populations subjected to a bottleneck, observed heterozygosity is higher than expected given the number of alleles in a population if this population is at the mutation-drift equilibrium (Cornuet and Luikart, 1996). This heterozygosity excess was used to test for the genetic signature of bottlenecks in apricot as implemented in BOTTLENECK software v1.2.02 (Piry et al., 1999). Gene diversity was estimated with 1,000 iterations under three models of molecular evolution: the stepwise mutation model (SMM), the infinite allele model (IAM), and the two-phase mutation model (TPM). The TPM has been shown to deliver the most realistic results for the typical mutational events of microsatellite loci (Di Rienzo et al., 1994). We used TPM with 95% single-step mutations and 5% multiple-step mutations and a variance among multiple steps of 12, as recommended by Piry et al. (1999) for microsatellite data. Significance was tested using the one-tailed Wilcoxon signed rank test. A qualitative test of model shift was also performed to calculate the allele frequency distribution using BOTTLENECK v1.2.02. In fact, bottlenecks cause alleles at low frequency to become less abundant than alleles in one or more intermediate allele frequency classes, thus shifting the mode of the normally L-shaped frequency distribution into higher frequency classes (Luikart et al., 1998). All input file preparations were prepared using CONVERT v1.31 (Glaubitz, 2004).

### Demographic Modeling

A combination of coalescent-based simulation of the evolutionary processes (Hudson, 1990) and Approximate Bayesian Computing (Beaumont et al., 2002), as implemented in the DIYABC v2.1.0 software (Cornuet et al., 2014), was used to estimate the demographic parameters of the historical processes identified through the TreeMix inference. We applied the approach of Barthe et al. (2017), and aimed to estimate ratios of current to past effective population sizes, instead of attempting to estimate absolute values. As in Barthe et al. (2017), instead of comparing scenarios we left the parameters free to vary in a single model, and used parameter posterior distributions to infer the direction of demographic processes. We focused on the estimation of ratios of effective population sizes for the five clusters and of admixture proportions. The parameters applied to DIYABC are described in Additional File S2.

### Implementation of the Core Collection

The development of the core collection was established based on the genotyping data using the advanced maximization strategy (M), implemented by modifying the heuristic algorithm in the PowerCore software program as described by Kim et al. (2007). The advanced maximization strategy M selects the most diverse accessions to represent the total variability of the entire collection. The PowerCore software program minimizes allele loss and therefore effectively selects the most diverse accessions, reducing the number of redundant accessions as described by Kim et al. (2007).

## Results

### SSR Polymorphism

Summary statistics of the 25 microsatellite loci over the 890 apricot accessions are listed in Table 1. A total of 609 alleles were detected across the 25 loci, with the number of alleles per locus ranging from 18 (AMPA119 and UDP98-412) to 32 (CPPCT022). The average number of alleles per locus was 24.36. The effective number of alleles per locus varied from 1.427 (UDP96-018) to 8.668 (UDP98-409), with a mean value of 4.643. The allelic richness ranged from 7.115 (Ma014a) to 15.582 (CPPCT022). The major allele showed a highly variable frequency from one locus to another, ranging from 0.192 (UDP98-409) to 0.835 (UDP96-018). The highest Shannon information index was 2.516 at the locus CPPCT022, and the lowest was 0.840 at the locus UDP96-018. The average observed and expected heterozygosities across markers were 0.570 and 0.732, respectively. All SSR loci displayed significant heterozygosity deficit (*P* < 0.0001; Table 1).

**TABLE 1:** Summary statistics of genetic variation at 25 SSR loci in the entire apricot germplasm collection.

**Locus**	**Linkage group position**	**N_A_**	**N_E_**	A_R_*	**Major allele frequency**	**I**	**H_O_**	**H_E_**	F_IS_**
AMPA100^a^	6	21	6.008	12.067	0.311	2.155	0.725	0.834	0.129
AMPA101^a^	3	23	3.648	9.636	0.470	1.755	0.606	0.726	0.164
AMPA105^a^	5	29	7.083	12.555	0.204	2.258	0.615	0.859	0.283
AMPA109^a^	1	27	2.381	11.008	0.636	1.551	0.336	0.580	0.420
AMPA116^a^	2	21	5.127	10.284	0.374	2.006	0.639	0.805	0.206
AMPA119^a^	3	18	2.098	7.128	0.658	1.167	0.385	0.523	0.263
BPPCT001^b^	2	23	2.321	7.917	0.596	1.259	0.471	0.569	0.171
BPPCT004^b^	2	28	7.799	14.43	0.245	2.418	0.699	0.872	0.198
BPPCT008^b^	6	25	6.018	11.369	0.298	2.127	0.707	0.834	0.151
BPPCT017^b^	5	29	3.507	11.152	0.458	1.772	0.585	0.715	0.181
BPPCT025^b^	6	20	3.375	7.117	0.465	1.520	0.564	0.704	0.198
BPPCT030^b^	2	21	5.649	9.49	0.209	1.947	0.703	0.823	0.145
BPPCT038^b^	5	21	5.749	11.247	0.277	2.078	0.697	0.826	0.156
BPPCT040^b^	4	23	2.847	8.624	0.516	1.471	0.525	0.649	0.190
CPPCT006^c^	8	30	6.899	13.411	0.284	2.312	0.766	0.855	0.104
CPPCT022^c^	7	32	8.630	15.582	0.211	2.516	0.698	0.884	0.210
CPPCT030^c^	6	29	4.816	12.577	0.408	2.102	0.645	0.792	0.185
CPPCT033^c^	7	21	3.150	9.828	0.534	1.689	0.500	0.683	0.267
CPPCT034^c^	1	20	2.868	8.692	0.536	1.506	0.509	0.651	0.217
Ma014a^d^	6	21	2.575	7.115	0.519	1.276	0.464	0.612	0.240
Ma040a^d^	6	27	4.266	9.54	0.368	1.816	0.465	0.766	0.392
UDP96-018^e^	1	23	1.427	7.445	0.835	0.840	0.132	0.299	0.557
UDP97-402^e^	4	28	4.274	12.082	0.426	1.978	0.436	0.766	0.430
UDP98-409^e^	8	31	8.668	15.273	0.192	2.508	0.742	0.885	0.161
UDP98-412^f^	6	18	4.884	9.651	0.336	1.887	0.643	0.795	0.191
Multilocus average		24.36	4.643	−	–	1.837	0.570	0.732	0.221

*N*_*A*_, *Observed number of alleles; N_*E*_*,*Effective number of alleles; A_*R*_*, *Allelic Richness; I, Shannon’s Information index; H_*O*_*, *Observed Heterozygosity; H_*E*_*, *Expected Heterozygosity; F_*IS*_*, Inbreeding coefficient. *Based on minimum sample size of 33 diploid individuals. **Exact test significant at P-value < 0.0001. Primers developed by: ^*a*^(Hagen et al., 2004), ^*b*^(Dirlewanger et al., 2002), ^*c*^(Aranzana et al., 2002), ^*d*^(Yamamoto et al., 2002), ^*e*^(Cipriani et al., 1999), ^*f*^(Testolin et al., 2000).

### Genetic Diversity and Differentiation Among Geographic Groups

Seven geographical groups from the East to the West were identified using passport data as Group 1 “Eastern Asia,” Group 2 “Central Asia,” Group 3 “Irano-Caucasian,” Group 4 “Continental Europe,” Group 5 “Mediterranean Europe,” Group 6 “North Africa,” and Group 7 “America.” A comparative analysis of their genetic diversity indicated that the material issued from the center of origin showed the most elevated level of genetic diversity. In fact, “Central Asia” was the group identified as having the highest averages for the following diversity measures: number of alleles per locus (N_A_ = 19.88), number of effective alleles per locus (N_E_ = 7.694), number of private alleles (97), number of unique alleles (66), allelic richness (A_R_ = 13.752) and observed and expected heterozygosity values (H_O_ = 0.617 and H_E_ = 0.852, respectively) (Table 2). In contrast, the “North Africa” group displayed the lowest average number of alleles per locus (N_A_ = 7.8), allelic richness (5.412), and observed and expected heterozygosity values (H_O_ = 0.495 and H_E_ = 0.572), while the “Continental Europe” group exhibited the lowest mean number of effective alleles per locus (N_E_ = 2.898), and no private and unique alleles were encountered. Except for the “North Africa” and “America” groups, all groups displayed a significant heterozygosity deficit (*P* < 0.01; Table 2).

**TABLE 2:** Comparison of genetic diversity generated by 25 SSR markers within the seven geographic apricot groups.

**Geographic group**	**Sample size**	**Allele number**				A_R_***	**H_O_**	**H_E_**	**F_IS_**
		**N_A_**	**N_E_**	Private*	Unique**				
Group 1: Eastern Asia	67	15.88	6.726	42	23	13.316	0.616	0.835	0.269****
Group 2: Central Asia	142	19.88	7.694	97	66	13.752	0.617	0.852	0.279****
Group 3: Irano-Caucasian	86	12.04	4.253	8	7	9.395	0.578	0.708	0.201****
Group 4: Continental Europe	86	10	2.898	0	0	7.701	0.606	0.614	0.064****
Group 5: Mediterranean Europe	250	11.4	3.190	13	6	6.573	0.580	0.636	0.094****
Group 6: North Africa	214	7.8	2.904	5	2	5.412	0.495	0.572	0.129
Group 7: America	45	9.36	4.245	8	4	8.773	0.588	0.713	0.185

*N*_*A*_, Mean number of alleles per locus; N_*E*_, Effective number of alleles; A_*R*_, Allelic Richness; H_*O*_, Observed Heterozygosity; H_*E*_, Expected Heterozygosity; F_*IS*_, Inbreeding coefficient. *Alleles only found in one group, **alleles only detected in one unique accession, ***based on minimum sample size of 33 diploid individuals, ****exact test significant at P-value < 0.01.

The pairwise population differentiation values (F_ST_) among the seven apricot geographic groups were all highly significant and within the same range (0.032–0.187) (Additional File S3). They indicated a strong differentiation between “Eastern Asia” and “North Africa” groups (F_ST_ = 0.187). However, a low differentiation was observed between the “America” group and both “Irano-Caucasian” and “Mediterranean Europe” groups (F_ST_ = 0.032 and F_ST_ = 0.036, respectively) as well as between the “Mediterranean Europe” and “North Africa” groups (F_ST_ = 0.038). Similar results were obtained when computing pairwise genetic distances among the seven apricot geographic groups (Additional File S3). The Neighbor-Joining tree obtained showed a pattern according to the worldwide geographic distribution of the groups from the East to the West (Additional File S4). The “Eastern Asia” group was the most distant from the other groups, while the “Mediterranean Europe” and “North Africa” groups enclosed the lowest genetic distance value reflecting their close genetic basis. Moreover, the “America” group presented an intermediate position between the Asian and the European groups.

### Genetic Structure Analysis

**FIGURE 1:**
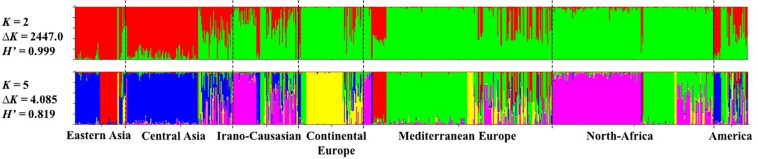
Inference of population structure based on 25 SSR markers using STRUCTURE program for *K* = 2 and *K* = 5. Each individual is represented by a single vertical line, which is partitioned into colored segments in proportion to the estimated membership in the K clusters. The seven apricot geographic groups are separated by a black line. H’ represents the similarity coefficient between ten runs for each K, and ΔK is the *ad hoc* measure of Evanno.

The model-based Bayesian clustering approach implemented in STRUCTURE software was used to elucidate the genetic structure of worldwide apricot germplasm. The change rate in the log-likelihood between successive *K* values (Δ*K*) revealed two levels of clustering at *K* = 2 (Δ*K* = 2447.0) and *K* = 5 (Δ*K* = 4.085; Figure 1 and Additional File S5). Based on the average similarity of individual assignments across runs (*H’*) generated by CLUMPP for the 10 runs, similar results were obtained as the highest similarity coefficient (*H’*) was observed for *K* = 2 (*H’* = 0.999) and *K* = 5 (*H’* = 0.819; Figure 1).

For the first level of clustering at *K* = 2, the apricot accessions were differentiated into two clusters, the first one consisted of accessions from Asia (i.e., “Eastern Asia” and “Central Asia” groups) and some accessions from the “America” group, while the second cluster included all the remaining accessions (Figure 1).

We used the second level of clustering at *K* = 5 to define the clusters considered in subsequent analyses (Figure 1). For analyses hereafter, genotypes were assigned to a given cluster if their membership coefficient for that cluster was *qI* ≥ 0.8 (Additional File S6). Cluster 1 (44 accessions; *in red* on Figure 1) included accessions from the “Eastern Asia” group and specifically those originating from Japan. Cluster 2 (149 accessions; *in blue*) grouped accessions from “Central Asia” and China. Cluster 3 (163 accessions; *in pink*) grouped accessions from the “Irano-Caucasian” group as well as the seed propagated accessions from “North Africa.” Cluster 4 (64 accessions; *in yellow*) comprised accessions from the “Continental Europe” group. Finally, Cluster 5 (170 accessions; *in green*) included accessions from the “Mediterranean Europe” group and some graft-propagated accessions from “North-Africa” (Figure 1 and Additional File S7). In total, 590 accessions (66.29%) were strongly assigned to a cluster (Additional File S7). The remaining 300 accessions were assumed to have admixed ancestry (i.e., *qI* < 0.8). We noted that the “America” geographical group enclosed the highest number of admixed accessions (27/45 accessions; 60%), for what genotypes that were mainly composed by Cluster 5 (18 accessions) and Cluster 2 (12 accessions).

**FIGURE 2:**
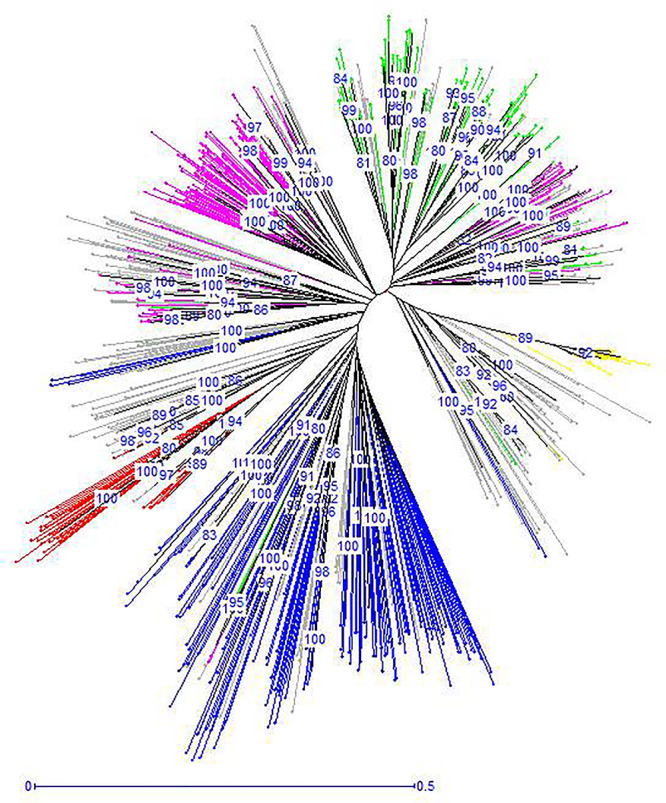
Neighbor-joining dendrogram based on simple matching dissimilarity matrix showing relationships among the 890 apricot accessions. The five clusters identified by STRUCTURE analysis are depicted using the color codes as defined in Figure 1 with Cluster 1 in red, Cluster 2 in blue, Cluster 3 in pink, Cluster 4 in yellow, and Cluster 5 in green. The mixed accessions are in gray. Bootstrap values above 80% are shown.

**FIGURE 3:**
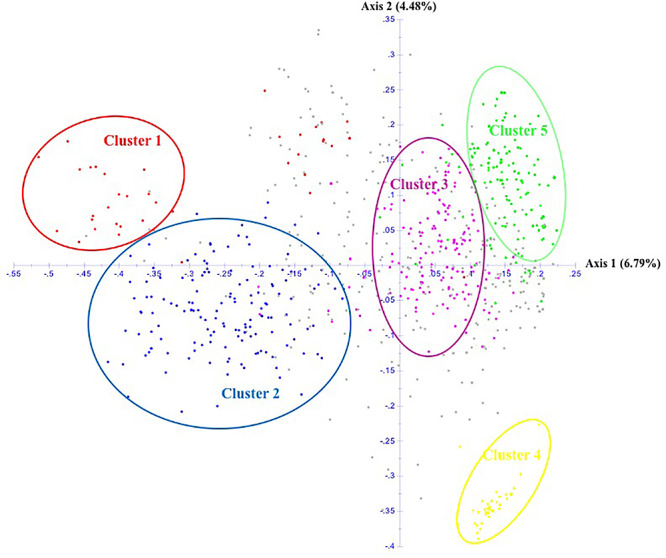
Scatter plot of the Principal Coordinate Analysis of 890 apricot accessions based on 25 SSRs. The five clusters are depicted using the color codes as defined by STRUCTURE analysis with Cluster 1 in red, Cluster 2 in blue, Cluster 3 in pink, Cluster 4 in yellow, and Cluster 5 in green. The mixed accessions are in gray.

The unrooted Neighbor-Joining tree (NJ) of the 890 accessions based on dissimilarity scores as well as the multivariate Principal Coordinate Analysis (PCoA) revealed a similar pattern as inferred with the model-based Bayesian clustering analysis, with a clear genetic discrimination of the five clusters (Figures 2, 3). Both analyses revealed that Cluster 2 was the most diversified cluster. In addition, the PCoA plot showed that the second axis differentiated both Clusters 1 and 2 including material from “Eastern Asia” and “Central Asia” groups, from the remaining clusters. Overall, the observed cluster distribution reflected the geographic origin of the material with an overlap between Cluster 3 and Cluster 5 (Figure 3).

**FIGURE 4:**
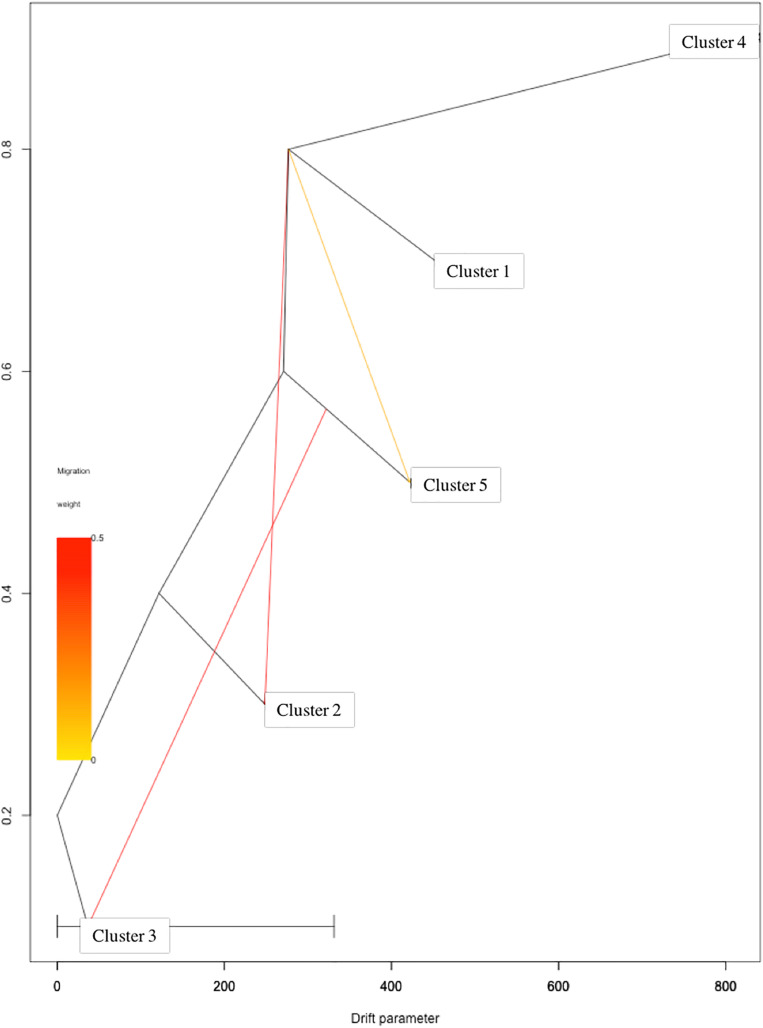
TreeMix analysis with three migration events. Clusters in the graph corresponded to the five genetic clusters identified by STRUCTURE analysis.

Based on log-likelihood and residual variance values, the most predictive model suggested the presence of three migration events (Figure 4). A first migration event was predicted by TreeMix software from Cluster 1 (Eastern Asia) toward Cluster 2 (Central Asia) with the highest weight of 0.452. A second migration event (0.451) was directed from Cluster 5 (Mediterranean Europe) to Cluster 3 (Irano-Caucasian). Finally, the third migration event occurred between Cluster 1 (Eastern Asia) to Cluster 5 (Mediterranean Europe) with a weight of 0.1.

### Genetic Diversity and Differentiation Among Genetic Clusters

Genetic statistics were computed for the five identified genetic clusters (Table 3). Cluster 2 enclosed the highest values of total allele number (427), mean number of alleles per locus (N_A_ = 17.08), effective number of alleles (N_E_ = 6.680), private alleles (163), unique alleles (45), allelic richness (A_R_ = 10.911) and expected heterozygosity (H_E_ = 0.813). In contrast, Cluster 4 displayed the lowest values of total allele number (82), mean number of alleles per locus (N_A_ = 3.28), effective number of alleles (N_E_ = 1.707), allelic richness (A_R_ = 2.540), and expected heterozygosity (H_E_ = 0.360). In addition, Cluster 4 included only one private allele and no unique allele (Table 3). For the different estimators measured, the genetic diversity levels were moderate and relatively homogeneous within Clusters 1, 3 and 5. Among the five clusters, only Clusters 2 and 5 exhibited a significant heterozygosity deficit, while Cluster 4 showed a significant heterozygosity excess (*P* < 0.05; Table 3).

**TABLE 3:** Descriptive information for each of the five clusters of genotypes identified by STRUCTURE analysis.

**Cluster**	**Sample size**	**Allele number**					A_R_***	**H_O_**	**H_E_**	**F_IS_**
		**Total**	**N_A_**	**N_E_**	Private*	Unique**				
Cluster 1	44	194	7.76	3.478	14	8	6.691	0.656	0.676	0.031
Cluster 2	149	427	17.08	6.680	163	45	10.911	0.614	0.813	0.252****
Cluster 3	163	198	7.92	2.908	11	4	5.032	0.482	0.554	0.122
Cluster 4	64	82	3.28	1.707	1	0	2.539	0.599	0.360	-0.474****
Cluster 5	170	182	7.28	2.592	7	6	4.498	0.538	0.560	0.051****

N_*A*_, Mean number of alleles per locus; N_*E*_, Effective number of alleles; A_*R*_, Allelic Richness; H_*O*_, Observed Heterozygosity; H_*E*_, Expected Heterozygosity; F_*IS*_, Inbreeding coefficient. *Alleles only found in one cluster, **alleles only detected in one unique accession, ***based on minimum sample size of 22 diploid individuals, ****exact test significant at P-value ≤ 0.05.

The genetic differentiation among the five defined clusters was high since the average value was F_ST_ = 0.174, suggesting a strong worldwide genetic structure for this species. Pairwise F_ST_ among the five apricot clusters were all highly significant (*P* < 0.0001; Table 4). They indicated a high differentiation between Cluster 4 and all other clusters (0.238–0.364), whereas a low differentiation was found between Cluster 3 and Cluster 5 (F_ST_ = 0.110). Similar results were obtained when computing the pairwise genetic distances among the five genetic clusters (Table 4).

**TABLE 4:** Pairwise F_*ST*_ (above diagonal) and Nei’s genetic distances (below diagonal) values among the five genetic clusters.

**Cluster**	**1**	**2**	**3**	**4**	**5**
1	–	0.114	0.212	0.364	0.206
2	0.500	–	0.139	0.238	0.152
3	0.526	0.341	–	0.258	0.110
4	0.804	0.536	0.366	–	0.257
5	0.520	0.415	0.173	0.369	–

**Global F_*ST*_ = 0.174; All pairwise F_*ST*_ values were highly significant at P < 0.0001.**

Accordingly, the two-level AMOVA showed that most genetic variation resided within clusters (83%) as compared with variation among clusters (17%). Regarding the geographic groups, the genetic variation within groups was 92%, while the genetic variation among groups was 8% (Table 5).

**TABLE 5:** Analysis of Molecular Variance based on the 25 SSR markers of studied apricot germplasm.

**Populations**	**Source of variation**	***df***	**Sum of squares**	**Estimated variability**	Percentage of variation*
Geographic origin based	Among groups	6	1157.597	0.766	8
	Within groups	1773	14671.668	8.275	92
	Total	1779	15827.265	9.041	100
STRUCTURE analysis based	Among clusters	4	1472.179	1.608	17
	Within clusters	1175	9163.726	7.799	83
	Total	1179	10635.904	9.407	100

Df, degrees of freedom. *P-value = 0.001 based on 999 permutations.

### Evidence for Domestication Bottleneck

**FIGURE 5:**
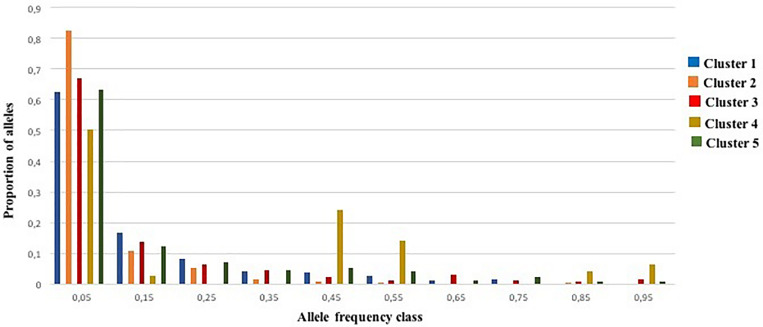
Allele frequency distribution for 25 SSR loci within the five genetic clusters. Bars represent the proportion of alleles found in each allele frequency class. The distribution is L-shaped, as expected for a stable population under mutation-drift equilibrium.

An excess of heterozygosity is an indicator of recent bottleneck, while a deficit as a result of inbreeding is a sign of expansion. Both represent departures from a Hardy-Weinberg equilibrium. For the BOTTLENECK analysis, Wilcoxon signed rank tests were not significant under both TPM (with 95% single-step mutations) and SMM mutation models; only the test based on the IAM identified a heterozygosity excess for Cluster 1 and Cluster 2 (*P* < 0.05; Additional File S8). The absence of a heterozygosity excess under both the SMM and the TPM models suggested no recent genetic bottleneck within any of the identified genetic clusters in the recent past. Except for Cluster 4, these results were also consistent with the normal L-shaped distribution of allele frequencies as there is no evidence for a significant deviation as expected for a stable population under mutation-drift equilibrium (Figure 5). For Cluster 4, the graphic test exhibited a particular distribution of the allele proportions with a decrease in the low frequency followed by an increase in the intermediate frequency class (e.g., 0.4–0.5) predicting a population bottleneck as shown in Figure 5.

### Estimation of Parameters in Domestication History

**FIGURE 6:**
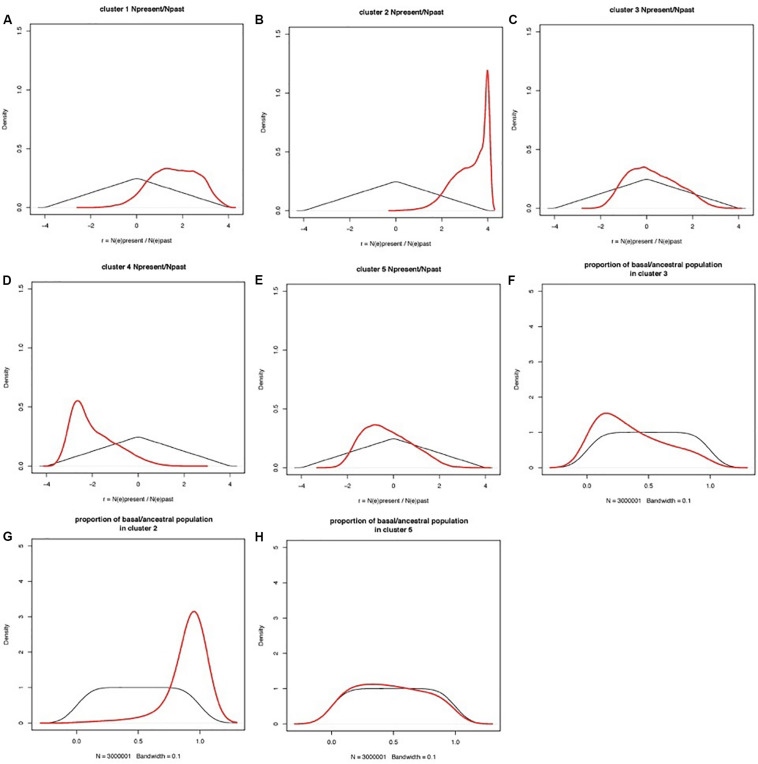
Prior and posterior distributions of evolutionary parameters estimated by coalescent-ABC modeling in DIYABC. Clusters are defined by STRUCTURE analysis as in Figure 1. **(A–E)** Prior and posterior distributions of r = N_E(present)_/N_E(past)_ for each cluster, with N_E(present)_ = current effective population size, N_E(past)_ = effective population size before the demographic change. r is shown on a log(10) scale, so that a posterior peak at zero indicated no demographic change, a posterior peak at negative values suggests a population bottleneck (i.e., current population smaller than past population), and a posterior peak at positive values suggests a population expansion (i.e., current population larger than past population). **(F–H)** Population admixture ratios. Thin black lines: prior distributions; thick red lines: posterior distributions.

Coalescent modeling and Approximate Bayesian Computing (ABC) were used to infer the evolutionary parameters associated with the domestication history of cultivated apricot. Empirical summary statistics fitted the cloud of simulated summary statistics (Additional File S2). Ratios of present-to-past effective population size had a clear peak for most clusters, distinct from the prior (Figure 6). Population size ratio *r* showed moderate signs of expansion for Cluster 1, and much stronger expansion for Cluster 2; a bottleneck for Cluster 4, and quasi-stability or slight contraction for both Clusters 3 and 5. Population admixture showed that Cluster 2 is mostly made of lineages derived from the basal/ancestral population (i.e., introgression stemming from the root of phylogenetic tree), Cluster 3 received little introgression from basal/ancestral germplasm, and Cluster 5 returns no signal (i.e., posterior overlaps prior; the data are not informative). Estimations of the timings of events are not reported here, because they are scaled to mutation rates (also inferred in the model) and because they are constrained by the order of events in the phylogenetic tree, thus making the posteriors hard to interpret.

### Apricot Core Collection Development

To conserve an overview for the whole genetic diversity of the studied germplasm, a core collection that contained 34.38% (306 accessions) of the 890 apricot accessions was constructed capturing 100% of the total alleles (Additional File S1). The core collection included 55 accessions from Group 1 “Eastern Asia,” 105 accessions from Group 2 “Central Asia,” 33 accessions from Group 3 “Irano-Caucasian,” 15 accessions from Group 4 “Continental Europe,” 34 accessions from Group 5 “Mediterranean Europe,” 41 accessions from Group 6 “North Africa,” and finally 23 accessions from Group 7 “America.”

Regarding the identified genetic clusters, the constructed core collection was mainly composed by accessions from Cluster 2 (122 accessions) and the admixed accessions (111 accessions), followed by accessions from Clusters 3 (36 accessions), Cluster 1 (24 accessions), Cluster 5 (11 accessions), and Cluster 4 (only 2 accessions).

## Discussion

### Genetic Diversity and Structure of Worldwide Apricot Germplasm

The current study used nuclear SSR genotyping to analyze the genetic diversity and structure of cultivated apricot accessions from a worldwide collection (Japan to North America) in order to clarify the demographic history of diffusion of this species from its center of origin.

The 890 accessions revealed a total of 609 alleles with a mean value of 24.36 alleles per locus. It highlights the highest level of diversity ever described within a worldwide apricot collection, compared with results obtained in previous studies focusing on apricot diversity at a large scale. In fact, Zhebentyayeva et al. (2003) used 14 SSR markers to investigate patterns of genetic variation in 74 apricot accessions representing the European, Irano-Caucasian, Chinese and Central Asian regions and revealed an average of 7.64 alleles per locus. Pedryc et al. (2009) evaluated the genetic diversity of 77 apricot accessions belonging to five different geographical groups (China, Asia, North America, Mediterranean and Western Europe as well as Middle Europe) using 6 SSR markers and identified 11.83 alleles per locus. Similarly, using the same set of SSR markers but focusing only on the Mediterranean apricot germplasm, Bourguiba et al. (2012b) highlighted an average of 10.28 alleles per locus. Hence, the widening of the geographical scale of this study through the addition of germplasm from Eastern Asia region for the first time allowed us to cover the broadest range of genetic diversity within the cultivated apricot germplasm. When comparing the obtained genetic diversity with previously reported genetic diversity from wild apricot germplasm, results revealed also that the diversity issued from our characterized germplasm is higher. In fact, an average number of 23 alleles per locus was obtained studying the genetic variability of 81 wild apricots from West China using 8 SSR markers (He et al., 2007). Decroocq et al. (2016) investigated the level of diversity of 230 wild trees from Central Asia and 142 cultivated apricots as representatives of the worldwide cultivated apricot germplasm with 15 SSR markers and found an average value of 16.75 alleles per locus. The genetic diversity of wild *P. armeniaca* and closely related species was involved in apricot domestication in Central and Eastern Asia where self-incompatible cultivars prevailed. In combination with seed-propagation, genetic diversity of apricot germplasm was largely preserved at early stages of domestication and it can be related to the difficulties in apricot grafting propagation in comparison with plum, apple and peach species. Similarly, moderate loss of genetic diversity in cultivated Chinese cherry was also evidenced by Zhang et al. (2018) and related to the existence during long-term cultivation history of both seed and grafting propagation.

Thus, we can conclude that a large part of the cultivated apricot genetic diversity was captured in the already available collections. The collaboration within the network of repositories associated with this study enabled us to capture the largest part of the existing variability due to the complementarity of the worldwide collections. The high level of diversity observed in the repositories located worldwide compared with the one observed in the center of origin and in the primary diversification centers is also observed in tomato where huge differences are observed between sub-species in America each of them being strongly adapted to their particular region of development (Razifard et al., 2020).

Overall, we revealed that apricot accessions around the world clustered into five clearly differentiated gene pools. Strong relationships between memberships of accessions within the clusters defined by STRUCTURE and their geographical regions of origin were identified. Similar results of the association between the genetic structure and the geographic origin of the material were also reported in other fruit crops such as date palm (Zehdi-Azouzi et al., 2015), and olive (Haouane et al., 2011).

Accessions from China and the “Central Asia” geographic group were clustered together (Cluster 2) exhibiting the highest level of genetic variability characterized by a higher allelic richness and expected heterozygosity values. In addition, this cluster (Cluster 2), which was the most represented in the constructed core set, enclosed a unique variability with 163 private alleles and 45 unique alleles supporting the notion that China and Central Asia represented two primary centers of origin for apricot species. We confirmed the results reported by Decroocq et al. (2016) with the occurrence of specific alleles within the Chinese germplasm. These results were also consistent with previous studies suggesting that the total number of alleles and the number of unique alleles were among the highest in the Chinese apricot population (Zhebentyayeva et al., 2003; Pedryc et al., 2009).

Considering the others clusters, the presence of private alleles for Cluster 1 (14 private alleles), Cluster 3 (11 private alleles) and Cluster 5 (7 private alleles) could be associated with the existence of different centers of diversification in the related geographical regions following the apricot diffusion from its center of origin mediated by the main civilizations. In fact, it was accepted in several studies that the Irano-Caucasian region represented here by Cluster 3 was considered a secondary zone of apricot diversification (Vavilov, 1951; Faust et al., 1998). Moreover, the appearance of a distinctive cluster in Eastern Asia which included Japanese apricots (Cluster 1) could have represented another secondary center of apricot diversification resulting from the selection of accessions of ornamental interests in this region and/or specific adaptation to warmer and humid climatic conditions. Finally, apricots from the Mediterranean Europe region (Cluster 5) cultivated exclusively for fresh fruits also constituted a secondary center of apricot diversification. Thus, despite the importance of the center of origin in terms of genetic variability, our results proved that three others zones of apricot diversification (Clusters 1, 3, and 5), which were approximately equally represented in the core set, were important and complementary to evaluate the worldwide pattern of genetic diversity of apricot species. These zones of apricot diversification could offer new insights for challenging the genes involved in the processes of adaptation to climatic changes, which would be particularly useful for apricot species tremendously characterized by a narrow adaptive range.

The evidence for a genetic bottleneck in the five identified genetic clusters was ambiguous. When testing either the extreme model or the in-between model (TPM with 95% of single-step mutations), evidence is lacking for the case of a recent bottleneck in apricot diffusion around the world. In fact, Cornuet and Luikart (1996) noted that bottlenecks can go undetected if they were either not very severe or were very recent. Moreover, except for Cluster 4, the analysis of allele frequency distribution failed to detect a mode-shifted distribution of allele frequencies, also suggesting that a bottleneck is not likely to have occurred in the recent past. Regarding Cluster 4, the low level of private alleles and the absence of unique allele could be explained by a diversification related to adaptive characteristics or a bottleneck effect. Compared with other *Prunus* species, there was also no evidence of bottlenecks in any populations of *Prunus lannesiana* in Izu Islands in Japan under the IAM, SMM or TPM assumptions (Kato et al., 2011), while, an excess of heterozygosity in a core collection of sweet cherry landraces under the TPM model has been found to be related to a genetic bottleneck (Campoy et al., 2016). Moreover, recently, significant genetic bottlenecks were also suggested in cultivated Chinese cherry during domestication (Zhang et al., 2018).

### Demographic History of Apricot Species

The demographic history of crop domestication has been recently assessed using microsatellite markers and model-based Bayesian clustering method in sweet cherry (Mariette et al., 2016), olive (Besnard et al., 2013a), date palm (Zehdi-Azouzi et al., 2015), and apple (Urrestarazu et al., 2016).

Before revealing the history of diffusion routes of cultivated apricot species, it is important to confirm the original center of origin. Overall, our study provided strong support that the region of Central Asia including China constituted the center of origin of apricot species which included a high and particular genetic diversity that can be considered as a reservoir of potentially interesting genes to sustain modern breeding programs. For the Central Asian cluster, the ABC method revealed that the apparent increased effective population size may be the consequence of the massive introgression of highly diverse wild material in the cultivated gene pool which would have raised genetic diversity as also suggested by Liu et al. (2019).

**FIGURE 7:**
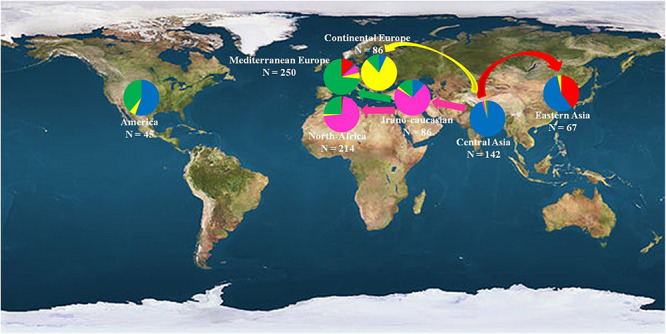
Geographical distribution of the 890 investigated apricot accessions classified into the seven geographic origins. Color codes denotes the clusters identified by STRUCTURE analysis as reported in Figure 1, with Cluster 1 in red, Cluster 2 in blue, Cluster 3 in pink, Cluster 4 in yellow, and Cluster 5 in green.

These multiple domestication events have already been suggested in different fruit species such as olive (Besnard et al., 2013b), grapevine (Myles et al., 2011), and cherry (Zhang et al., 2018). Human efforts have resulted in the domestication of several fleshy-fruited species, increasing the sizes and sweetness of the fruits (Xiang et al., 2016) and based on specific adaptive traits. The hypothesis of different diffusion routes and the existence of secondary domestication centers have thus been deeply and thoroughly studied in the present work (Figure 7).

The first diffusion route was through the Eastern Asia to Japan (Cluster 1) where apricots present low chilling requirements. Apricot was first introduced to Japan from China about 2,000 years ago. However, in contrast to other introduced fruit species such as pear (Zohary et al., 2012) or persimmon, agricultural cultivation of apricot was initiated during the early modern period and was located only in a narrow region of Central Japan. Before that time, apricot was cultivated exclusively as ornamental or medicinal plants. Moreover, the fact that accessions from Cluster 1 were also encountered only in Southern European countries like France and Italy, and regarding the subsequent number of admixed accessions between Clusters 1 and 5 in the “Mediterranean Europe” group (31/104 accessions; 29.8%), we can suggest the hypothesis of an apricot dissemination by boat directly from Japan to the Mediterranean Europe countries. This hypothesis was confirmed by the TreeMix analysis with the detection of one migration event between Clusters 1 and 5 attesting the occurrence of gene flow between these two regions. Finally, coalescent/ABC modeling suggested that the East Asian and Central Asian germplasm underwent a recent expansion (two orders of magnitude larger for the Central Asian cluster) and a major introgression event from basal/ancestral germplasm, which contributed most of its genes.

The second diffusion route was from the Irano-Caucasian region (Cluster 3) through the Mediterranean countries to Morocco (Cluster 5). Indeed, Bourguiba et al. (2012b) revealed three apricot gene pools throughout the Mediterranean Basin region: “Irano-Caucasian,” “North-Mediterranean Basin,” and “South-Mediterranean Basin,” as well as the existence from the Irano-Caucasian region of two apricot diffusion routes, one through the Southern European countries and the other through the North African countries. Here, there was no evidence for distinction of the North and South Mediterranean Basin gene pools and apricot accessions from the “North-Africa” group were mainly classified within the Cluster 3 (119/166 accessions) representing the Irano-Caucasian region. This result can be explained by the close genetic similarity of apricot in the Mediterranean Basin compared to worldwide genetic diversity and confirmed that apricot from the Mediterranean Basin came from the Irano-Caucasian region as described by Bourguiba et al. (2012b). In addition, focusing on the “North-Africa” group, the fact that some apricot accessions originated from this region were found to be clustered with accessions from the “Mediterranean Europe” group in Cluster 5 (41/166 accessions), as well as the high level of admixed accessions between Clusters 3 and 5 confirming the presence of gene exchange between the Northern and Southern Mediterranean countries as suggested by Bourguiba et al. (2013). These two remaining clusters exhibited moderate signs of contraction with the ABC method, with apparent small introgression from the (unsampled) historical domesticated population and a more stable effective population size, consistent with an history of mixing and sharing of germplasm across regions.

Finally, the third apricot dissemination route was through the Continental European countries (Cluster 4). In fact, apricot accessions from this region, which were self-compatible and specifically adapted to winter frost through high chilling requirements, appeared to be distantly related to the accessions from Mediterranean Europe, confirming previous studies which demonstrated that the Eastern European accessions can be clearly distinguished from other cultivars of European origin (Faust et al., 1998). Plant traits such as mating system have been suggested to affect the evolution of local adaptation, mainly due to their effects on the level and distribution of genetic variation. Self-compatible species tend to be more strongly differentiated at a smaller scale than outcrossing species (Linhart and Grant, 1996) and therefore the former are expected to show stronger adaptation to local conditions. Moreover, accessions from Cluster 4 exhibited the lowest level of genetic variability, probably because most of them belong to a few prominent cultivar groups or they arose from hybridization between them as suggested by Romero et al. (2003) and Pedryc et al. (2009). The ABC method confirmed that a severe population bottleneck occurred, which is compatible with strong subsampling of germplasm, because of limited transfer of material to the continental fringes, and/or because of strong selective pressure in favor of lineages well adapted to cold climates.

Apricot accessions from the “America” group displayed a higher level of admixture (60%) mainly with Cluster 2 and Cluster 5. The assigned accessions (18/45 accessions), they belonged to either cluster 2 (10 accessions) or cluster 5 (7 accessions), suggesting that these two gene pools contributed to the genetic basis of apricot material from America which was recently introduced and thus used through human hybridization. In fact, when studying markers linked to disease resistance in apricot, Zhebentyayeva et al. (2008) revealed that the *Plum Pox Virus* resistance occurring in North American germplasm also has a Chinese origin. Similar results were reported in peach, indicating that most modern cultivars in North America appeared to have originated from only a few old cultivars used in early twentieth-century North American breeding programs and coming from China (Cao et al., 2014).

## Conclusion

In conclusion, this large-scale analysis in apricot germplasm constituted a good example of the efficiency and high value of coordinated international actions to enhance the knowledge of worldwide variability. In fact, our study has provided a wider perspective on genetic diversity and structure within *P. armeniaca* L. species as well as the establishment of a core collection. Furthermore, from the center of origin, we have found evidence for three different diffusion routes, clarifying the worldwide scenario of domestication history for apricots. These results offer new opportunities for apricot breeding programs in the future related to (*i*) the maintenance of genetic diversity, (*ii*) defining strategies for efficient conservation of the genetic resources of this species, and (*iii*) improving quantitative traits using a genomic selection approach. Finally, the exploitation of the broad genetic diversity of apricot germplasm obtained will help genome-wide association studies linked to candidate genes in order to dissect complex traits as instigated by Mariette et al. (2016) for the resistance to *Plum Pox Virus* and/or the implementation of prospective multi-trait selection approaches for maximizing Value for Crop Use and Sustainability (VCUS) and minimizing the impact of climatic changes and biotic stress impacts.

## Data Availability Statement

The datasets generated for this study can be found in the public repositories. The full list of accessions and corresponding accession numbers can be found in Supplementary Table S1.

## Author Contributions

HB and J-MA conceived and designed the experiments. BK, TZ, CD’O, CL, HI, DC, LK, NT-F, WL, and J-MA provided the samples. GR and AR performed the experiments. HB, CS, IS, and J-MA analyzed the data. HB wrote the manuscript. J-MA, IS, and CS assisted in editing the manuscript. All authors read and approved the final manuscript.

## Conflict of Interest

The authors declare that the research was conducted in the absence of any commercial or financial relationships that could be construed as a potential conflict of interest.

## Supplementary Material

ADDITIONAL FILE S1 List of the 890 apricot accessions considered in the present study. Accession code, name, site of collection, geographical group of origin, cluster assignment inferred by the STRUCTURE analysis are reported. Accessions with asterisk are core collections members.

ADDITIONAL FILE S2 The parameters applied to DIYABC software.

ADDITIONAL FILE S3 Pairwise F_ST_ (above diagonal) and Nei’s genetic distances (below diagonal) values among the seven apricot geographic groups.

ADDITIONAL FILE S4 Neighbor-joining clustering of geographic groups based on pairwise Nei’s genetic distance values as well as the distribution of the five clusters within each of them. Color codes denotes the clusters identified by STRUCTURE analysis as reported in Figure 1, with Cluster 1 in red, Cluster 2 in blue, Cluster 3 in pink, Cluster 4 in yellow, and Cluster 5 in green. Numbers next to nodes indicate bootstrap support percentages in 1000 pseudoreplicates. Bootstrap values above 60% are shown.

ADDITIONAL FILE S5 Description of the four steps for the graphical method allowing determination of optimal *K* according to Evanno’s parameters.

ADDITIONAL FILE S6 Membership of apricot accessions to the five clusters as determined by STRUCTURE analysis.

ADDITIONAL FILE S7 Number and proportion (in parentheses) of accessions from different geographic groups assigned to the five clusters identified by STRUCURE analysis.

ADDITIONAL FILE S8 Parameters and results for bottleneck analyses used to detect significant reductions in effective population size in apricot clusters.
