# Supplementary material for: Genetic Structure of a Worldwide Germplasm Collection of Prunus armeniaca L. Reveals Three Major Diffusion Routes for Varieties Coming From the Species’ Center of Origin
Source: Front Plant Sci. 2020 May 25;11:638. doi: 10.3389/fpls.2020.00638 (PMC7261834; doi:10.3389/fpls.2020.00638)
Supplement: ADDITIONAL FILE S1 — List of the 890 apricot accessions considered in the present study. Accession code, name, site of collection, geographical group of origin, cluster assignment inferred by the STRUCTURE analysis are reported. Accessions with asterisk are core collections members. [file Data_Sheet_1.zip › Additioanl File 2.docx]

Additional file 2. The parameters applied to DIYABC software.

**DIYABC methods**

Evolutionary inference was designed to infer the parameters associated to the phylogenetic tree as reconstructed by TreeMix, with possible recent demographic change (expansion or bottleneck) and past admixture. Notice that in the DIYABC formalism, the branch of the tree leading (with introgression) to clusters 2, 3, and 5 needs to be modeled as a sixth (unsampled) population.

The code for the writing of the tree is as follows:

N1 N2 N3 N4 N5 N6

0 sample 1

0 sample 2

0 sample 3

0 sample 4

0 sample 5

t1 varNe 1 N1a

t2 varNe 2 N2b

t3 varNe 3 N3c

t4 varNe 4 N4d

t5 varNe 5 N5e

t6 split 3 1 6 r1

t7 split 2 1 6 r2

t8 split 5 1 6 r3

t9 merge 6 4

t9 merge 6 1

and the priors of the parameters are as described in the following tables:

| Parameter | Function | Min | max |
| --- | --- | --- | --- |
| N1 | logUniform | 10 | 100000 |
| N2 | logUniform | 10 | 100000 |
| N3 | logUniform | 10 | 100000 |
| N4 | logUniform | 10 | 100000 |
| N5 | logUniform | 10 | 100000 |
| N1a | logUniform | 10 | 100000 |
| N2b | logUniform | 10 | 100000 |
| N3c | logUniform | 10 | 100000 |
| N4d | logUniform | 10 | 100000 |
| N5e | logUniform | 10 | 100000 |
| t1 | Uniform | 10 | 100000 |
| t2 | Uniform | 10 | 100000 |
| t3 | Uniform | 10 | 100000 |
| t4 | Uniform | 10 | 100000 |
| t5 | Uniform | 10 | 100000 |
| t6 | Uniform | 10 | 100000 |
| t7 | Uniform | 10 | 100000 |
| t8 | Uniform | 10 | 100000 |
| t9 | Uniform | 10 | 100000 |
| r1 | uniform | 0.001 | 0.999 |
| r2 | uniform | 0.001 | 0.999 |
| r3 | uniform | 0.001 | 0.999 |

| Parameter | function | min | max | mean | shape |
| --- | --- | --- | --- | --- | --- |
| mean mut rate | Unif | 1e-4 | 1e-3 | 0.0005 | 2 |
| ind loc mut rate | gamma | 1e-5 | 1e-2 | mean_mu | 2 |
| mean P | unif | 0.1 | 0.3 | 0.22 | 2 |
| mean SNI rate | logUnif | 1e-8 | 1e-5 | 1e-7 | 2 |
| ind loc SNI rate | gamma | 1e-9 | 1e-4 | meanSNI | 2 |

The following summary statistics were used for inference:

Mean allele size variance for all clusters

Mean Garza-Williamson's M for all clusters

*F*_ST_ for all pairs of clusters

Three millions of simulations were run, and 30,000 (1%) were retained to build the posterior distributions.

The PCA plots for the first four principal components shows the position of the empirical summary statistic relative to the simulated ones:

The observed summary statistics are well represented by the simulations.
