# Supplementary material for: Genetic Structure of a Worldwide Germplasm Collection of Prunus armeniaca L. Reveals Three Major Diffusion Routes for Varieties Coming From the Species’ Center of Origin
Source: Front Plant Sci. 2020 May 25;11:638. doi: 10.3389/fpls.2020.00638 (PMC7261834; doi:10.3389/fpls.2020.00638)
Supplement: ADDITIONAL FILE S1 — List of the 890 apricot accessions considered in the present study. Accession code, name, site of collection, geographical group of origin, cluster assignment inferred by the STRUCTURE analysis are reported. Accessions with asterisk are core collections members. [file Data_Sheet_1.zip › Additional File 1.docx]

Additional file 1. List of the 890 apricot accessions considered in the present study. Accession code, name, their site of collection, geographical group of origin, the cluster assignment inferred by STRUCTURE analysis are reported. Accessions with asterisk are core collections members.

| N° | Accession code | Accession Name | Site of collection | Accession repository | Geographic group of origin | Cluster |
| --- | --- | --- | --- | --- | --- | --- |
| 1 | 25872^*^ | Bansei Oumi | Japan | NARO Institute | Eastern Asia | 1 |
| 2 | 25882^*^ | Heiwa | Japan | NARO Institute | Eastern Asia | 1 |
| 3 | 25883^*^ | Hiroshima Koanzu | Japan | NARO Institute | Eastern Asia | 1 |
| 4 | 25884^*^ | Jin Kyou | Japan | NARO Institute | Eastern Asia | 2 |
| 5 | 25885 | Jin Zhou Da Xing | Japan | NARO Institute | Eastern Asia | 1 |
| 6 | 25886^*^ | Jinshirou | Japan | NARO Institute | Eastern Asia | 1 |
| 7 | 25887^*^ | Kinrei | Japan | NARO Institute | Eastern Asia | 1 |
| 8 | 25888^*^ | Koushiu Oumi | Japan | NARO Institute | Eastern Asia | 1 |
| 9 | 25890 | Niigata Oumi | Japan | NARO Institute | Eastern Asia | 1 |
| 10 | 25891^*^ | Ogasawara | Japan | NARO Institute | Eastern Asia | 1 |
| 11 | 25892^*^ | Okitama | Japan | NARO Institute | Eastern Asia | 1 |
| 12 | 25896^*^ | Satakemaru | Japan | NARO Institute | Eastern Asia | 1 |
| 13 | 25897 | Satsuki | Japan | NARO Institute | Eastern Asia | 1 |
| 14 | 25898^*^ | Shimizugo | Japan | NARO Institute | Eastern Asia | 1 |
| 15 | 25899 | Shinheiwa | Japan | NARO Institute | Eastern Asia | 1 |
| 16 | 25900^*^ | Shiromomo Kitajima | Japan | NARO Institute | Eastern Asia | 1 |
| 17 | 25901^*^ | Shiromomo Kondousanae | Japan | NARO Institute | Eastern Asia | 1 |
| 18 | 25902^*^ | Takano Manjiu | Japan | NARO Institute | Eastern Asia | 1 |
| 19 | 25904^*^ | Todoroki Manjiu | Japan | NARO Institute | Eastern Asia | 1 |
| 20 | 25905^*^ | Tokoumaru | Japan | NARO Institute | Eastern Asia | 1 |
| 21 | 25906^*^ | Toua | Japan | NARO Institute | Eastern Asia | 1 |
| 22 | 25908^*^ | Yamagata 3 | Japan | NARO Institute | Eastern Asia | 1 |
| 23 | 25912^*^ | Mikanmomo | Japan | NARO Institute | Eastern Asia | 1 |
| 24 | A2852 | Chinese | France | INRA CRB GAFL | Eastern Asia | 4 |
| 25 | B10^*^ | Čína | China | Mendel University | Eastern Asia | 2 |
| 26 | B2^*^ | Chuang Sin | China | Mendel University | Eastern Asia | 2 |
| 27 | B6^*^ | Moi-Chua-Sin | China | Mendel University | Eastern Asia | mixed |
| 28 | B9^*^ | In-Bej-Sin | China | Mendel University | Eastern Asia | mixed |
| 29 | C12^*^ | Ansu | China | Mendel University | Eastern Asia | 1 |
| 30 | D1^*^ | Šantungská | China | Mendel University | Eastern Asia | 2 |
| 31 | D2^*^ | Da-Yu-bada | China | Mendel University | Eastern Asia | 2 |
| 32 | D6 | Mai-Che-Sin | China | Mendel University | Eastern Asia | mixed |
| 33 | D7^*^ | Mai-Huang | China | Mendel University | Eastern Asia | 2 |
| 34 | E10^*^ | 14 B | China | Mendel University | Eastern Asia | 2 |
| 35 | E12^*^ | He Bao Xing | China | Mendel University | Eastern Asia | mixed |
| 36 | E2^*^ | Pastyrik | China | Mendel University | Eastern Asia | 2 |
| 37 | E7^*^ | Yin Xang Bai Xing | China | Mendel University | Eastern Asia | 2 |
| 38 | E8^*^ | 5 Liaoning | China | Mendel University | Eastern Asia | 2 |
| 39 | E9^*^ | 3 B | China | Mendel University | Eastern Asia | 2 |
| 40 | F1^*^ | Hong Yu | China | Mendel University | Eastern Asia | mixed |
| 41 | F2^*^ | Dan Xing Bian Xing | China | Mendel University | Eastern Asia | 2 |
| 42 | F3^*^ | Sha Jin Hong | China | Mendel University | Eastern Asia | 2 |
| 43 | F4^*^ | 3 Liaoning | China | Mendel University | Eastern Asia | 2 |
| 44 | F6^*^ | L Liaoning | China | Mendel University | Eastern Asia | 2 |
| 45 | F7 | Beixan Heda Huang | China | Mendel University | Eastern Asia | 2 |
| 46 | F8^*^ | Liaoning | China | Mendel University | Eastern Asia | 2 |
| 47 | G10^*^ | ČLR 1 | China | Mendel University | Eastern Asia | 2 |
| 48 | G2^*^ | ČLR Da Jie Xing | China | Mendel University | Eastern Asia | 2 |
| 49 | G4^*^ | ČLR 8 | China | Mendel University | Eastern Asia | 2 |
| 50 | G5^*^ | ČLR 4 | China | Mendel University | Eastern Asia | 2 |
| 51 | G6^*^ | ČLR 10 | China | Mendel University | Eastern Asia | 2 |
| 52 | H3^*^ | Chuan Zhi Hong | China | Mendel University | Eastern Asia | 2 |
| 53 | U1588^*^ | Kanko-bai | Japan | USDA ARS Parlier | Eastern Asia | 2 |
| 54 | U2311^*^ | 96077 | China | USDA ARS Parlier | Eastern Asia | 2 |
| 55 | U2346 | RRS-1A | New Zealand | USDA ARS Parlier | Eastern Asia | mixed |
| 56 | U2426^*^ | Shanlian | Taiwan | USDA ARS Parlier | Eastern Asia | 2 |
| 57 | JM1^*^ | Da-bei | China | Nikita Botanical Garden | Eastern Asia | mixed |
| 58 | JM2^*^ | Da-chuan-che N1 | China | Nikita Botanical Garden | Eastern Asia | 2 |
| 59 | JM3^*^ | Da-chuan-che N2 | China | Nikita Botanical Garden | Eastern Asia | 2 |
| 60 | JM4^*^ | Kitaiskii | China | Nikita Botanical Garden | Eastern Asia | 2 |
| 61 | JM5 | Lao-yech-lian | China | Nikita Botanical Garden | Eastern Asia | mixed |
| 62 | JM6 | ln-ben-sin | China | Nikita Botanical Garden | Eastern Asia | 2 |
| 63 | JM7 | Mai-che-sin | China | Nikita Botanical Garden | Eastern Asia | 4 |
| 64 | JM8^*^ | Mi-bada | China | Nikita Botanical Garden | Eastern Asia | mixed |
| 65 | JM9^*^ | Pui-sha-sin | China | Nikita Botanical Garden | Eastern Asia | 2 |
| 66 | JM10^*^ | Shantunski | China | Nikita Botanical Garden | Eastern Asia | 2 |
| 67 | JM11 | Yuan-sin | China | Nikita Botanical Garden | Eastern Asia | 2 |
| 68 | A11^*^ | Kečpšar | France | Mendel University | Central Asia | 2 |
| 69 | A1275^*^ | Oranzeno Krasnyj | France | INRA CRB GAFL | Central Asia | 2 |
| 70 | A1333^*^ | Mitzourinskii2 | France | INRA CRB GAFL | Central Asia | 2 |
| 71 | A1693^*^ | Nikitski | France | INRA CRB GAFL | Europe | mixed |
| 72 | A2350 | Molodoi | France | INRA CRB GAFL | Central Asia | 5 |
| 73 | A3075^*^ | G1 A2039 | France | INRA CRB GAFL | Central Asia | mixed |
| 74 | A4082^*^ | Ladah | France | INRA CRB GAFL | Central Asia | 2 |
| 75 | B3^*^ | Roxana | USA | Mendel University | Central Asia | mixed |
| 76 | D8^*^ | Zard | USA | Mendel University | Central Asia | 2 |
| 77 | D9^*^ | Oranževo Krasnyj | USA | Mendel University | Central Asia | 2 |
| 78 | It59 | Monti Ladak | Italy | Università di Pisa | Central Asia | 4 |
| 79 | Tk65^*^ | Zard | Turkey | Malatya University | Central Asia | 2 |
| 80 | U0341 | Oranzhevo Krassny | USA | USDA ARS Parlier | Central Asia | 2 |
| 81 | U1372^*^ | Khubani | USA | USDA ARS Parlier | Central Asia | 2 |
| 82 | U1374^*^ |  | USA | USDA ARS Parlier | Central Asia | 2 |
| 83 | U1377 | Kabuli | USA | USDA ARS Parlier | Central Asia | 5 |
| 84 | U1380 | Habiju | USA | USDA ARS Parlier | Central Asia | 2 |
| 85 | U1381 | Habiju | USA | USDA ARS Parlier | Central Asia | mixed |
| 86 | U1383 | Janjir | USA | USDA ARS Parlier | Central Asia | 2 |
| 87 | U1384 | Habiju | USA | USDA ARS Parlier | Central Asia | mixed |
| 88 | U1385^*^ | Mirmamudi I | USA | USDA ARS Parlier | Central Asia | 2 |
| 89 | U1386 | Khuban | USA | USDA ARS Parlier | Central Asia | mixed |
| 90 | U1393 | Gaqai Shikanda | USA | USDA ARS Parlier | Central Asia | 2 |
| 91 | U1394^*^ | Burum Gakas | USA | USDA ARS Parlier | Central Asia | 2 |
| 92 | U1395^*^ | Badam Gakas | USA | USDA ARS Parlier | Central Asia | 2 |
| 93 | U1396^*^ | Bulbil-e-shikanda | USA | USDA ARS Parlier | Central Asia | 2 |
| 94 | U1398^*^ | Kartashi | USA | USDA ARS Parlier | Central Asia | 2 |
| 95 | U1401^*^ | Quropo | USA | USDA ARS Parlier | Central Asia | 2 |
| 96 | U1402^*^ | Dail Mesh | USA | USDA ARS Parlier | Central Asia | mixed |
| 97 | U1403^*^ | Lakesh | USA | USDA ARS Parlier | Central Asia | 2 |
| 98 | U1404 | Dolato Ju | USA | USDA ARS Parlier | Central Asia | 2 |
| 99 | U1405^*^ | Dolato Ju | USA | USDA ARS Parlier | Central Asia | 2 |
| 100 | U1407^*^ | Garkmish | USA | USDA ARS Parlier | Central Asia | 2 |
| 101 | U1408^*^ | Nagarum Shikanda | USA | USDA ARS Parlier | Central Asia | 2 |
| 102 | U1409^*^ | Gagai Tinani | USA | USDA ARS Parlier | Central Asia | 2 |
| 103 | U1410^*^ | Nazarali Kutzay Tinani | USA | USDA ARS Parlier | Central Asia | 2 |
| 104 | U1411^*^ | Shai Kutzay Ju | USA | USDA ARS Parlier | Central Asia | 2 |
| 105 | U1414 | Arghoon | USA | USDA ARS Parlier | Central Asia | 2 |
| 106 | U1416^*^ | Khoshenda | USA | USDA ARS Parlier | Central Asia | 2 |
| 107 | U1419 | Almon | USA | USDA ARS Parlier | Central Asia | 2 |
| 108 | U1421 | Karfochuli | USA | USDA ARS Parlier | Central Asia | 2 |
| 109 | U1423^*^ | Korkum Chuli | USA | USDA ARS Parlier | Central Asia | 2 |
| 110 | U1426 | Kachachuli | USA | USDA ARS Parlier | Central Asia | 2 |
| 111 | U1427^*^ | Badamchuli | USA | USDA ARS Parlier | Central Asia | 2 |
| 112 | U1428^*^ | Chaksa | USA | USDA ARS Parlier | Central Asia | 2 |
| 113 | U1429^*^ | Hashimpa | USA | USDA ARS Parlier | Central Asia | 2 |
| 114 | U1430^*^ | Khakas | USA | USDA ARS Parlier | Central Asia | 2 |
| 115 | U1432^*^ | Habiju | USA | USDA ARS Parlier | Central Asia | 2 |
| 116 | U1433 | Shikanda | USA | USDA ARS Parlier | Central Asia | 2 |
| 117 | U1434^*^ |  | USA | USDA ARS Parlier | Central Asia | mixed |
| 118 | U1435 |  | USA | USDA ARS Parlier | Central Asia | 2 |
| 119 | U1436 | Soris | USA | USDA ARS Parlier | Central Asia | 2 |
| 120 | U1438 | Paiwand | USA | USDA ARS Parlier | Central Asia | 2 |
| 121 | U1439^*^ |  | USA | USDA ARS Parlier | Central Asia | 2 |
| 122 | U1795^*^ | Luchak #6 | USA | USDA ARS Parlier | Central Asia | 2 |
| 123 | U1799^*^ | USSR 90-04-01 | USA | USDA ARS Parlier | Central Asia | 2 |
| 124 | U1800 | Yhulag | USA | USDA ARS Parlier | Central Asia | 2 |
| 125 | U1801^*^ | Mahtoby | USA | USDA ARS Parlier | Central Asia | 2 |
| 126 | U1802^*^ |  | USA | USDA ARS Parlier | Central Asia | 2 |
| 127 | U1803^*^ | USSR 90-11-01 | USA | USDA ARS Parlier | Central Asia | 2 |
| 128 | U1805^*^ | USSR 90-13-03 | USA | USDA ARS Parlier | Central Asia | 2 |
| 129 | U1807^*^ |  | USA | USDA ARS Parlier | Central Asia | 2 |
| 130 | U1809^*^ |  | USA | USDA ARS Parlier | Central Asia | 2 |
| 131 | U1810^*^ | USSR 90-16-04 | USA | USDA ARS Parlier | Central Asia | 2 |
| 132 | U1811^*^ | USSR 90-16-05 | USA | USDA ARS Parlier | Central Asia | 2 |
| 133 | U1812^*^ | USSR 90-16-06 | USA | USDA ARS Parlier | Central Asia | 2 |
| 134 | U1900^*^ | USSR 90-15-04 | USA | USDA ARS Parlier | Central Asia | mixed |
| 135 | U1901^*^ | USSR 90-16-03 | USA | USDA ARS Parlier | Central Asia | 2 |
| 136 | U2085 | Afghanistan E43-12 | USA | USDA ARS Parlier | Central Asia | 3 |
| 137 | U2279^*^ | USSR-90-06-01 | USA | USDA ARS Parlier | Central Asia | 2 |
| 138 | U2286 | Ak Luchak | USA | USDA ARS Parlier | Central Asia | mixed |
| 139 | U2307^*^ |  | USA | USDA ARS Parlier | Central Asia | 2 |
| 140 | U2308^*^ |  | USA | USDA ARS Parlier | Central Asia | 2 |
| 141 | U2309^*^ |  | USA | USDA ARS Parlier | Central Asia | 2 |
| 142 | U2310^*^ |  | USA | USDA ARS Parlier | Central Asia | 2 |
| 143 | U2390^*^ | Arzamy 37 | USA | USDA ARS Parlier | Central Asia | mixed |
| 144 | U2391^*^ | N262 | USA | USDA ARS Parlier | Central Asia | 2 |
| 145 | U2393^*^ | N270 | USA | USDA ARS Parlier | Central Asia | 2 |
| 146 | U2394^*^ | 280 | USA | USDA ARS Parlier | Central Asia | mixed |
| 147 | U2395^*^ | 14336 | USA | USDA ARS Parlier | Central Asia | mixed |
| 148 | U2422 | Tza-Sin | USA | USDA ARS Parlier | Central Asia | mixed |
| 149 | U2423^*^ | Da-Dze-Sin | USA | USDA ARS Parlier | Central Asia | mixed |
| 150 | U2425 | Badjok 93 | USA | USDA ARS Parlier | Central Asia | 2 |
| 151 | U2550 | IC 20089 | USA | USDA ARS Parlier | Central Asia | mixed |
| 152 | U2553^*^ | Subhany | USA | USDA ARS Parlier | Central Asia | 3 |
| 153 | U2570 | Germab-5 | USA | USDA ARS Parlier | Central Asia | mixed |
| 154 | U2590 | Birleshik 2 | USA | USDA ARS Parlier | Central Asia | mixed |
| 155 | JM12 | Arzami | Ukraine | Nikita Botanical Garden | Central Asia | 2 |
| 156 | JM13^*^ | B-1-11 | Ukraine | Nikita Botanical Garden | Central Asia | mixed |
| 157 | JM14^*^ | B-4-5 | Ukraine | Nikita Botanical Garden | Central Asia | 2 |
| 158 | JM15^*^ | B-5-3 | Ukraine | Nikita Botanical Garden | Central Asia | mixed |
| 159 | JM16^*^ | Badami | Ukraine | Nikita Botanical Garden | Central Asia | 2 |
| 160 | JM17^*^ | Dionis | Ukraine | Nikita Botanical Garden | Central Asia/ Irano-Caucasian | mixed |
| 161 | JM18^*^ | Gulyunghi | Ukraine | Nikita Botanical Garden | Central Asia | mixed |
| 162 | JM19^*^ | Iskadari | Ukraine | Nikita Botanical Garden | Central Asia | 2 |
| 163 | JM20^*^ | Kandak-10 | Ukraine | Nikita Botanical Garden | Central Asia | mixed |
| 164 | JM21 | Kandak-12 | Ukraine | Nikita Botanical Garden | Central Asia | mixed |
| 165 | JM22^*^ | KB-12 | Ukraine | Nikita Botanical Garden | Central Asia | 2 |
| 166 | JM23^*^ | KB-9 | Ukraine | Nikita Botanical Garden | Central Asia | 2 |
| 167 | JM24^*^ | Khurmai | Ukraine | Nikita Botanical Garden | Central Asia | 2 |
| 168 | JM25^*^ | Khurmai Rannii | Ukraine | Nikita Botanical Garden | Central Asia | 2 |
| 169 | JM26^*^ | KK(2) N1 | Ukraine | Nikita Botanical Garden | Central Asia | mixed |
| 170 | JM27^*^ | Kok-pshar | Ukraine | Nikita Botanical Garden | Central Asia | 2 |
| 171 | JM28^*^ | Kolon Boboi | Ukraine | Nikita Botanical Garden | Central Asia | 2 |
| 172 | JM29^*^ | Krimskii Amur | Ukraine | Nikita Botanical Garden | Central Asia/ Europe | mixed |
| 173 | JM30^*^ | Kunduzi | Ukraine | Nikita Botanical Garden | Central Asia | 2 |
| 174 | JM31^*^ | Kzyl Khorezmskii | Ukraine | Nikita Botanical Garden | Central Asia | mixed |
| 175 | JM32 | Kzyl Khurmai Kannibadam | Ukraine | Nikita Botanical Garden | Central Asia | 2 |
| 176 | JM33^*^ | Kzyl Uryuk | Ukraine | Nikita Botanical Garden | Central Asia | 2 |
| 177 | JM34^*^ | Lunnik | Ukraine | Nikita Botanical Garden | Irano-Caucasian/ Europe | 2 |
| 178 | JM35 | Lyuichak Sumbarski | Ukraine | Nikita Botanical Garden | Central Asia | mixed |
| 179 | JM36^*^ | Maftobi | Ukraine | Nikita Botanical Garden | Central Asia | mixed |
| 180 | JM37 | Medunets Krimskii | Ukraine | Nikita Botanical Garden | Central Asia/ Europe | mixed |
| 181 | JM38^*^ | Mirsandzhali | Ukraine | Nikita Botanical Garden | Central Asia | mixed |
| 182 | JM39^*^ | Naryadnyi | Ukraine | Nikita Botanical Garden | Central Asia/ Europe | mixed |
| 183 | JM40^*^ | Naslazhdenije | Ukraine | Nikita Botanical Garden | Irano-Caucasian/ Europe | 2 |
| 184 | JM41^*^ | Nukul Citronnyi | Ukraine | Nikita Botanical Garden | Central Asia | mixed |
| 185 | JM42^*^ | Dzhungarskii 18/55 | Ukraine | Nikita Botanical Garden | Central Asia | mixed |
| 186 | JM43^*^ | Dzhungarskii 18/63 | Ukraine | Nikita Botanical Garden | Central Asia | 2 |
| 187 | JM44 | Dzhungarskii 18/64 | Ukraine | Nikita Botanical Garden | Central Asia | 2 |
| 188 | JM45^*^ | Dzhungarskii 18/68 | Ukraine | Nikita Botanical Garden | Central Asia | mixed |
| 189 | JM46 | Dzhungarskii 18/75 | Ukraine | Nikita Botanical Garden | Central Asia | mixed |
| 190 | JM47 | Dzhungarskii 18/78 | Ukraine | Nikita Botanical Garden | Central Asia | 2 |
| 191 | JM48^*^ | *P. ansu* | China | Nikita Botanical Garden | Central Asia | 2 |
| 192 | JM49^*^ | *P. brigantina* | Alpine plum (outgroup) | Nikita Botanical Garden | Central Asia | 1 |
| 193 | JM50^*^ | *P. brigantina* x *P. armeniaca* (Olimp) | Interspecific hybrid (outgroup) | Nikita Botanical Garden | Central Asia | 2 |
| 194 | JM51^*^ | *P. brigantina* x *P. cerasifera* (Pionerka) | Interspecific hybrid (outgroup) | Nikita Botanical Garden | Central Asia | 2 |
| 195 | JM52^*^ | *P. Davida* N 7 (peach) | *Prunus davidiana* (outgroup) | Nikita Botanical Garden | Central Asia | 2 |
| 196 | JM53^*^ | *P. Davida* N 8 (peach) | *Prunus davidiana* (outgroup) | Nikita Botanical Garden | Central Asia | mixed |
| 197 | JM54^*^ | *P. mume* N15 | China | Nikita Botanical Garden | Central Asia | 2 |
| 198 | JM55^*^ | *P. mume* N18 | China | Nikita Botanical Garden | Central Asia | 2 |
| 199 | JM56^*^ | *P. sibirica* var *davidiana* | Russia | Nikita Botanical Garden | Central Asia | 2 |
| 200 | JM57^*^ | Paivandi Bukharskii | Ukraine | Nikita Botanical Garden | Central Asia | mixed |
| 201 | JM58^*^ | Parnas | Ukraine | Nikita Botanical Garden | Irano-Caucasian/ Europe | 2 |
| 202 | JM59^*^ | Priusadebnyi Rannii | Ukraine | Nikita Botanical Garden | Central Asia/ Europe | mixed |
| 203 | JM60^*^ | Rukhi Dzhuvanon Meona | Ukraine | Nikita Botanical Garden | Central Asia | 2 |
| 204 | JM61^*^ | Rukhi Dzhuvanon Surkh | Ukraine | Nikita Botanical Garden | Central Asia | 2 |
| 205 | JM62^*^ | Samyi Rannii | Ukraine | Nikita Botanical Garden | Central Asia | 2 |
| 206 | JM63 | Satser | *P. armeniaca* x *P. sibireca* | Nikita Botanical Garden | Central Asia | mixed |
| 207 | JM64^*^ | Supkhani | Ukraine | Nikita Botanical Garden | Central Asia | 2 |
| 208 | JM65 | Tadzhabai | Ukraine | Nikita Botanical Garden | Central Asia | mixed |
| 209 | JM66^*^ | Zard | Ukraine | Nikita Botanical Garden | Central Asia | mixed |
| 210 | 25847^*^ | Kabaasi | France | INRA CRB GAFL | Irano-Caucasian | 3 |
| 211 | 25848 | Igdir | France | INRA CRB GAFL | Irano-Caucasian | 3 |
| 212 | 25849 | Mahmudun Erigi | France | INRA CRB GAFL | Irano-Caucasian | 3 |
| 213 | 25850 | Alyanak | France | INRA CRB GAFL | Irano-Caucasian | mixed |
| 214 | 25864 | Cologlu | Turkey | INRA CRB GAFL | Irano-Caucasian | 3 |
| 215 | 25865 | Hasanbey | Turkey | INRA CRB GAFL | Irano-Caucasian | 3 |
| 216 | 25875 | Choloulu | Japan | NARO Institute | Irano-Caucasian | 3 |
| 217 | 25879 | Hajihaliloulu | Japan | NARO Institute | Irano-Caucasian | 3 |
| 218 | 25881 | Hasanbay | Japan | NARO Institute | Irano-Caucasian | 3 |
| 219 | A1267 | Badami | France | INRA CRB GAFL | Central Asia | 2 |
| 220 | A1406 | Charhoud | France | INRA CRB GAFL | Irano-Caucasian | mixed |
| 221 | A1458 | Nassiri | France | INRA CRB GAFL | Irano-Caucasian | 5 |
| 222 | A1868^*^ | Ordurao | France | INRA CRB GAFL | Irano-Caucasian | mixed |
| 223 | A1871^*^ | Semis | France | INRA CRB GAFL | Irano-Caucasian | mixed |
| 224 | A2166 | Tokaloglu | France | INRA CRB GAFL | Irano-Caucasian | mixed |
| 225 | A2292 | Karakabey | France | INRA CRB GAFL | Irano-Caucasian | mixed |
| 226 | A2294 | Sam | France | INRA CRB GAFL | Irano-Caucasian | 5 |
| 227 | A2348 | Erevani | France | INRA CRB GAFL | Irano-Caucasian | mixed |
| 228 | A2609 | Dorosthe | France | INRA CRB GAFL | Irano-Caucasian | mixed |
| 229 | A2613^*^ | Kermanshah | France | INRA CRB GAFL | Irano-Caucasian | mixed |
| 230 | A3508^*^ | G1 2121 6 | France | INRA CRB GAFL | Irano-Caucasian | mixed |
| 231 | A3509 | G1 2121 7 | France | INRA CRB GAFL | Irano-Caucasian | mixed |
| 232 | A3512^*^ | G1 2122 1 | France | INRA CRB GAFL | Irano-Caucasian | mixed |
| 233 | A3515^*^ | G1 2122 5 | France | INRA CRB GAFL | Irano-Caucasian | mixed |
| 234 | A3516 | G1 2122 6 | France | INRA CRB GAFL | Irano-Caucasian | mixed |
| 235 | A3517^*^ | G1 2122 7 | France | INRA CRB GAFL | Irano-Caucasian | mixed |
| 236 | A3518^*^ | G1 2122 8 | France | INRA CRB GAFL | Irano-Caucasian | mixed |
| 237 | A3521 | G1 2122 11 | France | INRA CRB GAFL | Irano-Caucasian | 3 |
| 238 | A3522^*^ | G1 2124 3 | France | INRA CRB GAFL | Irano-Caucasian | mixed |
| 239 | A3523 | G1 2053 3 | France | INRA CRB GAFL | Irano-Caucasian | mixed |
| 240 | A578 | Moustakaoui | France | INRA CRB GAFL | Irano-Caucasian | 5 |
| 241 | B1^*^ | Abu Talibu | USA | Mendel University | Irano-Caucasian | mixed |
| 242 | E1^*^ | Lasgerdi Mashad | USA | Mendel University | Irano-Caucasian | 2 |
| 243 | Tk1 | Adilcevaz-5 | Turkey | Malatya University | Irano-Caucasian | 3 |
| 244 | Tk10^*^ | Mahmudun Erigi | Turkey | Malatya University | Irano-Caucasian | mixed |
| 245 | Tk11 | Sekerpare | Turkey | Malatya University | Irano-Caucasian | 3 |
| 246 | Tk12 | Soganci | Turkey | Malatya University | Irano-Caucasian | mixed |
| 247 | Tk13 | Tokaloglu-Erzincan | Turkey | Malatya University | Irano-Caucasian | mixed |
| 248 | Tk14 | Tokaloglu-Yalova | Turkey | Malatya University | Irano-Caucasian | 5 |
| 249 | Tk15^*^ | Inciaz Erigi | Turkey | Malatya University | Irano-Caucasian | mixed |
| 250 | Tk16^*^ | Kayisi Erigi | Turkey | Malatya University | Irano-Caucasian | 2 |
| 251 | Tk17 | Agerik | Turkey | Malatya University | Irano-Caucasian | 3 |
| 252 | Tk18 | Alata Yildizi | Turkey | Malatya University | Irano-Caucasian | mixed |
| 253 | Tk19^*^ | Alyanak | Turkey | Malatya University | Irano-Caucasian | mixed |
| 254 | Tk2 | Cataloglu | Turkey | Malatya University | Irano-Caucasian | 3 |
| 255 | Tk20^*^ | Aprikoz | Turkey | Malatya University | Irano-Caucasian | mixed |
| 256 | Tk21^*^ | Cagataybey | Turkey | Malatya University | Irano-Caucasian | mixed |
| 257 | Tk22^*^ | Dr. Asma | Turkey | Malatya University | Irano-Caucasian | mixed |
| 258 | Tk23^*^ | Guz Aprikozu | Turkey | Malatya University | Irano-Caucasian | mixed |
| 259 | Tk24 | Imrahor | Turkey | Malatya University | Irano-Caucasian | 3 |
| 260 | Tk25^*^ | Iri Bitirgen | Turkey | Malatya University | Irano-Caucasian | 3 |
| 261 | Tk26^*^ | Kamelya | Turkey | Malatya University | Irano-Caucasian | mixed |
| 262 | Tk27^*^ | Karacabey | Turkey | Malatya University | Irano-Caucasian | 3 |
| 263 | Tk28 | Levent | Turkey | Malatya University | Irano-Caucasian | mixed |
| 264 | Tk29 | Sakit-1 | Turkey | Malatya University | Irano-Caucasian | 3 |
| 265 | Tk3 | Cologlu | Turkey | Malatya University | Irano-Caucasian | 3 |
| 266 | Tk30 | Sakit-2 | Turkey | Malatya University | Irano-Caucasian | 3 |
| 267 | Tk31^*^ | Sakit-3 | Turkey | Malatya University | Irano-Caucasian | 3 |
| 268 | Tk32 | Sakit-4 | Turkey | Malatya University | Irano-Caucasian | 3 |
| 269 | Tk33 | Sakit-6 | Turkey | Malatya University | Irano-Caucasian | mixed |
| 270 | Tk34^*^ | Sakit-7 | Turkey | Malatya University | Irano-Caucasian | 3 |
| 271 | Tk35 | Sam | Turkey | Malatya University | Irano-Caucasian | 5 |
| 272 | Tk36 | Torunoglu | Turkey | Malatya University | Irano-Caucasian | 3 |
| 273 | Tk37^*^ | Turfanda Eskimalatya | Turkey | Malatya University | Irano-Caucasian | 3 |
| 274 | Tk38 | Ziraat Okulu | Turkey | Malatya University | Irano-Caucasian | mixed |
| 275 | Tk39^*^ | Ordubat | Turkey | Malatya University | Irano-Caucasian | mixed |
| 276 | Tk5 | Hacihaliloglu | Turkey | Malatya University | Irano-Caucasian | 3 |
| 277 | Tk6 | Hacikiz | Turkey | Malatya University | Irano-Caucasian | mixed |
| 278 | Tk7 | Hasanbey | Turkey | Malatya University | Irano-Caucasian | 3 |
| 279 | Tk8 | Kabaasi | Turkey | Malatya University | Irano-Caucasian | 3 |
| 280 | Tk9^*^ | Kurukabuk | Turkey | Malatya University | Irano-Caucasian | mixed |
| 281 | U1787 |  | USA | USDA ARS Parlier | Irano-Caucasian | mixed |
| 282 | U1788 |  | USA | USDA ARS Parlier | Irano-Caucasian | mixed |
| 283 | U1847^*^ |  | USA | USDA ARS Parlier | Irano-Caucasian | 2 |
| 284 | U2065 | Lasgerdi Mashaad | USA | USDA ARS Parlier | Irano-Caucasian | 3 |
| 285 | U2095 | Turkish White | USA | USDA ARS Parlier | Irano-Caucasian | mixed |
| 286 | JM67 | Daradzhi ek Shabistr | Ukraine | Nikita Botanical Garden | Irano-Caucasian | 4 |
| 287 | JM68 | Katuni | Ukraine | Nikita Botanical Garden | Irano-Caucasian | 5 |
| 288 | JM69^*^ | Kurbane Marache | Ukraine | Nikita Botanical Garden | Irano-Caucasian | mixed |
| 289 | JM70^*^ | Mascat | Ukraine | Nikita Botanical Garden | Irano-Caucasian | 3 |
| 290 | JM71^*^ | Nakhichevanskii | Ukraine | Nikita Botanical Garden | Irano-Caucasian | mixed |
| 291 | JM72 | Nasera Tabris | Ukraine | Nikita Botanical Garden | Irano-Caucasian | 3 |
| 292 | JM73^*^ | Ordubad | Ukraine | Nikita Botanical Garden | Irano-Caucasian | 2 |
| 293 | JM74 | Shalakh | Ukraine | Nikita Botanical Garden | Irano-Caucasian | mixed |
| 294 | JM75 | Shekarpara de Semnan | Ukraine | Nikita Botanical Garden | Irano-Caucasian | 3 |
| 295 | JM76 | Vaagas Vardaguin | Ukraine | Nikita Botanical Garden | Irano-Caucasian | mixed |
| 296 | 3682 | Luizet | Switzerland | Agroscope | Continental Europe | 4 |
| 297 | 3684 | Luizet | Switzerland | Agroscope | Continental Europe | 4 |
| 298 | 3688 | Luizet | Switzerland | Agroscope | Continental Europe | 4 |
| 299 | 3703 | Luizet | Switzerland | Agroscope | Continental Europe | 4 |
| 300 | 3707 | Luizet | Switzerland | Agroscope | Continental Europe | 4 |
| 301 | 3708 | Luizet | Switzerland | Agroscope | Continental Europe | 4 |
| 302 | 3711 | Luizet | Switzerland | Agroscope | Continental Europe | 4 |
| 303 | 3712 | Luizet | Switzerland | Agroscope | Continental Europe | 4 |
| 304 | 3715 | Luizet | Switzerland | Agroscope | Continental Europe | 4 |
| 305 | 3718 | Luizet | Switzerland | Agroscope | Continental Europe | 4 |
| 306 | 3830^*^ | Luizet | Switzerland | Agroscope | Continental Europe | 4 |
| 307 | 3939 | Luizet | Switzerland | Agroscope | Continental Europe | 4 |
| 308 | 3941 | Luizet | Switzerland | Agroscope | Continental Europe | 4 |
| 309 | 3943 | Luizet | Switzerland | Agroscope | Continental Europe | 4 |
| 310 | 3944 | Luizet | Switzerland | Agroscope | Continental Europe | 4 |
| 311 | 3947 | Luizet | Switzerland | Agroscope | Continental Europe | 4 |
| 312 | 3948 | Luizet | Switzerland | Agroscope | Continental Europe | 4 |
| 313 | 3949 | Perrier | Switzerland | Agroscope | Continental Europe | 4 |
| 314 | 3951 | Luizet | Switzerland | Agroscope | Continental Europe | 4 |
| 315 | 25866 | Velcopavlovitsca | Turkey | INRA CRB GAFL | Continental Europe | mixed |
| 316 | 3685a | Luizet | Switzerland | Agroscope | Continental Europe | 4 |
| 317 | 3686a | Luizet | Switzerland | Agroscope | Continental Europe | 4 |
| 318 | 3689a | Luizet | Switzerland | Agroscope | Continental Europe | 4 |
| 319 | 3690b | Luizet | Switzerland | Agroscope | Continental Europe | 4 |
| 320 | 3691a | Luizet | Switzerland | Agroscope | Continental Europe | 4 |
| 321 | 3692a | Luizet | Switzerland | Agroscope | Continental Europe | 4 |
| 322 | 3695a | Luizet | Switzerland | Agroscope | Continental Europe | 4 |
| 323 | 3696a | Luizet | Switzerland | Agroscope | Continental Europe | 4 |
| 324 | 3699a | Luizet | Switzerland | Agroscope | Continental Europe | 4 |
| 325 | 3701a | Luizet | Switzerland | Agroscope | Continental Europe | 4 |
| 326 | 3702a | Luizet | Switzerland | Agroscope | Continental Europe | 4 |
| 327 | 3704a^*^ | Luizet | Switzerland | Agroscope | Continental Europe | 4 |
| 328 | 3942a | Luizet | Switzerland | Agroscope | Continental Europe | 4 |
| 329 | 3945a | Luizet | Switzerland | Agroscope | Continental Europe | 4 |
| 330 | 3950a | Luizet | Switzerland | Agroscope | Continental Europe | 4 |
| 331 | 3954a | Luizet | Switzerland | Agroscope | Continental Europe | 4 |
| 332 | 3955a | Luizet | Switzerland | Agroscope | Continental Europe | 4 |
| 333 | 3961a | Luizet | Switzerland | Agroscope | Continental Europe | 4 |
| 334 | 3966a | Luizet | Switzerland | Agroscope | Continental Europe | mixed |
| 335 | 3971a | Luizet | Switzerland | Agroscope | Continental Europe | 4 |
| 336 | 3972a | Luizet | Switzerland | Agroscope | Continental Europe | 4 |
| 337 | 7319a | Luizet | Switzerland | Agroscope | Continental Europe | 4 |
| 338 | A0144^*^ | Kesoi Rozsa | France | INRA CRB GAFL | Continental Europe | mixed |
| 339 | A12^*^ | Klosterneiburger | France | INRA CRB GAFL | Continental Europe | mixed |
| 340 | A1345^*^ | Cinska USL n°2 | France | INRA CRB GAFL | Continental Europe | 2 |
| 341 | A1663 | Cais Trandafiniu | France | INRA CRB GAFL | Continental Europe | mixed |
| 342 | A1690 | Uriase de Pecs | France | INRA CRB GAFL | Continental Europe | 4 |
| 343 | A1700 | Timpuri de Arad | France | INRA CRB GAFL | Continental Europe | 5 |
| 344 | A1800^*^ | Dietrich A1 | France | INRA CRB GAFL | Continental Europe | mixed |
| 345 | A2036 | F1 Abricotier x Amandier | Interspecific hybrid *P. armenica* x *P. dulcis* (outgroup) | INRA CRB GAFL | Continental Europe | mixed |
| 346 | A2147 | Stean Rosie | France | INRA CRB GAFL | Continental Europe | mixed |
| 347 | A2339 | De Hollanda | France | INRA CRB GAFL | Continental Europe | 5 |
| 348 | A2346^*^ | Timpurii de Kisinau | France | INRA CRB GAFL | Continental Europe | mixed |
| 349 | A2635 | Moorpark | France | INRA CRB GAFL | Continental Europe | 5 |
| 350 | A2655 | Karlotten | France | INRA CRB GAFL | Continental Europe | mixed |
| 351 | A2809 | Krupna Skopjanka | France | INRA CRB GAFL | Continental Europe | mixed |
| 352 | A3024^*^ | Kec Psar | France | INRA CRB GAFL | Central Asia | 2 |
| 353 | A601 | Beaugé | France | INRA CRB GAFL | Continental Europe | 5 |
| 354 | A7^*^ | Kostinskij | France | INRA CRB GAFL | Irano-Caucasian/ Europe | mixed |
| 355 | A755 | Rakovsheho | France | INRA CRB GAFL | Continental Europe | mixed |
| 356 | A882 | Madarska Narjlepsia | France | INRA CRB GAFL | Continental Europe | 4 |
| 357 | C11^*^ | Keczke Mete Rosen (Kecskemeti Rozsa) | Czech republic | Mendel University | Continental Europe | mixed |
| 358 | C6 | Krupnoplodá | Czech republic | Mendel University | Continental Europe | mixed |
| 359 | C9 | Jubilejnyj | Czech republic | Mendel University | Continental Europe | mixed |
| 360 | D11 | Chersonskij | Czech republic | Mendel University | Continental Europe | mixed |
| 361 | D3^*^ | Julskij | Czech republic | Mendel University | Continental Europe | 2 |
| 362 | MoranD2 | MoranD2 | Switzerland | Agroscope | Continental Europe | 4 |
| 363 | MorN | Morand N | Switzerland | Agroscope | Continental Europe | 4 |
| 364 | Tk47 | Hungarian Best | Turkey | Malatya University | Continental Europe | 4 |
| 365 | Tk61^*^ | Silistre de Rona | Turkey | Malatya University | Continental Europe | mixed |
| 366 | U1611 | Cegled De Mamut (cegledi mamut) | USA | USDA ARS Parlier | Continental Europe | mixed |
| 367 | U1638 | V/17 | USA | USDA ARS Parlier | Continental Europe | mixed |
| 368 | U2202 | UK-1 | USA | USDA ARS Parlier | Continental Europe | mixed |
| 369 | U2345 | Rakovsky BU 33 | USA | USDA ARS Parlier | Continental Europe | mixed |
| 370 | U2359 | Cacak's gold | USA | USDA ARS Parlier | Continental Europe | mixed |
| 371 | JM77 | Alberge de Tur | Ukraine | Nikita Botanical Garden | Continental Europe | 4 |
| 372 | JM78^*^ | Bergeron | Ukraine | Nikita Botanical Garden | Continental Europe | mixed |
| 373 | JM79^*^ | De Compot | Ukraine | Nikita Botanical Garden | Continental Europe | mixed |
| 374 | JM80 | Jubileinyi | Ukraine | Nikita Botanical Garden | Continental Europe | 4 |
| 375 | JM81 | Krasnoshchekii | Ukraine | Nikita Botanical Garden | Continental Europe | 4 |
| 376 | JM82 | Luizet Krupnoplodnyi | Ukraine | Nikita Botanical Garden | Continental Europe | mixed |
| 377 | JM83 | Precoce d'ltalia | Ukraine | Nikita Botanical Garden | Continental Europe | 5 |
| 378 | JM84 | Real d'Imola | Ukraine | Nikita Botanical Garden | Continental Europe | 5 |
| 379 | JM85 | Velkopavlovichka | Ukraine | Nikita Botanical Garden | Continental Europe | 5 |
| 380 | JM86 | Vengerskii Krypnyi | Ukraine | Nikita Botanical Garden | Continental Europe | mixed |
| 381 | JM87^*^ | Vynoslivyi | Ukraine | Nikita Botanical Garden | Continental Europe | 2 |
| 382 | 25826 | Currot | France | INRA CRB GAFL | Mediterranean Europe | mixed |
| 383 | 25827^*^ | Saint-Jean de Bruel | France | INRA CRB GAFL | Mediterranean Europe | mixed |
| 384 | 25828 | Précoce de Boulbon | France | INRA CRB GAFL | Mediterranean Europe | mixed |
| 385 | 25829 | Gros Rouge | France | INRA CRB GAFL | Mediterranean Europe | mixed |
| 386 | 25830 | Fleurit Tard | France | INRA CRB GAFL | Mediterranean Europe | 5 |
| 387 | 25831 | Royal | France | INRA CRB GAFL | Mediterranean Europe | mixed |
| 388 | 25832 | Poizat | France | INRA CRB GAFL | Mediterranean Europe | mixed |
| 389 | 25833^*^ | Merveilles du Dauphiné | France | INRA CRB GAFL | Mediterranean Europe | mixed |
| 390 | 25834 | Julin | France | INRA CRB GAFL | Mediterranean Europe | 4 |
| 391 | 25835 | Ampuis | France | INRA CRB GAFL | Mediterranean Europe | 5 |
| 392 | 25837 | Pêche de Nancy | France | INRA CRB GAFL | Mediterranean Europe | mixed |
| 393 | 25838^*^ | Rouge de Mauves | France | INRA CRB GAFL | Mediterranean Europe | mixed |
| 394 | 25839 | Muscat | France | INRA CRB GAFL | Mediterranean Europe | 5 |
| 395 | 25851 | Manri | Spain | Valencia | Mediterranean Europe | 5 |
| 396 | 25852 | Corbato | Spain | Valencia | Mediterranean Europe | 5 |
| 397 | 25853 | Rojo de Carlet | Spain | Valencia | Mediterranean Europe | 5 |
| 398 | 25854 | Palabras | Spain | Valencia | Mediterranean Europe | 5 |
| 399 | 25855 | Palau | Spain | Valencia | Mediterranean Europe | 5 |
| 400 | 25857 | Ginesta | Spain | Valencia | Mediterranean Europe | 5 |
| 401 | 25858 | Martinet | Spain | Valencia | Mediterranean Europe | 5 |
| 402 | 25859 | Cristali | Spain | Valencia | Mediterranean Europe | 5 |
| 403 | 25860 | Chirivello | Spain | Valencia | Mediterranean Europe | 5 |
| 404 | 25861 | Gandia | Spain | Valencia | Mediterranean Europe | 5 |
| 405 | 25862 | Gabachet | Spain | Valencia | Mediterranean Europe | 5 |
| 406 | 25863 | Currot | Spain | Valencia | Mediterranean Europe | 5 |
| 407 | 25869 | Angliers | France | INRA CRB GAFL | Mediterranean Europe | 5 |
| 408 | 25870 | Abricot plat | France | INRA CRB GAFL | Mediterranean Europe | mixed |
| 409 | 12PB390 | Monaco Bello | Italy | Università di Pisa | Mediterranean Europe | 5 |
| 410 | 12PB391 | Mandorla Dolce | Italy | Università di Pisa | Mediterranean Europe | 4 |
| 411 | 12PB392 | Precoce d'Imola | Italy | Università di Pisa | Mediterranean Europe | mixed |
| 412 | 12PB393^*^ | Rapareddu | Italy | Università di Pisa | Mediterranean Europe | 1 |
| 413 | 12PB394 | San Castrese | Italy | Università di Pisa | Mediterranean Europe | mixed |
| 414 | 12PB395 | Boccacia spinosa | Italy | Università di Pisa | Mediterranean Europe | 5 |
| 415 | 12PB396^*^ | Menace | Italy | Università di Pisa | Mediterranean Europe | 3 |
| 416 | 12PB417 | Dwarf Darnaud St Gervais | France | INRA CRB GAFL | Mediterranean Europe | mixed |
| 417 | 12PB592 | Ubones | Spain | Zaragoza | Mediterranean Europe | mixed |
| 418 | 12PB593 | Pepito grio | Spain | Zaragoza | Mediterranean Europe | 5 |
| 419 | 12PB594 | Juliette | Spain | Zaragoza | Mediterranean Europe | 5 |
| 420 | 12PB595 | Apr 25 PO | France | INRA CRB GAFL | Mediterranean Europe | 5 |
| 421 | 12PB596 | Apr 26 PO | France | INRA CRB GAFL | Mediterranean Europe | 5 |
| 422 | 12PB599 | Var Luventons | Spain | Mallorca | Mediterranean Europe | 5 |
| 423 | 12PB600 | Morro de Bou | Spain | Mallorca | Mediterranean Europe | mixed |
| 424 | 12PB601 | Bord Capona | Spain | Mallorca | Mediterranean Europe | 5 |
| 425 | 12PB602 | Del Marge | Spain | Mallorca | Mediterranean Europe | mixed |
| 426 | 12PB603 | Domas Blanc | Spain | Mallorca | Mediterranean Europe | mixed |
| 427 | 12PB604 | Moscatell | Spain | Mallorca | Mediterranean Europe | mixed |
| 428 | 12PB605 | Primarenc 4 | Spain | Mallorca | Mediterranean Europe | 5 |
| 429 | 12PB606 | Galta 1 | Spain | Mallorca | Mediterranean Europe | 3 |
| 430 | 12PB607 | Galta 2 | Spain | Mallorca | Mediterranean Europe | 3 |
| 431 | 12PB608 | Galta 4 | Spain | Mallorca | Mediterranean Europe | mixed |
| 432 | 12PB609 | Galta 5 | Spain | Mallorca | Mediterranean Europe | 3 |
| 433 | 12PB610 | Primarenc 1 | Spain | Mallorca | Mediterranean Europe | mixed |
| 434 | 12PB611^*^ | Taronsal | Spain | Mallorca | Mediterranean Europe | mixed |
| 435 | 12PB612 | Primarenc 3 | Spain | Mallorca | Mediterranean Europe | mixed |
| 436 | 12PB613 | Murto 2 | Spain | Mallorca | Mediterranean Europe | 5 |
| 437 | 12PB614 | Primarenc 2 | Spain | Mallorca | Mediterranean Europe | 5 |
| 438 | 12PB615 | Galta 5 (frano) | Spain | Mallorca | Mediterranean Europe | 3 |
| 439 | 12PB616 | Inquero | Spain | Mallorca | Mediterranean Europe | 5 |
| 440 | 12PB617 | Galta 8 (R Brot curt) | Spain | Mallorca | Mediterranean Europe | 3 |
| 441 | 12PB619 | Murto | Spain | Mallorca | Mediterranean Europe | 5 |
| 442 | 12PB620 | Galta 6 (Brot curt) | Spain | Mallorca | Mediterranean Europe | mixed |
| 443 | 12PB621 | Galta (Brot llarg) | Spain | Mallorca | Mediterranean Europe | mixed |
| 444 | A0014 | Bulida | France | INRA CRB GAFL | Mediterranean Europe | 4 |
| 445 | A0039^*^ | Ampuis | France | INRA CRB GAFL | Mediterranean Europe | mixed |
| 446 | A0076 | Pêche de Nancy | France | INRA CRB GAFL | Mediterranean Europe | mixed |
| 447 | A0082 | Hatif colomer | France | INRA CRB GAFL | Mediterranean Europe | 1 |
| 448 | A0110 | kaiska | France | INRA CRB GAFL | Mediterranean Europe | 5 |
| 449 | A0217 | Desfargue n°1 | France | INRA CRB GAFL | Mediterranean Europe | mixed |
| 450 | A0384 | A384 voisin colomer | France | INRA CRB GAFL | Mediterranean Europe | 5 |
| 451 | A0545 | Blanc rosé | France | INRA CRB GAFL | Mediterranean Europe | mixed |
| 452 | A0571 | proche Bergeron | France | INRA CRB GAFL | Mediterranean Europe | mixed |
| 453 | A0654 | Paviot | France | INRA CRB GAFL | Mediterranean Europe | 5 |
| 454 | A0657 | Poizat 20 | France | INRA CRB GAFL | Mediterranean Europe | mixed |
| 455 | A0924 | Canino | France | INRA CRB GAFL | Mediterranean Europe | mixed |
| 456 | A0010 | Sucré de Holub | France | INRA CRB GAFL | Mediterranean Europe | 5 |
| 457 | A1127 | Gabriel Bergeron | France | INRA CRB GAFL | Mediterranean Europe | mixed |
| 458 | A0114 | Bergeron | France | INRA CRB GAFL | Mediterranean Europe | mixed |
| 459 | A1176 | Alberchigo de confitar | France | INRA CRB GAFL | Mediterranean Europe | mixed |
| 460 | A1236 | Manicot | France | INRA CRB GAFL | Mediterranean Europe | mixed |
| 461 | A1236 | Manicot pied-mere | France | INRA CRB GAFL | Mediterranean Europe | mixed |
| 462 | A1311 | Proche Perla | France | INRA CRB GAFL | Mediterranean Europe | 5 |
| 463 | A1314^*^ | Arrogante | France | INRA CRB GAFL | Mediterranean Europe | 2 |
| 464 | A1317 | Gitano | France | INRA CRB GAFL | Mediterranean Europe | 5 |
| 465 | A1319 | Pepito | France | INRA CRB GAFL | Mediterranean Europe | 5 |
| 466 | A1352 | Polonais | France | INRA CRB GAFL | Mediterranean Europe | 5 |
| 467 | A1354 | Polonais n°2- Polonais Rebeyrolle | France | INRA CRB GAFL | Mediterranean Europe | 5 |
| 468 | A1356^*^ | Palummella | France | INRA CRB GAFL | Mediterranean Europe | mixed |
| 469 | A0157 | Rouge du Roussillon | France | INRA CRB GAFL | Mediterranean Europe | 5 |
| 470 | A1596 | San Fernando Biecheler | France | INRA CRB GAFL | Mediterranean Europe | 5 |
| 471 | A1600 | Canino | France | INRA CRB GAFL | Mediterranean Europe | 1 |
| 472 | A1601 | Bulida | France | INRA CRB GAFL | Mediterranean Europe | 5 |
| 473 | A1602 | Jaubert Foulon | France | INRA CRB GAFL | Mediterranean Europe | mixed |
| 474 | A1666 | RDR 377 | France | INRA CRB GAFL | Mediterranean Europe | 5 |
| 475 | A1685 | Précoce de Boulbon | France | INRA CRB GAFL | Mediterranean Europe | mixed |
| 476 | A1686 | Dr Mascle | France | INRA CRB GAFL | Mediterranean Europe | 1 |
| 477 | A1710 | Super Rouge | France | INRA CRB GAFL | Mediterranean Europe | mixed |
| 478 | A1711 | Avikaline | France | INRA CRB GAFL | Mediterranean Europe | mixed |
| 479 | A1712 | Colomer Arbre2 | France | INRA CRB GAFL | Mediterranean Europe | 1 |
| 480 | A1714 | Rouge de Sernhac | France | INRA CRB GAFL | Mediterranean Europe | 5 |
| 481 | A1721 | Précoce de Tyrinthe | France | INRA CRB GAFL | Mediterranean Europe | 4 |
| 482 | A1726 | ABR 882 | France | INRA CRB GAFL | Mediterranean Europe | 1 |
| 483 | A1744 | Prete | France | INRA CRB GAFL | Mediterranean Europe | 5 |
| 484 | A1745 | Albi Precocci n°1 | France | INRA CRB GAFL | Mediterranean Europe | 5 |
| 485 | A1748 | Donato | France | INRA CRB GAFL | Mediterranean Europe | mixed |
| 486 | A1750 | Boccuccia Eboli | France | INRA CRB GAFL | Mediterranean Europe | 5 |
| 487 | A1760 | Tardif de Bordaneil 2 | France | INRA CRB GAFL | Mediterranean Europe | mixed |
| 488 | A1792 | Houcall | France | INRA CRB GAFL | Mediterranean Europe | 1 |
| 489 | A1793 | Tardif de Bordaneil 1 | France | INRA CRB GAFL | Mediterranean Europe | mixed |
| 490 | A1801 | SH50 | France | INRA CRB GAFL | Mediterranean Europe | mixed |
| 491 | A1802 | SH7 | France | INRA CRB GAFL | Mediterranean Europe | 5 |
| 492 | A1809 | Précoce de Tyrinthe | France | INRA CRB GAFL | Mediterranean Europe | mixed |
| 493 | A1915 | San Castrese | France | INRA CRB GAFL | Mediterranean Europe | 5 |
| 494 | A1916^*^ | Pazza | France | INRA CRB GAFL | Mediterranean Europe | mixed |
| 495 | A1925 | Portici 2 | France | INRA CRB GAFL | Mediterranean Europe | mixed |
| 496 | A1939^*^ | Fantasme | France | INRA CRB GAFL | Mediterranean Europe | mixed |
| 497 | A1940 | Susincocco | France | INRA CRB GAFL | Mediterranean Europe | 5 |
| 498 | A2087 | Bebeco n°15 | France | INRA CRB GAFL | Mediterranean Europe | 5 |
| 499 | A2088 | Bebeco n°16 | France | INRA CRB GAFL | Mediterranean Europe | mixed |
| 500 | A2089 | Bebeco | France | INRA CRB GAFL | Mediterranean Europe | 5 |
| 501 | A2090 | Bebeco n°18 | France | INRA CRB GAFL | Mediterranean Europe | 5 |
| 502 | A2108 | Nancy | France | INRA CRB GAFL | Mediterranean Europe | 5 |
| 503 | A2129 | Rouge de Fournes | France | INRA CRB GAFL | Mediterranean Europe | mixed |
| 504 | A2131 | Carrascal | France | INRA CRB GAFL | Mediterranean Europe | mixed |
| 505 | A2187 | Bebeco La2A | France | INRA CRB GAFL | Mediterranean Europe | 5 |
| 506 | A2204 | Bebeco | France | INRA CRB GAFL | Mediterranean Europe | 5 |
| 507 | A2313 | Précoce du Portugal | France | INRA CRB GAFL | Mediterranean Europe | 1 |
| 508 | A2314 | Docteur Mascle | France | INRA CRB GAFL | Mediterranean Europe | 1 |
| 509 | A2319 | Taddeo | France | INRA CRB GAFL | Mediterranean Europe | 5 |
| 510 | A2330 | Monaco Bello | France | INRA CRB GAFL | Mediterranean Europe | mixed |
| 511 | A2351 | Précoce Pugget | France | INRA CRB GAFL | Mediterranean Europe | 1 |
| 512 | A2352^*^ | Precoce Firenze | France | INRA CRB GAFL | Mediterranean Europe | mixed |
| 513 | A2353 | Amabile Vecchioni | France | INRA CRB GAFL | Mediterranean Europe | mixed |
| 514 | A2354^*^ | Sernsniy | France | INRA CRB GAFL | Mediterranean Europe | mixed |
| 515 | A2645 | Bruel | France | INRA CRB GAFL | Mediterranean Europe | 1 |
| 516 | A2653 | Portici | France | INRA CRB GAFL | Mediterranean Europe | 5 |
| 517 | A2654^*^ | Viceroy | France | INRA CRB GAFL | Mediterranean Europe | mixed |
| 518 | A2657 | Vitillo | France | INRA CRB GAFL | Mediterranean Europe | mixed |
| 519 | A2719 | Cruzman | France | INRA CRB GAFL | Mediterranean Europe | mixed |
| 520 | A2720 | Pancin | France | INRA CRB GAFL | Mediterranean Europe | mixed |
| 521 | A2922 | Bulida | France | INRA CRB GAFL | Mediterranean Europe | 5 |
| 522 | A3408 | Ajouc | France | INRA CRB GAFL | Mediterranean Europe | mixed |
| 523 | A0380^*^ | Rouge de Rivesaltes 2 | France | INRA CRB GAFL | Mediterranean Europe | mixed |
| 524 | A3837^*^ | Royal | France | INRA CRB GAFL | Mediterranean Europe | 5 |
| 525 | A0039 | Précoce Ampuis | France | INRA CRB GAFL | Mediterranean Europe | mixed |
| 526 | A0526 | Precoce d'Italie | France | INRA CRB GAFL | Mediterranean Europe | 5 |
| 527 | A0539 | Poizat | France | INRA CRB GAFL | Mediterranean Europe | 5 |
| 528 | A5403 | Ampuis | France | INRA CRB GAFL | Mediterranean Europe | 5 |
| 529 | A5405^*^ | Delpierre | France | INRA CRB GAFL | Mediterranean Europe | 1 |
| 530 | A5406 | Delpierre Précoce | France | INRA CRB GAFL | Mediterranean Europe | 1 |
| 531 | A5406 | Delpierre Précoce2 | France | INRA CRB GAFL | Mediterranean Europe | 5 |
| 532 | A5407 | Mathieu Paret | France | INRA CRB GAFL | Mediterranean Europe | 5 |
| 533 | A5408 | Merveille Delpierre | France | INRA CRB GAFL | Mediterranean Europe | mixed |
| 534 | A5409 | Muscat Pêches Nancy | France | INRA CRB GAFL | Mediterranean Europe | mixed |
| 535 | A5410 | Paviot | France | INRA CRB GAFL | Mediterranean Europe | 5 |
| 536 | A5412 | Poman Rosé | France | INRA CRB GAFL | Mediterranean Europe | 5 |
| 537 | A5414 | Précoce de Boulbon | France | INRA CRB GAFL | Mediterranean Europe | mixed |
| 538 | A5415 | Dr Mascle Prospection | France | INRA CRB GAFL | Mediterranean Europe | mixed |
| 539 | A5417 | Précoce Saint Jean de Bruel | France | INRA CRB GAFL | Mediterranean Europe | 5 |
| 540 | A5418 | Pointu de Roqueverre | France | INRA CRB GAFL | Mediterranean Europe | mixed |
| 541 | A5419 | Apr 25 | France | INRA CRB GAFL | Mediterranean Europe | 5 |
| 542 | A5420 | Apr 26 | France | INRA CRB GAFL | Mediterranean Europe | 5 |
| 543 | A0544 | Cafona | France | INRA CRB GAFL | Mediterranean Europe | 5 |
| 544 | A0547 | Nanassa | France | INRA CRB GAFL | Mediterranean Europe | mixed |
| 545 | A0660 | Bergeron | France | INRA CRB GAFL | Mediterranean Europe | mixed |
| 546 | A0664 | Royer | France | INRA CRB GAFL | Mediterranean Europe | mixed |
| 547 | A0665 | Luizet | France | INRA CRB GAFL | Mediterranean Europe | 4 |
| 548 | A0692^*^ | Mandorlon | France | INRA CRB GAFL | Mediterranean Europe | mixed |
| 549 | A0074^*^ | Jaubert Foulon | France | INRA CRB GAFL | Mediterranean Europe | 1 |
| 550 | A0008 | Colomer | France | INRA CRB GAFL | Mediterranean Europe | 1 |
| 551 | A0804 | Screara | France | INRA CRB GAFL | Mediterranean Europe | 5 |
| 552 | A0873 | Reale d' Imola | France | INRA CRB GAFL | Mediterranean Europe | 5 |
| 553 | Es1 | Realfino | Spain | CSIC CEBAS Murcia | Mediterranean Europe | 5 |
| 554 | Es2 | Mauricio | Spain | CSIC CEBAS Murcia | Mediterranean Europe | 5 |
| 555 | Es3 | Currot | Spain | CSIC CEBAS Murcia | Mediterranean Europe | 5 |
| 556 | Es4 | Bulida | Spain | CSIC CEBAS Murcia | Mediterranean Europe | 5 |
| 557 | Es5 | Velazquez | Spain | CSIC CEBAS Murcia | Mediterranean Europe | 5 |
| 558 | Es6 | Canino | Spain | CSIC CEBAS Murcia | Mediterranean Europe | 5 |
| 559 | Es7 | Moniqui | Spain | CSIC CEBAS Murcia | Mediterranean Europe | 5 |
| 560 | Es8 | Pepito del Rubio | Spain | CSIC CEBAS Murcia | Mediterranean Europe | 5 |
| 561 | It01 | Acqua del Serino | Italy | Università di Pisa | Mediterranean Europe | 5 |
| 562 | It02 | Alessandrino | Italy | Università di Pisa | Mediterranean Europe | 5 |
| 563 | It03 | Amabile Vecchioni | Italy | Università di Pisa | Mediterranean Europe | mixed |
| 564 | It04 | Baracca | Italy | Università di Pisa | Mediterranean Europe | mixed |
| 565 | It05 | Bella d'Imola | Italy | Università di Pisa | Mediterranean Europe | mixed |
| 566 | It06^*^ | Bella si San Giuliano | Italy | Università di Pisa | Mediterranean Europe | 5 |
| 567 | It07^*^ | Boccuccia | Italy | Università di Pisa | Mediterranean Europe | 1 |
| 568 | It08 | Boccuccia Liscia | Italy | Università di Pisa | Mediterranean Europe | 5 |
| 569 | It09 | Boccuccia Spinosa | Italy | Università di Pisa | Mediterranean Europe | 5 |
| 570 | It10 | Breda (di) | Italy | Università di Pisa | Mediterranean Europe | 4 |
| 571 | It11 | Cafona MIPAF | Italy | Università di Pisa | Mediterranean Europe | 5 |
| 572 | It12 | Caldesi 1 | Italy | Università di Pisa | Mediterranean Europe | mixed |
| 573 | It13 | Caldesi 2 | Italy | Università di Pisa | Mediterranean Europe | mixed |
| 574 | It14 | Ceccona | Italy | Università di Pisa | Mediterranean Europe | mixed |
| 575 | It15 | Certosa A 5 | Italy | Università di Pisa | Mediterranean Europe | mixed |
| 576 | It16 | Certosa A 8 | Italy | Università di Pisa | Mediterranean Europe | mixed |
| 577 | It17 | Certosa B 5 | Italy | Università di Pisa | Mediterranean Europe | mixed |
| 578 | It18 | Cibo del Paradiso | Italy | Università di Pisa | Mediterranean Europe | 5 |
| 579 | It19 | Comune | Italy | Università di Pisa | Mediterranean Europe | mixed |
| 580 | It20 | Corona Quartucciu | Italy | Università di Pisa | Mediterranean Europe | mixed |
| 581 | It21 | Mandorlon | Italy | Università di Pisa | Mediterranean Europe | mixed |
| 582 | It22^*^ | Costa Sciacca Ragana 2 | Italy | Università di Pisa | Mediterranean Europe | mixed |
| 583 | It23 | Crisommene | Italy | Università di Pisa | Mediterranean Europe | 5 |
| 584 | It24 | D' Alessandria | Italy | Università di Pisa | Mediterranean Europe | 5 |
| 585 | It25^*^ | Dasycarpa PI 1 / 1 | Italy | Università di Pisa | Mediterranean Europe | mixed |
| 586 | It26 | Grossa del Giardino | Italy | Università di Pisa | Mediterranean Europe | 5 |
| 587 | It27 | Maiolino di Menfi | Italy | Università di Pisa | Mediterranean Europe | 5 |
| 588 | It28 | Mandorla Dolce | Italy | Università di Pisa | Mediterranean Europe | 4 |
| 589 | It29^*^ | Menace | Italy | Università di Pisa | Mediterranean Europe | 3 |
| 590 | It30 | Monaco | Italy | Università di Pisa | Mediterranean Europe | 5 |
| 591 | It31 | Monaco bello | Italy | Università di Pisa | Mediterranean Europe | 5 |
| 592 | It32^*^ | Monti Ladak 16 | Italy | Università di Pisa | Mediterranean Europe | mixed |
| 593 | It33^*^ | Monti Ladak 17 | Italy | Università di Pisa | Mediterranean Europe | 2 |
| 594 | It34^*^ | O' Pazzo | Italy | Università di Pisa | Mediterranean Europe | 5 |
| 595 | It35^*^ | Orru Quartu | Italy | Università di Pisa | Mediterranean Europe | 5 |
| 596 | It36 | Nonno | Italy | Università di Pisa | Mediterranean Europe | 5 |
| 597 | It37 | Particolare | Italy | Università di Pisa | Mediterranean Europe | 5 |
| 598 | It38 | Pelese di Giovanniello | Italy | Università di Pisa | Mediterranean Europe | 5 |
| 599 | It39^*^ | Pelluzzella | Italy | Università di Pisa | Mediterranean Europe | 5 |
| 600 | It40 | Persicini piccola | Italy | Università di Pisa | Mediterranean Europe | mixed |
| 601 | It41 | Del Pittore | Italy | Università di Pisa | Mediterranean Europe | mixed |
| 602 | It42 | Portici | Italy | Università di Pisa | Mediterranean Europe | 5 |
| 603 | It43 | Portici 2 | Italy | Università di Pisa | Mediterranean Europe | 5 |
| 604 | It44 | Precoce d' Imola | Italy | Università di Pisa | Mediterranean Europe | mixed |
| 605 | It45^*^ | Precoce d'Italia | Italy | Università di Pisa | Mediterranean Europe | 3 |
| 606 | It46 | Precoce di Sicilia | Italy | Università di Pisa | Mediterranean Europe | mixed |
| 607 | It47^*^ | Rapareddo | Italy | Università di Pisa | Mediterranean Europe | 3 |
| 608 | It48 | Reale d' Imola | Italy | Università di Pisa | Mediterranean Europe | 5 |
| 609 | It49 | San Castrese | Italy | Università di Pisa | Mediterranean Europe | mixed |
| 610 | It50 | San Francesco | Italy | Università di Pisa | Mediterranean Europe | mixed |
| 611 | It51 | Sant' Ambrogio | Italy | Università di Pisa | Mediterranean Europe | 1 |
| 612 | It52 | Saritzu I | Italy | Università di Pisa | Mediterranean Europe | mixed |
| 613 | It53 | Saritzu II | Italy | Università di Pisa | Mediterranean Europe | mixed |
| 614 | It54 | Tardiva di Menfi | Italy | Università di Pisa | Mediterranean Europe | 5 |
| 615 | It55 | Tiltonno | Italy | Università di Pisa | Mediterranean Europe | mixed |
| 616 | It56^*^ | Ungherese gialla | Italy | Università di Pisa | Mediterranean Europe | 5 |
| 617 | It57 | Ungherese piccola | Italy | Università di Pisa | Mediterranean Europe | 5 |
| 618 | It58 | Valleggia | Italy | Università di Pisa | Mediterranean Europe | 5 |
| 619 | It60^*^ | Vitillo | Italy | Università di Pisa | Mediterranean Europe | mixed |
| 620 | Tk40 | Bebeco | Turkey | Malatya University | Mediterranean Europe | 5 |
| 621 | Tk42 | Canino | Turkey | Malatya University | Mediterranean Europe | mixed |
| 622 | Tk44 | Fracasso | Turkey | Malatya University | Mediterranean Europe | mixed |
| 623 | Tk51 | Paviot | Turkey | Malatya University | Mediterranean Europe | mixed |
| 624 | Tk53^*^ | Polenais | Turkey | Malatya University | Mediterranean Europe | mixed |
| 625 | Tk54 | Precoce de Boulbon | Turkey | Malatya University | Mediterranean Europe | 3 |
| 626 | Tk55 | Precoce de Thyrinte | Turkey | Malatya University | Mediterranean Europe | mixed |
| 627 | Tk56 | Precoce de Colomer | Turkey | Malatya University | Mediterranean Europe | 1 |
| 628 | Tk59 | Royal | Turkey | Malatya University | Mediterranean Europe | mixed |
| 629 | Tk60 | Sancastrese | Turkey | Malatya University | Mediterranean Europe | 1 |
| 630 | Tk63 | Vitillo | Turkey | Malatya University | Mediterranean Europe | mixed |
| 631 | U0945 | Alberge De Montgamet | USA | USDA ARS Parlier | Mediterranean Europe | 4 |
| 632 | 3 | Bou Herra | Tunisia | *in situ* sampling | North-Africa | mixed |
| 633 | 9 | Chéchi Dhraa Tammar | Tunisia | *in situ* sampling | North-Africa | 5 |
| 634 | 15 | Addadi Ahmar | Tunisia | *in situ* sampling | North-Africa | mixed |
| 635 | 16^*^ | Om Younes | Tunisia | *in situ* sampling | North-Africa | mixed |
| 636 | 27 | Oud Gnaa | Tunisia | *in situ* sampling | North-Africa | 3 |
| 637 | 29 | Chéchi Horr | Tunisia | *in situ* sampling | North-Africa | 5 |
| 638 | 31 | Bangui | Tunisia | *in situ* sampling | North-Africa | mixed |
| 639 | 47 | Variété de Mahdia | Tunisia | *in situ* sampling | North-Africa | 5 |
| 640 | 67^*^ | Jerba 67 | Tunisia | *in situ* sampling | North-Africa | mixed |
| 641 | 68 | Jerba 68 | Tunisia | *in situ* sampling | North-Africa | 3 |
| 642 | 69 | Jerba 69 | Tunisia | *in situ* sampling | North-Africa | 3 |
| 643 | 70 | Oud El Haj Tahar | Tunisia | *in situ* sampling | North-Africa | 3 |
| 644 | 71 | Oud Aouicha | Tunisia | *in situ* sampling | North-Africa | mixed |
| 645 | 72 | Zalouzi | Tunisia | *in situ* sampling | North-Africa | 3 |
| 646 | 25845 | Chechi | France | INRA CRB GAFL | North-Africa | 5 |
| 647 | 25846 | Bayoudhi | France | INRA CRB GAFL | North-Africa | 5 |
| 648 | 10A | Chéchi Khit El Oued | Tunisia | *in situ* sampling | North-Africa | 5 |
| 649 | 11B^*^ | Bayoudhi | Tunisia | *in situ* sampling | North-Africa | 5 |
| 650 | 12C | H'midi | Tunisia | *in situ* sampling | North-Africa | mixed |
| 651 | 13A | Bouk Hmed | Tunisia | *in situ* sampling | North-Africa | 3 |
| 652 | 14C | Faggoussi | Tunisia | *in situ* sampling | North-Africa | mixed |
| 653 | 17A | Aranji | Tunisia | *in situ* sampling | North-Africa | 3 |
| 654 | 18A | Oud Rhayem | Tunisia | *in situ* sampling | North-Africa | mixed |
| 655 | 19A | Bedri Ahmar | Tunisia | *in situ* sampling | North-Africa | mixed |
| 656 | 1C | Baccour | Tunisia | *in situ* sampling | North-Africa | 3 |
| 657 | 1G | Bedri | Tunisia | *in situ* sampling | North-Africa | mixed |
| 658 | 20C | Bouthani | Tunisia | *in situ* sampling | North-Africa | 3 |
| 659 | 21A | Oud Hmida | Tunisia | *in situ* sampling | North-Africa | 3 |
| 660 | 22B | Oud Tijani | Tunisia | *in situ* sampling | North-Africa | mixed |
| 661 | 23B | Oud Nakhla | Tunisia | *in situ* sampling | North-Africa | 3 |
| 662 | 25B | Oud Salah Ben Salem | Tunisia | *in situ* sampling | North-Africa | 3 |
| 663 | 28D | Chechi Bazza | Tunisia | *in situ* sampling | North-Africa | mixed |
| 664 | 2C | Khad Hlima | Tunisia | *in situ* sampling | North-Africa | 5 |
| 665 | 34B | Bouk Hmed Akhal | Tunisia | *in situ* sampling | North-Africa | 3 |
| 666 | 38B^*^ | Fourati | Tunisia | *in situ* sampling | North-Africa | 5 |
| 667 | 40A | Bargoug 40A | Tunisia | *in situ* sampling | North-Africa | 3 |
| 668 | 40B | Bargoug 40B | Tunisia | *in situ* sampling | North-Africa | 3 |
| 669 | 40E | Bargoug 40E | Tunisia | *in situ* sampling | North-Africa | 3 |
| 670 | 40G^*^ | Bargoug 40G | Tunisia | *in situ* sampling | North-Africa | 3 |
| 671 | 40H | Bargoug 40H | Tunisia | *in situ* sampling | North-Africa | 3 |
| 672 | 40I | Bargoug 40I | Tunisia | *in situ* sampling | North-Africa | 3 |
| 673 | 40J | Bargoug 40J | Tunisia | *in situ* sampling | North-Africa | 3 |
| 674 | 40K | Bargoug 40K | Tunisia | *in situ* sampling | North-Africa | 3 |
| 675 | 40M^*^ | Bargoug 40M | Tunisia | *in situ* sampling | North-Africa | 3 |
| 676 | 40N^*^ | Bargoug 40N | Tunisia | *in situ* sampling | North-Africa | mixed |
| 677 | 41A | Agrégé de Baccour | Tunisia | *in situ* sampling | North-Africa | 3 |
| 678 | 42A | Bargoug 42A | Tunisia | *in situ* sampling | North-Africa | 3 |
| 679 | 42B | Bargoug42B | Tunisia | *in situ* sampling | North-Africa | 3 |
| 680 | 42C^*^ | Bargoug 42C | Tunisia | *in situ* sampling | North-Africa | 3 |
| 681 | 42G | Bargoug 42G | Tunisia | *in situ* sampling | North-Africa | 3 |
| 682 | 42H^*^ | Bargoug 42H | Tunisia | *in situ* sampling | North-Africa | 3 |
| 683 | 43B^*^ | Bargoug 43B | Tunisia | *in situ* sampling | North-Africa | 3 |
| 684 | 43C | Bargoug 43C | Tunisia | *in situ* sampling | North-Africa | 3 |
| 685 | 43D | Bargoug 43D | Tunisia | *in situ* sampling | North-Africa | 3 |
| 686 | 43F | Bargoug 43F | Tunisia | *in situ* sampling | North-Africa | 3 |
| 687 | 44A | Bargoug 44A | Tunisia | *in situ* sampling | North-Africa | 3 |
| 688 | 44B | Bargoug 44B | Tunisia | *in situ* sampling | North-Africa | 3 |
| 689 | 44C | Bargoug 44C | Tunisia | *in situ* sampling | North-Africa | 3 |
| 690 | 44D^*^ | Bargoug 44D | Tunisia | *in situ* sampling | North-Africa | 3 |
| 691 | 44E | Bargoug 44E | Tunisia | *in situ* sampling | North-Africa | 3 |
| 692 | 44F | Bargoug 44F | Tunisia | *in situ* sampling | North-Africa | 3 |
| 693 | 44G | Bargoug 44G | Tunisia | *in situ* sampling | North-Africa | 3 |
| 694 | 44H | Bargoug 44H | Tunisia | *in situ* sampling | North-Africa | 3 |
| 695 | 45B^*^ | Bargoug 45B | Tunisia | *in situ* sampling | North-Africa | 3 |
| 696 | 45C | Bargoug 45C | Tunisia | *in situ* sampling | North-Africa | 3 |
| 697 | 46B | Bargoug 46B | Tunisia | *in situ* sampling | North-Africa | 3 |
| 698 | 46C^*^ | Bargoug 46C | Tunisia | *in situ* sampling | North-Africa | 3 |
| 699 | 46D | Bargoug 46D | Tunisia | *in situ* sampling | North-Africa | 3 |
| 700 | 46E | Bargoug 46E | Tunisia | *in situ* sampling | North-Africa | 3 |
| 701 | 48A | Bedri | Tunisia | *in situ* sampling | North-Africa | mixed |
| 702 | 48G | Bedri Thani | Tunisia | *in situ* sampling | North-Africa | mixed |
| 703 | 49A | Louzi Thani | Tunisia | *in situ* sampling | North-Africa | 3 |
| 704 | 4B | Najjar | Tunisia | *in situ* sampling | North-Africa | 3 |
| 705 | 53A | Ben Souileh | Tunisia | *in situ* sampling | North-Africa | 3 |
| 706 | 57A | Bedri Louzi | Tunisia | *in situ* sampling | North-Africa | mixed |
| 707 | 58B | Thani | Tunisia | *in situ* sampling | North-Africa | 3 |
| 708 | 59A | Theleth | Tunisia | *in situ* sampling | North-Africa | 3 |
| 709 | 5C | Amor El Euch | Tunisia | *in situ* sampling | North-Africa | 3 |
| 710 | 62A | Bou Khobza | Tunisia | *in situ* sampling | North-Africa | 3 |
| 711 | 65B | Mazouzi | Tunisia | *in situ* sampling | North-Africa | 3 |
| 712 | 6A | Messelmani | Tunisia | *in situ* sampling | North-Africa | 3 |
| 713 | 6B | Agrégé de Messelmani | Tunisia | *in situ* sampling | North-Africa | 3 |
| 714 | 7C | Zbidi | Tunisia | *in situ* sampling | North-Africa | 3 |
| 715 | A0795 | Canino | France | INRA CRB GAFL | North-Africa | 5 |
| 716 | A1592 | kasserine n°2 Bergeron | France | INRA CRB GAFL | North-Africa | mixed |
| 717 | A1594 | kasserine n°1 polonais | France | INRA CRB GAFL | North-Africa | 5 |
| 718 | A1598 | Hatif Colomer | France | INRA CRB GAFL | North-Africa | 1 |
| 719 | A2065 | Marouch 4 | France | INRA CRB GAFL | North-Africa | mixed |
| 720 | A2067 | Marouch 14 | France | INRA CRB GAFL | North-Africa | 5 |
| 721 | A2102^*^ | Marouch 16 | France | INRA CRB GAFL | North-Africa | 5 |
| 722 | A2137^*^ | BAKOUR | France | INRA CRB GAFL | North-Africa | 3 |
| 723 | Al.01 | Louzi Local | Algeria | *in situ* sampling | North-Africa | 4 |
| 724 | Al.02 | Boulila Rouge | Algeria | *in situ* sampling | North-Africa | 5 |
| 725 | Al.04 | Canino | Algeria | *in situ* sampling | North-Africa | 5 |
| 726 | Al.05 | Louzi Rouge | Algeria | *in situ* sampling | North-Africa | 5 |
| 727 | Al.06 | Louzi blanc | Algeria | *in situ* sampling | North-Africa | 5 |
| 728 | Al.07^*^ | Rosé de Corail | Algeria | *in situ* sampling | North-Africa | mixed |
| 729 | Al.08^*^ | Rosé de Ménaa | Algeria | *in situ* sampling | North-Africa | mixed |
| 730 | Al.09 | Paviot Rouge | Algeria | *in situ* sampling | North-Africa | mixed |
| 731 | Al.10^*^ | Paviot blanc | Algeria | *in situ* sampling | North-Africa | mixed |
| 732 | Al.11 | Boulida | Algeria | *in situ* sampling | North-Africa | 5 |
| 733 | Al.12 | Rouge du Roussillon | Algeria | *in situ* sampling | North-Africa | 1 |
| 734 | Al.13 | Pêcher | Algeria | *in situ* sampling | North-Africa | mixed |
| 735 | Al.14^*^ | Mechmech Hlou | Algeria | *in situ* sampling | North-Africa | mixed |
| 736 | Al.15 | Gros MechMech | Algeria | *in situ* sampling | North-Africa | 5 |
| 737 | Al.16 | Mechmech Laghdech | Algeria | *in situ* sampling | North-Africa | mixed |
| 738 | Al.17 | Nail | Algeria | *in situ* sampling | North-Africa | 3 |
| 739 | Al.18 | Abyad el Imlak | Algeria | *in situ* sampling | North-Africa | 3 |
| 740 | Al.19 | Laouzi greffé | Algeria | *in situ* sampling | North-Africa | mixed |
| 741 | Al.20 | Nda el Morjane | Algeria | *in situ* sampling | North-Africa | 3 |
| 742 | Al.21 | Pêcher Rouge | Algeria | *in situ* sampling | North-Africa | 3 |
| 743 | Al.22 | Arbi V1 | Algeria | *in situ* sampling | North-Africa | 3 |
| 744 | Al.23 | Arbia Nadir | Algeria | *in situ* sampling | North-Africa | 3 |
| 745 | Al.24 | Mahalat el Djoundi | Algeria | *in situ* sampling | North-Africa | 3 |
| 746 | Al.25 | Louzia greffé | Algeria | *in situ* sampling | North-Africa | 5 |
| 747 | Al.26 | Arbi Kd | Algeria | *in situ* sampling | North-Africa | 3 |
| 748 | Al.27 | Pêcher Blanc | Algeria | *in situ* sampling | North-Africa | 5 |
| 749 | Al.28 | Mouzemèche | Algeria | *in situ* sampling | North-Africa | mixed |
| 750 | Al.29 | Kahf | Algeria | *in situ* sampling | North-Africa | mixed |
| 751 | Al.30 | Messaad Greffé | Algeria | *in situ* sampling | North-Africa | mixed |
| 752 | Al.31 | Louizi Rouge | Algeria | *in situ* sampling | North-Africa | mixed |
| 753 | Al.32 | Arbi Nadir | Algeria | *in situ* sampling | North-Africa | 3 |
| 754 | Al.33 | Saafi Arbi | Algeria | *in situ* sampling | North-Africa | mixed |
| 755 | Al.34 | El Maghreb | Algeria | *in situ* sampling | North-Africa | 3 |
| 756 | Al.35 | Hamrai | Algeria | *in situ* sampling | North-Africa | 2 |
| 757 | Al.36 | Moutaakhir | Algeria | *in situ* sampling | North-Africa | mixed |
| 758 | Al.37 | Hmarai tardif | Algeria | *in situ* sampling | North-Africa | mixed |
| 759 | Al.38 | Pêcher blanc M | Algeria | *in situ* sampling | North-Africa | 5 |
| 760 | Al.39^*^ | El Bakria | Algeria | *in situ* sampling | North-Africa | 3 |
| 761 | Al.40 | Douk el Kamel | Algeria | *in situ* sampling | North-Africa | 3 |
| 762 | Al.41 | Bulida M | Algeria | *in situ* sampling | North-Africa | mixed |
| 763 | Al.42 | Mnadir greffé M | Algeria | *in situ* sampling | North-Africa | 5 |
| 764 | Al.43 | Hamrai Greffé | Algeria | *in situ* sampling | North-Africa | 3 |
| 765 | Al.44 | Percher sur franc | Algeria | *in situ* sampling | North-Africa | 5 |
| 766 | Al.45 | Chems el Massa | Algeria | *in situ* sampling | North-Africa | 3 |
| 767 | Al.46 | Naila | Algeria | *in situ* sampling | North-Africa | 3 |
| 768 | Al.47 | Saib Ennahdha | Algeria | *in situ* sampling | North-Africa | 3 |
| 769 | Al.48^*^ | Ikhtiyar ettayeb | Algeria | *in situ* sampling | North-Africa | 3 |
| 770 | K2 | Kasserine K2 | Tunisia | *in situ* sampling | North-Africa | mixed |
| 771 | L1V1 | Goulmima AJG1 | Morocco | INRAM Meknès | North-Africa | 3 |
| 772 | L1V10 | Kalaat Meggouna G1 | Morocco | INRAM Meknès | North-Africa | 3 |
| 773 | L1V11^*^ | Kalaat Meggouna G2 | Morocco | INRAM Meknès | North-Africa | mixed |
| 774 | L1V3^*^ | Jorf 7 | Morocco | INRAM Meknès | North-Africa | 3 |
| 775 | L1V4 | Jorf 8 | Morocco | INRAM Meknès | North-Africa | 3 |
| 776 | L1V5 | Goulmima AJG2 | Morocco | INRAM Meknès | North-Africa | 3 |
| 777 | L1V6^*^ | Boumalen J3 | Morocco | INRAM Meknès | North-Africa | 3 |
| 778 | L1V7 | Agdez A7 | Morocco | INRAM Meknès | North-Africa | 5 |
| 779 | L1V8 | Agdez A8 | Morocco | INRAM Meknès | North-Africa | 5 |
| 780 | L1V9 | Skoura SKH2 | Morocco | INRAM Meknès | North-Africa | 3 |
| 781 | L2V1^*^ | Rich RK1 | Morocco | INRAM Meknès | North-Africa | 5 |
| 782 | L2V3 | Rich 3 | Morocco | INRAM Meknès | North-Africa | 5 |
| 783 | L2V4^*^ | Rich 3 | Morocco | INRAM Meknès | North-Africa | 3 |
| 784 | L2V5 | Jorf 6 | Morocco | INRAM Meknès | North-Africa | 3 |
| 785 | L2V6^*^ | Kalaat Meggouna G6 | Morocco | INRAM Meknès | North-Africa | mixed |
| 786 | L2V8 | Boumalen A3 | Morocco | INRAM Meknès | North-Africa | 3 |
| 787 | L3V1 | Rich 4 | Morocco | INRAM Meknès | North-Africa | 3 |
| 788 | L3V11 | Agdez C1 | Morocco | INRAM Meknès | North-Africa | mixed |
| 789 | L3V2^*^ | Goulmima RG1 | Morocco | INRAM Meknès | North-Africa | 3 |
| 790 | L3V3^*^ | Rtil 1 | Morocco | INRAM Meknès | North-Africa | mixed |
| 791 | L3V4 | Rtil 2 | Morocco | INRAM Meknès | North-Africa | 4 |
| 792 | L3V5 | Can 1 | Morocco | INRAM Meknès | North-Africa | 5 |
| 793 | L3V7 | Kalaat Meggouna G5 | Morocco | INRAM Meknès | North-Africa | mixed |
| 794 | L3V8 | Skoura SKH3 | Morocco | INRAM Meknès | North-Africa | 3 |
| 795 | L3V9 | Boumalen A4 | Morocco | INRAM Meknès | North-Africa | 3 |
| 796 | L4V1 | Rtil 4 | Morocco | INRAM Meknès | North-Africa | mixed |
| 797 | L4V10 | Agdez A6 | Morocco | INRAM Meknès | North-Africa | 3 |
| 798 | L4V2 | Rtil 5 | Morocco | INRAM Meknès | North-Africa | 3 |
| 799 | L4V3 | Goulmima GR1 | Morocco | INRAM Meknès | North-Africa | 3 |
| 800 | L4V4^*^ | Goulmima GM1 | Morocco | INRAM Meknès | North-Africa | 3 |
| 801 | L4V5 | Geli | Morocco | INRAM Meknès | North-Africa | 5 |
| 802 | L4V6 | Skoura SKT1 | Morocco | INRAM Meknès | North-Africa | 3 |
| 803 | L4V7 | Skoura SKH1 | Morocco | INRAM Meknès | North-Africa | 3 |
| 804 | L4V8 | Outat Elhaj 7 | Morocco | INRAM Meknès | North-Africa | 4 |
| 805 | L4V9^*^ | Kalaat Meggouna G7 | Morocco | INRAM Meknès | North-Africa | mixed |
| 806 | L5V1^*^ | Goulmima GAY1 | Morocco | INRAM Meknès | North-Africa | 3 |
| 807 | L5V10^*^ | Outat Elhaj 6 | Morocco | INRAM Meknès | North-Africa | 3 |
| 808 | L5V11^*^ | Boumalen A2 | Morocco | INRAM Meknès | North-Africa | 3 |
| 809 | L5V2 | Goulmima GAY2 | Morocco | INRAM Meknès | North-Africa | 3 |
| 810 | L5V3 | Goulmima G3 | Morocco | INRAM Meknès | North-Africa | 3 |
| 811 | L5V4 | Missour V4 | Morocco | INRAM Meknès | North-Africa | mixed |
| 812 | L5V5 | Rtil 3 | Morocco | INRAM Meknès | North-Africa | 3 |
| 813 | L5V6 | Missour V2 | Morocco | INRAM Meknès | North-Africa | 3 |
| 814 | L5V8 | Outat Elhaj 2 | Morocco | INRAM Meknès | North-Africa | 5 |
| 815 | L5V9 | Outat Elhaj 3 | Morocco | INRAM Meknès | North-Africa | 5 |
| 816 | L6V1 | Marrouch 3 | Morocco | INRAM Meknès | North-Africa | 5 |
| 817 | L6V10 | Boumalen A1 | Morocco | INRAM Meknès | North-Africa | 3 |
| 818 | L6V11^*^ | Guersif 2 | Morocco | INRAM Meknès | North-Africa | 3 |
| 819 | L6V3 | Marrouch 16 | Morocco | INRAM Meknès | North-Africa | 5 |
| 820 | L6V4 | Marrouch 4 | Morocco | INRAM Meknès | North-Africa | 5 |
| 821 | L6V5 | Khorb | Morocco | INRAM Meknès | North-Africa | 3 |
| 822 | L6V6 | Maoui | Morocco | INRAM Meknès | North-Africa | 5 |
| 823 | L6V8 | Outat Elhaj1 | Morocco | INRAM Meknès | North-Africa | mixed |
| 824 | L6V9 | Kalaat Meggouna G8 | Morocco | INRAM Meknès | North-Africa | 3 |
| 825 | L7V1 | Gmat | Morocco | INRAM Meknès | North-Africa | 3 |
| 826 | L7V11^*^ | Skoura SKT5 | Morocco | INRAM Meknès | North-Africa | 3 |
| 827 | L7V2 | Mans | Morocco | INRAM Meknès | North-Africa | mixed |
| 828 | L7V3 | Del Patricia | Morocco | INRAM Meknès | North-Africa | 5 |
| 829 | L7V4 | Missour V17 | Morocco | INRAM Meknès | North-Africa | 3 |
| 830 | L7V6 | Boumalen KH1 | Morocco | INRAM Meknès | North-Africa | 3 |
| 831 | L7V7 | Outat Elhaj 8 | Morocco | INRAM Meknès | North-Africa | 5 |
| 832 | L7V8 | Agdez A5 | Morocco | INRAM Meknès | North-Africa | 3 |
| 833 | L8V1 | Missour V12 | Morocco | INRAM Meknès | North-Africa | 3 |
| 834 | L8V10 | Skoura SKH4 | Morocco | INRAM Meknès | North-Africa | 3 |
| 835 | L8V11 | SK1 | Morocco | INRAM Meknès | North-Africa | 5 |
| 836 | L8V12 | Agdez A4 | Morocco | INRAM Meknès | North-Africa | 3 |
| 837 | L8V15 | Agdez IG1 | Morocco | INRAM Meknès | North-Africa | 5 |
| 838 | L8V3^*^ | Rich RT1 | Morocco | INRAM Meknès | North-Africa | 5 |
| 839 | L8V4^*^ | Marrouch 1 | Morocco | INRAM Meknès | North-Africa | 3 |
| 840 | L8V5 | Missour V3 | Morocco | INRAM Meknès | North-Africa | mixed |
| 841 | L8V7^*^ | Missour V15 | Morocco | INRAM Meknès | North-Africa | mixed |
| 842 | L8V8^*^ | Kalaat Meggouna G9 | Morocco | INRAM Meknès | North-Africa | 3 |
| 843 | L8V9^*^ | Boumalen J2 | Morocco | INRAM Meknès | North-Africa | 3 |
| 844 | L9V1 | Missour V22 | Morocco | INRAM Meknès | North-Africa | 3 |
| 845 | L9V3 | Kalaat Meggouna G3 | Morocco | INRAM Meknès | North-Africa | 3 |
| 846 | 12PB407 | Perfection | USA | USDA ARS Parlier | America | mixed |
| 847 | 12PB408^*^ | Morden 604 | USA | USDA ARS Parlier | America | 2 |
| 848 | 12PB409^*^ | Sun Glo | USA | USDA ARS Parlier | America | 2 |
| 849 | 12PB410^*^ | Reliable | USA | USDA ARS Parlier | America | mixed |
| 850 | 12PB598 | A157 | France | INRA CRB GAFL | America | 5 |
| 851 | 12PB691^*^ | 1384-2 | USA | USDA ARS Parlier | America | 2 |
| 852 | 12PB692^*^ | 1393-5 | USA | USDA ARS Parlier | America | 2 |
| 853 | 12PB693^*^ | 1394-1 | USA | USDA ARS Parlier | America | 2 |
| 854 | 12PB694^*^ | 1395-1 | USA | USDA ARS Parlier | America | mixed |
| 855 | 12PB695^*^ | 1416-4 | USA | USDA ARS Parlier | America | mixed |
| 856 | A1145 | Seo | France | INRA CRB GAFL | America | 2 |
| 857 | A1145^*^ | Stark Early Orange faux | France | INRA CRB GAFL | America | mixed |
| 858 | A1170^*^ | SR7 | France | INRA CRB GAFL | America | mixed |
| 859 | A1608 | Good Sweet Kernel EM1 | France | INRA CRB GAFL | America | mixed |
| 860 | A1609 | Sophia | France | INRA CRB GAFL | America | mixed |
| 861 | A1717 | Early Orange | France | INRA CRB GAFL | America | 2 |
| 862 | A1752^*^ | King | France | INRA CRB GAFL | America | mixed |
| 863 | A2382 | Stella | France | INRA CRB GAFL | America | mixed |
| 864 | A2633^*^ | Skaha | France | INRA CRB GAFL | America | mixed |
| 865 | A2634 | Perfection | France | INRA CRB GAFL | America | mixed |
| 866 | A2637^*^ | Riland | France | INRA CRB GAFL | America | mixed |
| 867 | A2848^*^ | Royalty | France | INRA CRB GAFL | America | mixed |
| 868 | A2853 | Dwarf | France | INRA CRB GAFL | America | mixed |
| 869 | A2923^*^ | Morocot | France | INRA CRB GAFL | America | mixed |
| 870 | A0044 | Royal | France | INRA CRB GAFL | America | 5 |
| 871 | A0519 | Suizo | France | INRA CRB GAFL | America | 5 |
| 872 | A0634^*^ | Henderson | France | INRA CRB GAFL | America | mixed |
| 873 | A0862^*^ | Pseudo Royal | France | INRA CRB GAFL | America | mixed |
| 874 | Tk62 | Stark Early Orange | Turkey | Malatya University | America | 2 |
| 875 | U0937^*^ | Brigantina | USA | USDA ARS Parlier | America | 2 |
| 876 | U0946 | Hemskirke | USA | USDA ARS Parlier | America | 5 |
| 877 | U1504 | Large Early Montgemet | USA | USDA ARS Parlier | America | 4 |
| 878 | U1505 | Maxson | USA | USDA ARS Parlier | America | 5 |
| 879 | U2375^*^ | Santa Clara Sweet | USA | USDA ARS Parlier | America | mixed |
| 880 | U2376 | Canada White | USA | USDA ARS Parlier | America | mixed |
| 881 | U2377 | Shalah | USA | USDA ARS Parlier | Irano-Caucasian | mixed |
| 882 | U2378 | Casa Soberanes | USA | USDA ARS Parlier | America | 5 |
| 883 | U2379 | Stephen's Favorite | USA | USDA ARS Parlier | America | mixed |
| 884 | U2380^*^ | Chenton | USA | USDA ARS Parlier | America | 2 |
| 885 | U2381^*^ | Nugget | USA | USDA ARS Parlier | America | mixed |
| 886 | U2382 | Derby Royal | USA | USDA ARS Parlier | America | 5 |
| 887 | U2383 | Basque | USA | USDA ARS Parlier | America | mixed |
| 888 | U2384 | San Fernando Supreme | USA | USDA ARS Parlier | America | mixed |
| 889 | U2385^*^ | Supkany | USA | USDA ARS Parlier | Central Asia | mixed |
| 890 | U2424^*^ | CP 15-1 | USA | USDA ARS Parlier | America | mixed |
