# Supplementary material for: Genetic Structure of a Worldwide Germplasm Collection of Prunus armeniaca L. Reveals Three Major Diffusion Routes for Varieties Coming From the Species’ Center of Origin
Source: Front Plant Sci. 2020 May 25;11:638. doi: 10.3389/fpls.2020.00638 (PMC7261834; doi:10.3389/fpls.2020.00638)
Supplement: ADDITIONAL FILE S1 — List of the 890 apricot accessions considered in the present study. Accession code, name, site of collection, geographical group of origin, cluster assignment inferred by the STRUCTURE analysis are reported. Accessions with asterisk are core collections members. [file Data_Sheet_1.zip › Additional File 6.docx]

| Additional file 6. Membership of apricot accessions to the five clusters as determined by STRUCTURE analysis. | | | | | |  |  |  |
| --- | --- | --- | --- | --- | --- | --- | --- | --- |
|  |  |  |  |  |  |  |  |  |
| N° | Accession Name | Accession code | Geographic group of origin | Cluster | | | | |
|  |  |  |  | Cluster 1 (44) | Cluster 2 (149) | Cluster 3 (163) | Cluster 4 (64) | Cluster 5 (170) |
| 1 | Bansei Oumi | 25872 | Eastern Asia | 0.9867 | 0.0084 | 0.0017 | 0.0015 | 0.0018 |
| 2 | Heiwa | 25882 | Eastern Asia | 0.9782 | 0.0160 | 0.0026 | 0.0015 | 0.0017 |
| 3 | Hiroshima Koanzu | 25883 | Eastern Asia | 0.9759 | 0.0177 | 0.0022 | 0.0027 | 0.0016 |
| 4 | Jin Kyou | 25884 | Eastern Asia | 0.0072 | 0.9643 | 0.0054 | 0.0206 | 0.0025 |
| 5 | Jin Zhou Da Xing | 25885 | Eastern Asia | 0.9880 | 0.0044 | 0.0023 | 0.0036 | 0.0017 |
| 6 | Jinshirou | 25886 | Eastern Asia | 0.9692 | 0.0250 | 0.0020 | 0.0021 | 0.0018 |
| 7 | Kinrei | 25887 | Eastern Asia | 0.9919 | 0.0030 | 0.0018 | 0.0017 | 0.0015 |
| 8 | Koushiu Oumi | 25888 | Eastern Asia | 0.9924 | 0.0029 | 0.0016 | 0.0016 | 0.0015 |
| 9 | Niigata Oumi | 25890 | Eastern Asia | 0.9937 | 0.0017 | 0.0016 | 0.0015 | 0.0015 |
| 10 | Ogasawara | 25891 | Eastern Asia | 0.9622 | 0.0287 | 0.0031 | 0.0025 | 0.0035 |
| 11 | Okitama | 25892 | Eastern Asia | 0.9856 | 0.0081 | 0.0022 | 0.0022 | 0.0018 |
| 12 | Satakemaru | 25896 | Eastern Asia | 0.9465 | 0.0443 | 0.0053 | 0.0017 | 0.0021 |
| 13 | Satsuki | 25897 | Eastern Asia | 0.9896 | 0.0041 | 0.0023 | 0.0021 | 0.0019 |
| 14 | Shimizugo | 25898 | Eastern Asia | 0.8505 | 0.1432 | 0.0028 | 0.0018 | 0.0018 |
| 15 | Shinheiwa | 25899 | Eastern Asia | 0.9866 | 0.0058 | 0.0033 | 0.0019 | 0.0024 |
| 16 | Shiromomo Kitajima | 25900 | Eastern Asia | 0.9341 | 0.0594 | 0.0024 | 0.0020 | 0.0021 |
| 17 | Shiromomo Kondousanae | 25901 | Eastern Asia | 0.9775 | 0.0159 | 0.0030 | 0.0021 | 0.0016 |
| 18 | Takano Manjiu | 25902 | Eastern Asia | 0.9605 | 0.0162 | 0.0121 | 0.0041 | 0.0071 |
| 19 | Todoroki Manjiu | 25904 | Eastern Asia | 0.9386 | 0.0551 | 0.0024 | 0.0018 | 0.0020 |
| 20 | Tokoumaru | 25905 | Eastern Asia | 0.9881 | 0.0053 | 0.0027 | 0.0017 | 0.0023 |
| 21 | Toua | 25906 | Eastern Asia | 0.9890 | 0.0045 | 0.0024 | 0.0022 | 0.0020 |
| 22 | Yamagata 3 | 25908 | Eastern Asia | 0.9664 | 0.0190 | 0.0108 | 0.0018 | 0.0019 |
| 23 | Mikanmomo | 25912 | Eastern Asia | 0.9873 | 0.0045 | 0.0032 | 0.0029 | 0.0021 |
| 24 | Chinese | A2852 | Eastern Asia | 0.0018 | 0.0169 | 0.0030 | 0.9755 | 0.0028 |
| 25 | Čína | B10 | Eastern Asia | 0.0111 | 0.9792 | 0.0035 | 0.0033 | 0.0028 |
| 26 | Chuang Sin | B2 | Eastern Asia | 0.0062 | 0.9639 | 0.0045 | 0.0202 | 0.0051 |
| 27 | Moi-Chua-Sin | B6 | Eastern Asia | 0.4189 | 0.5632 | 0.0036 | 0.0109 | 0.0034 |
| 28 | In-Bej-Sin | B9 | Eastern Asia | 0.4689 | 0.5245 | 0.0026 | 0.0019 | 0.0021 |
| 29 | Ansu | C12 | Eastern Asia | 0.9041 | 0.0878 | 0.0028 | 0.0026 | 0.0028 |
| 30 | Šantungská | D1 | Eastern Asia | 0.0136 | 0.9683 | 0.0089 | 0.0047 | 0.0046 |
| 31 | Da-Yu-bada | D2 | Eastern Asia | 0.0452 | 0.9469 | 0.0029 | 0.0023 | 0.0028 |
| 32 | Mai-Che-Sin | D6 | Eastern Asia | 0.5115 | 0.4722 | 0.0039 | 0.0086 | 0.0039 |
| 33 | Mai-Huang | D7 | Eastern Asia | 0.0108 | 0.8956 | 0.0109 | 0.0373 | 0.0454 |
| 34 | 14 B | E10 | Eastern Asia | 0.0301 | 0.9558 | 0.0041 | 0.0036 | 0.0063 |
| 35 | He Bao Xing | E12 | Eastern Asia | 0.2172 | 0.5016 | 0.0038 | 0.0035 | 0.2740 |
| 36 | Pastyrik | E2 | Eastern Asia | 0.0077 | 0.8137 | 0.0194 | 0.0069 | 0.1522 |
| 37 | Yin Xang Bai Xing | E7 | Eastern Asia | 0.0625 | 0.9097 | 0.0072 | 0.0031 | 0.0175 |
| 38 | 5 Liaoning | E8 | Eastern Asia | 0.0160 | 0.9654 | 0.0127 | 0.0025 | 0.0034 |
| 39 | 3 B | E9 | Eastern Asia | 0.0026 | 0.9886 | 0.0035 | 0.0025 | 0.0028 |
| 40 | Hong Yu | F1 | Eastern Asia | 0.0039 | 0.5097 | 0.0142 | 0.0060 | 0.4661 |
| 41 | Dan Xing Bian Xing | F2 | Eastern Asia | 0.0205 | 0.9680 | 0.0046 | 0.0034 | 0.0035 |
| 42 | Sha Jin Hong | F3 | Eastern Asia | 0.0109 | 0.9689 | 0.0079 | 0.0057 | 0.0065 |
| 43 | 3 Liaoning | F4 | Eastern Asia | 0.0100 | 0.9743 | 0.0039 | 0.0059 | 0.0059 |
| 44 | L Liaoning | F6 | Eastern Asia | 0.0123 | 0.9698 | 0.0043 | 0.0059 | 0.0077 |
| 45 | Beixan Heda Huang | F7 | Eastern Asia | 0.1100 | 0.8229 | 0.0056 | 0.0043 | 0.0572 |
| 46 | Liaoning | F8 | Eastern Asia | 0.0059 | 0.9817 | 0.0053 | 0.0028 | 0.0044 |
| 47 | ČLR 1 | G10 | Eastern Asia | 0.0051 | 0.9527 | 0.0244 | 0.0059 | 0.0119 |
| 48 | ČLR Da Jie Xing | G2 | Eastern Asia | 0.0542 | 0.9321 | 0.0071 | 0.0021 | 0.0045 |
| 49 | ČLR 8 | G4 | Eastern Asia | 0.0068 | 0.9816 | 0.0027 | 0.0028 | 0.0062 |
| 50 | ČLR 4 | G5 | Eastern Asia | 0.0049 | 0.9623 | 0.0176 | 0.0048 | 0.0104 |
| 51 | ČLR 10 | G6 | Eastern Asia | 0.0473 | 0.9465 | 0.0024 | 0.0017 | 0.0021 |
| 52 | Chuan Zhi Hong | H3 | Eastern Asia | 0.0198 | 0.9721 | 0.0029 | 0.0021 | 0.0031 |
| 53 | Kanko-bai | U1588 | Eastern Asia | 0.0112 | 0.9814 | 0.0024 | 0.0025 | 0.0024 |
| 54 | 96077 | U2311 | Eastern Asia | 0.0254 | 0.9657 | 0.0031 | 0.0021 | 0.0036 |
| 55 | RRS-1A | U2346 | Eastern Asia | 0.0025 | 0.0030 | 0.0136 | 0.4336 | 0.5472 |
| 56 | Shanlian | U2426 | Eastern Asia | 0.0042 | 0.9888 | 0.0027 | 0.0018 | 0.0025 |
| 57 | Da-bei | JM1 | Eastern Asia | 0.0134 | 0.4859 | 0.0125 | 0.4734 | 0.0149 |
| 58 | Da-chuan-che N1 | JM2 | Eastern Asia | 0.0141 | 0.9755 | 0.0043 | 0.0033 | 0.0028 |
| 59 | Da-chuan-che N2 | JM3 | Eastern Asia | 0.0056 | 0.9472 | 0.0232 | 0.0151 | 0.0089 |
| 60 | Kitaiskii | JM4 | Eastern Asia | 0.0143 | 0.8185 | 0.0173 | 0.1198 | 0.0301 |
| 61 | Lao-yech-lian | JM5 | Eastern Asia | 0.0046 | 0.4331 | 0.0782 | 0.4587 | 0.0253 |
| 62 | ln-ben-sin | JM6 | Eastern Asia | 0.0046 | 0.9455 | 0.0152 | 0.0079 | 0.0267 |
| 63 | Mai-che-sin | JM7 | Eastern Asia | 0.0017 | 0.0017 | 0.0027 | 0.9911 | 0.0029 |
| 64 | Mi-bada | JM8 | Eastern Asia | 0.0018 | 0.3523 | 0.0086 | 0.5874 | 0.0499 |
| 65 | Pui-sha-sin | JM9 | Eastern Asia | 0.0023 | 0.8657 | 0.0190 | 0.0471 | 0.0659 |
| 66 | Shantunski | JM10 | Eastern Asia | 0.0089 | 0.9746 | 0.0074 | 0.0043 | 0.0049 |
| 67 | Yuan-sin | JM11 | Eastern Asia | 0.0138 | 0.9744 | 0.0050 | 0.0039 | 0.0029 |
| 68 | Kečpšar | A11 | Central Asia | 0.0030 | 0.9684 | 0.0100 | 0.0124 | 0.0062 |
| 69 | Oranzeno Krasnyj | A1275 | Central Asia | 0.0493 | 0.9110 | 0.0175 | 0.0107 | 0.0115 |
| 70 | Mitzourinskii2 | A1333 | Central Asia | 0.0057 | 0.9798 | 0.0052 | 0.0040 | 0.0053 |
| 71 | Nikitski | A1693 | Europe | 0.0094 | 0.1816 | 0.0071 | 0.0035 | 0.7984 |
| 72 | Molodoi | A2350 | Central Asia | 0.0030 | 0.0043 | 0.0143 | 0.0030 | 0.9755 |
| 73 | G1 A2039 | A3075 | Central Asia | 0.0045 | 0.3774 | 0.0862 | 0.5228 | 0.0091 |
| 74 | Ladah | A4082 | Central Asia | 0.0255 | 0.9366 | 0.0123 | 0.0086 | 0.0170 |
| 75 | Roxana | B3 | Central Asia | 0.0034 | 0.4585 | 0.0047 | 0.2316 | 0.3019 |
| 76 | Zard | D8 | Central Asia | 0.0214 | 0.9632 | 0.0056 | 0.0059 | 0.0039 |
| 77 | Oranževo Krasnyj | D9 | Central Asia | 0.0035 | 0.9792 | 0.0061 | 0.0077 | 0.0035 |
| 78 | Monti Ladak | It59 | Central Asia | 0.0017 | 0.0017 | 0.0027 | 0.9911 | 0.0028 |
| 79 | Zard | Tk65 | Central Asia | 0.0038 | 0.9671 | 0.0144 | 0.0078 | 0.0069 |
| 80 | Oranzhevo Krassny | U0341 | Central Asia | 0.0244 | 0.9426 | 0.0169 | 0.0056 | 0.0106 |
| 81 | Khubani | U1372 | Central Asia | 0.0108 | 0.9271 | 0.0107 | 0.0443 | 0.0071 |
| 82 |  | U1374 | Central Asia | 0.0051 | 0.9687 | 0.0142 | 0.0042 | 0.0077 |
| 83 | Kabuli | U1377 | Central Asia | 0.0135 | 0.0817 | 0.0159 | 0.0108 | 0.8781 |
| 84 | Habiju | U1380 | Central Asia | 0.0035 | 0.9609 | 0.0141 | 0.0046 | 0.0169 |
| 85 | Habiju | U1381 | Central Asia | 0.0028 | 0.7080 | 0.0312 | 0.0061 | 0.2520 |
| 86 | Janjir | U1383 | Central Asia | 0.0059 | 0.9014 | 0.0737 | 0.0118 | 0.0073 |
| 87 | Habiju | U1384 | Central Asia | 0.0039 | 0.7928 | 0.1309 | 0.0043 | 0.0681 |
| 88 | Mirmamudi I | U1385 | Central Asia | 0.0041 | 0.9179 | 0.0534 | 0.0169 | 0.0078 |
| 89 | Khuban | U1386 | Central Asia | 0.0035 | 0.5503 | 0.0925 | 0.0073 | 0.3463 |
| 90 | Gaqai Shikanda | U1393 | Central Asia | 0.0033 | 0.9452 | 0.0042 | 0.0142 | 0.0332 |
| 91 | Burum Gakas | U1394 | Central Asia | 0.0045 | 0.9804 | 0.0085 | 0.0020 | 0.0046 |
| 92 | Badam Gakas | U1395 | Central Asia | 0.0048 | 0.8474 | 0.0840 | 0.0038 | 0.0599 |
| 93 | Bulbil-e-shikanda | U1396 | Central Asia | 0.0034 | 0.8059 | 0.0222 | 0.0061 | 0.1624 |
| 94 | Kartashi | U1398 | Central Asia | 0.0034 | 0.9176 | 0.0111 | 0.0045 | 0.0633 |
| 95 | Quropo | U1401 | Central Asia | 0.0050 | 0.8350 | 0.0759 | 0.0070 | 0.0771 |
| 96 | Dail Mesh | U1402 | Central Asia | 0.0033 | 0.6804 | 0.2919 | 0.0041 | 0.0203 |
| 97 | Lakesh | U1403 | Central Asia | 0.0053 | 0.9555 | 0.0118 | 0.0106 | 0.0168 |
| 98 | Dolato Ju | U1404 | Central Asia | 0.0041 | 0.9465 | 0.0277 | 0.0055 | 0.0161 |
| 99 | Dolato Ju | U1405 | Central Asia | 0.0026 | 0.8667 | 0.0873 | 0.0039 | 0.0395 |
| 100 | Garkmish | U1407 | Central Asia | 0.0023 | 0.9779 | 0.0042 | 0.0031 | 0.0123 |
| 101 | Nagarum Shikanda | U1408 | Central Asia | 0.0052 | 0.9226 | 0.0066 | 0.0524 | 0.0132 |
| 102 | Gagai Tinani | U1409 | Central Asia | 0.0128 | 0.8120 | 0.1425 | 0.0239 | 0.0089 |
| 103 | Nazarali Kutzay Tinani | U1410 | Central Asia | 0.0029 | 0.9095 | 0.0281 | 0.0379 | 0.0216 |
| 104 | Shai Kutzay Ju | U1411 | Central Asia | 0.0022 | 0.9219 | 0.0256 | 0.0226 | 0.0277 |
| 105 | Arghoon | U1414 | Central Asia | 0.0030 | 0.9675 | 0.0175 | 0.0050 | 0.0070 |
| 106 | Khoshenda | U1416 | Central Asia | 0.0048 | 0.9755 | 0.0032 | 0.0072 | 0.0094 |
| 107 | Almon | U1419 | Central Asia | 0.0047 | 0.8642 | 0.0125 | 0.0059 | 0.1126 |
| 108 | Karfochuli | U1421 | Central Asia | 0.0032 | 0.9490 | 0.0196 | 0.0193 | 0.0088 |
| 109 | Korkum Chuli | U1423 | Central Asia | 0.0312 | 0.8343 | 0.0297 | 0.0859 | 0.0189 |
| 110 | Kachachuli | U1426 | Central Asia | 0.0121 | 0.9582 | 0.0174 | 0.0031 | 0.0093 |
| 111 | Badamchuli | U1427 | Central Asia | 0.0065 | 0.9637 | 0.0062 | 0.0107 | 0.0129 |
| 112 | Chaksa | U1428 | Central Asia | 0.0137 | 0.9656 | 0.0043 | 0.0127 | 0.0038 |
| 113 | Hashimpa | U1429 | Central Asia | 0.0056 | 0.9824 | 0.0038 | 0.0028 | 0.0054 |
| 114 | Khakas | U1430 | Central Asia | 0.0104 | 0.9768 | 0.0059 | 0.0026 | 0.0044 |
| 115 | Habiju | U1432 | Central Asia | 0.0147 | 0.9108 | 0.0447 | 0.0075 | 0.0223 |
| 116 | Shikanda | U1433 | Central Asia | 0.0129 | 0.9511 | 0.0217 | 0.0067 | 0.0077 |
| 117 |  | U1434 | Central Asia | 0.0027 | 0.5054 | 0.4656 | 0.0177 | 0.0086 |
| 118 |  | U1435 | Central Asia | 0.0073 | 0.9434 | 0.0226 | 0.0167 | 0.0100 |
| 119 | Soris | U1436 | Central Asia | 0.0042 | 0.9785 | 0.0045 | 0.0043 | 0.0085 |
| 120 | Paiwand | U1438 | Central Asia | 0.0027 | 0.9352 | 0.0210 | 0.0045 | 0.0366 |
| 121 |  | U1439 | Central Asia | 0.0061 | 0.9788 | 0.0042 | 0.0055 | 0.0054 |
| 122 | Luchak #6 | U1795 | Central Asia | 0.0029 | 0.9080 | 0.0419 | 0.0104 | 0.0368 |
| 123 | USSR 90-04-01 | U1799 | Central Asia | 0.0044 | 0.9713 | 0.0087 | 0.0096 | 0.0061 |
| 124 | Yhulag | U1800 | Central Asia | 0.0047 | 0.9505 | 0.0128 | 0.0101 | 0.0219 |
| 125 | Mahtoby | U1801 | Central Asia | 0.0029 | 0.9569 | 0.0131 | 0.0115 | 0.0156 |
| 126 |  | U1802 | Central Asia | 0.0039 | 0.9029 | 0.0604 | 0.0061 | 0.0267 |
| 127 | USSR 90-11-01 | U1803 | Central Asia | 0.0050 | 0.9748 | 0.0070 | 0.0048 | 0.0085 |
| 128 | USSR 90-13-03 | U1805 | Central Asia | 0.0018 | 0.9907 | 0.0029 | 0.0019 | 0.0028 |
| 129 |  | U1807 | Central Asia | 0.0039 | 0.9495 | 0.0072 | 0.0164 | 0.0230 |
| 130 |  | U1809 | Central Asia | 0.0054 | 0.9040 | 0.0510 | 0.0309 | 0.0087 |
| 131 | USSR 90-16-04 | U1810 | Central Asia | 0.0049 | 0.9763 | 0.0039 | 0.0117 | 0.0032 |
| 132 | USSR 90-16-05 | U1811 | Central Asia | 0.0042 | 0.9683 | 0.0192 | 0.0044 | 0.0040 |
| 133 | USSR 90-16-06 | U1812 | Central Asia | 0.0139 | 0.9247 | 0.0093 | 0.0351 | 0.0170 |
| 134 | USSR 90-15-04 | U1900 | Central Asia | 0.0076 | 0.5379 | 0.0146 | 0.4236 | 0.0164 |
| 135 | USSR 90-16-03 | U1901 | Central Asia | 0.0095 | 0.9670 | 0.0061 | 0.0126 | 0.0048 |
| 136 | Afghanistan E43-12 | U2085 | Central Asia | 0.0071 | 0.0420 | 0.9435 | 0.0039 | 0.0034 |
| 137 | USSR-90-06-01 | U2279 | Central Asia | 0.0048 | 0.9519 | 0.0116 | 0.0092 | 0.0224 |
| 138 | Ak Luchak | U2286 | Central Asia | 0.0028 | 0.4816 | 0.4376 | 0.0069 | 0.0711 |
| 139 |  | U2307 | Central Asia | 0.0117 | 0.9607 | 0.0088 | 0.0106 | 0.0081 |
| 140 |  | U2308 | Central Asia | 0.0032 | 0.9867 | 0.0036 | 0.0040 | 0.0025 |
| 141 |  | U2309 | Central Asia | 0.0036 | 0.9867 | 0.0032 | 0.0032 | 0.0032 |
| 142 |  | U2310 | Central Asia | 0.0239 | 0.9501 | 0.0173 | 0.0023 | 0.0064 |
| 143 | Arzamy 37 | U2390 | Central Asia | 0.0056 | 0.5140 | 0.0043 | 0.0047 | 0.4714 |
| 144 | N262 | U2391 | Central Asia | 0.0100 | 0.9818 | 0.0032 | 0.0026 | 0.0024 |
| 145 | N270 | U2393 | Central Asia | 0.0605 | 0.9172 | 0.0171 | 0.0017 | 0.0036 |
| 146 | 280 | U2394 | Central Asia | 0.2726 | 0.7054 | 0.0143 | 0.0024 | 0.0053 |
| 147 | 14336 | U2395 | Central Asia | 0.0241 | 0.7875 | 0.0993 | 0.0701 | 0.0190 |
| 148 | Tza-Sin | U2422 | Central Asia | 0.0059 | 0.2692 | 0.0109 | 0.6799 | 0.0340 |
| 149 | Da-Dze-Sin | U2423 | Central Asia | 0.0640 | 0.5002 | 0.0717 | 0.0042 | 0.3599 |
| 150 | Badjok 93 | U2425 | Central Asia | 0.0039 | 0.9736 | 0.0077 | 0.0088 | 0.0060 |
| 151 | IC 20089 | U2550 | Central Asia | 0.0025 | 0.3870 | 0.5742 | 0.0313 | 0.0049 |
| 152 | Subhany | U2553 | Central Asia | 0.0032 | 0.1331 | 0.8503 | 0.0076 | 0.0058 |
| 153 | Germab-5 | U2570 | Central Asia | 0.0103 | 0.4758 | 0.0919 | 0.3940 | 0.0279 |
| 154 | Birleshik 2 | U2590 | Central Asia | 0.0026 | 0.0697 | 0.4510 | 0.2564 | 0.2202 |
| 155 | Arzami | JM12 | Central Asia | 0.0033 | 0.9714 | 0.0056 | 0.0115 | 0.0082 |
| 156 | B-1-11 | JM13 | Central Asia | 0.0080 | 0.6283 | 0.2622 | 0.0108 | 0.0908 |
| 157 | B-4-5 | JM14 | Central Asia | 0.0062 | 0.8710 | 0.0810 | 0.0189 | 0.0229 |
| 158 | B-5-3 | JM15 | Central Asia | 0.0055 | 0.4443 | 0.5132 | 0.0203 | 0.0166 |
| 159 | Badami | JM16 | Central Asia | 0.0064 | 0.8427 | 0.1138 | 0.0027 | 0.0344 |
| 160 | Dionis | JM17 | Irano-Caucasian/Central Asia | 0.0450 | 0.1933 | 0.5456 | 0.1675 | 0.0486 |
| 161 | Gulyunghi | JM18 | Central Asia | 0.0054 | 0.6950 | 0.0431 | 0.0130 | 0.2435 |
| 162 | Iskadari | JM19 | Central Asia | 0.0026 | 0.9873 | 0.0039 | 0.0023 | 0.0039 |
| 163 | Kandak-10 | JM20 | Central Asia | 0.7505 | 0.2374 | 0.0053 | 0.0022 | 0.0045 |
| 164 | Kandak-12 | JM21 | Central Asia | 0.0027 | 0.4184 | 0.0064 | 0.5696 | 0.0029 |
| 165 | KB-12 | JM22 | Central Asia | 0.0055 | 0.8174 | 0.0567 | 0.0142 | 0.1062 |
| 166 | KB-9 | JM23 | Central Asia | 0.0025 | 0.8985 | 0.0295 | 0.0217 | 0.0478 |
| 167 | Khurmai | JM24 | Central Asia | 0.0051 | 0.9505 | 0.0215 | 0.0028 | 0.0201 |
| 168 | Khurmai Rannii | JM25 | Central Asia | 0.0047 | 0.9457 | 0.0153 | 0.0078 | 0.0265 |
| 169 | KK(2) N1 | JM26 | Central Asia | 0.0065 | 0.5919 | 0.3945 | 0.0020 | 0.0051 |
| 170 | Kok-pshar | JM27 | Central Asia | 0.0119 | 0.9779 | 0.0055 | 0.0024 | 0.0023 |
| 171 | Kolon Boboi | JM28 | Central Asia | 0.0037 | 0.8752 | 0.0643 | 0.0027 | 0.0541 |
| 172 | Krimskii Amur | JM29 | Central Asia/Europe | 0.0779 | 0.5761 | 0.0094 | 0.0055 | 0.3311 |
| 173 | Kunduzi | JM30 | Central Asia | 0.0075 | 0.9485 | 0.0216 | 0.0036 | 0.0188 |
| 174 | Kzyl Khorezmskii | JM31 | Central Asia | 0.0072 | 0.5036 | 0.4567 | 0.0045 | 0.0280 |
| 175 | Kzyl Khurmai Kannibadam | JM32 | Central Asia | 0.0161 | 0.9483 | 0.0110 | 0.0117 | 0.0129 |
| 176 | Kzyl Uryuk | JM33 | Central Asia | 0.0055 | 0.9439 | 0.0098 | 0.0182 | 0.0226 |
| 177 | Lunnik | JM34 | Europe/Irano-Caucasian | 0.0047 | 0.9566 | 0.0092 | 0.0221 | 0.0074 |
| 178 | Lyuichak Sumbarski | JM35 | Central Asia | 0.0020 | 0.1674 | 0.4299 | 0.3884 | 0.0123 |
| 179 | Maftobi | JM36 | Central Asia | 0.0050 | 0.7091 | 0.2263 | 0.0203 | 0.0392 |
| 180 | Medunets Krimskii | JM37 | Central Asia/Europe | 0.0019 | 0.3887 | 0.0089 | 0.5596 | 0.0409 |
| 181 | Mirsandzhali | JM38 | Central Asia | 0.0044 | 0.5513 | 0.0180 | 0.2774 | 0.1489 |
| 182 | Naryadnyi | JM39 | Central Asia/Europe | 0.0023 | 0.4332 | 0.4450 | 0.0073 | 0.1122 |
| 183 | Naslazhdenije | JM40 | Europe/Irano-Caucasian | 0.0041 | 0.9816 | 0.0030 | 0.0021 | 0.0093 |
| 184 | Nukul Citronnyi | JM41 | Central Asia | 0.0050 | 0.7460 | 0.2260 | 0.0164 | 0.0065 |
| 185 | Dzhungarskii 18/55 | JM42 | Central Asia | 0.0056 | 0.6471 | 0.3358 | 0.0050 | 0.0066 |
| 186 | Dzhungarskii 18/63 | JM43 | Central Asia | 0.0110 | 0.9363 | 0.0053 | 0.0431 | 0.0043 |
| 187 | Dzhungarskii 18/64 | JM44 | Central Asia | 0.0033 | 0.9610 | 0.0221 | 0.0026 | 0.0109 |
| 188 | Dzhungarskii 18/68 | JM45 | Central Asia | 0.0045 | 0.7137 | 0.2677 | 0.0043 | 0.0099 |
| 189 | Dzhungarskii 18/75 | JM46 | Central Asia | 0.0031 | 0.3161 | 0.4583 | 0.0761 | 0.1464 |
| 190 | Dzhungarskii 18/78 | JM47 | Central Asia | 0.0051 | 0.9218 | 0.0443 | 0.0061 | 0.0228 |
| 191 | *P. ansu* | JM48 | China | 0.0070 | 0.9855 | 0.0023 | 0.0026 | 0.0026 |
| 192 | *P. brigantina* | JM49 | Alpine plum (outgroup) | 0.8878 | 0.1001 | 0.0050 | 0.0039 | 0.0031 |
| 193 | *P. brigantina* x *P. armeniaca* (Olimp) | JM50 | Interspecific hybrid (outgroup) | 0.0100 | 0.9806 | 0.0035 | 0.0024 | 0.0035 |
| 194 | *P. brigantina* x *P. cerasifera* (Pionerka) | JM51 | Interspecific hybrid (outgroup) | 0.0200 | 0.9701 | 0.0035 | 0.0023 | 0.0041 |
| 195 | *P. Davida* N 7 (peach) | JM52 | *Prunus davidiana* (outgroup) | 0.0026 | 0.9862 | 0.0060 | 0.0017 | 0.0034 |
| 196 | *P. Davida* N 8 (peach) | JM53 | *Prunus davidiana* (outgroup) | 0.3374 | 0.6546 | 0.0038 | 0.0019 | 0.0024 |
| 197 | *P. mume* N15 | JM54 | China | 0.0071 | 0.9692 | 0.0043 | 0.0148 | 0.0047 |
| 198 | *P. mume* N18 | JM55 | China | 0.0029 | 0.8404 | 0.0223 | 0.0101 | 0.1243 |
| 199 | *P. sibirica* var *davidiana* | JM56 | Central Asia (Russia) | 0.0032 | 0.9837 | 0.0073 | 0.0020 | 0.0039 |
| 200 | Paivandi Bukharskii | JM57 | Central Asia | 0.0041 | 0.4358 | 0.5365 | 0.0060 | 0.0176 |
| 201 | Parnas | JM58 | European/Irano-Caucasian | 0.0038 | 0.9728 | 0.0062 | 0.0127 | 0.0045 |
| 202 | Priusadebnyi Rannii | JM59 | Central Asia | 0.0035 | 0.5904 | 0.1199 | 0.0100 | 0.2762 |
| 203 | Rukhi Dzhuvanon Meona | JM60 | Central Asia | 0.0023 | 0.9503 | 0.0273 | 0.0090 | 0.0112 |
| 204 | Rukhi Dzhuvanon Surkh | JM61 | Central Asia | 0.0070 | 0.8009 | 0.1051 | 0.0067 | 0.0803 |
| 205 | Samyi Rannii | JM62 | Central Asia | 0.0257 | 0.9659 | 0.0032 | 0.0026 | 0.0025 |
| 206 | Satser | JM63 | *P. armeniaca* x *P. sibirica* | 0.0019 | 0.1654 | 0.0051 | 0.2222 | 0.6054 |
| 207 | Supkhani | JM64 | Central Asia | 0.0139 | 0.9743 | 0.0049 | 0.0039 | 0.0030 |
| 208 | Tadzhabai | JM65 | Central Asia | 0.0023 | 0.4197 | 0.5551 | 0.0070 | 0.0158 |
| 209 | Zard | JM66 | Central Asia | 0.0020 | 0.1679 | 0.4298 | 0.3880 | 0.0123 |
| 210 | Kabaasi | 25847 | Irano-Caucasian | 0.0035 | 0.0348 | 0.8515 | 0.0148 | 0.0954 |
| 211 | Igdir | 25848 | Irano-Caucasian | 0.0024 | 0.0026 | 0.9827 | 0.0066 | 0.0056 |
| 212 | Mahmudun Erigi | 25849 | Irano-Caucasian | 0.0035 | 0.0099 | 0.9743 | 0.0066 | 0.0057 |
| 213 | Alyanak | 25850 | Irano-Caucasian | 0.0030 | 0.0028 | 0.0038 | 0.4275 | 0.5630 |
| 214 | Cologlu | 25864 | Irano-Caucasian | 0.0023 | 0.0029 | 0.9796 | 0.0066 | 0.0086 |
| 215 | Hasanbey | 25865 | Irano-Caucasian | 0.0029 | 0.0073 | 0.9509 | 0.0129 | 0.0260 |
| 216 | Choloulu | 25875 | Irano-Caucasian | 0.0082 | 0.0091 | 0.9664 | 0.0088 | 0.0075 |
| 217 | Hajihaliloulu | 25879 | Irano-Caucasian | 0.0024 | 0.0026 | 0.9827 | 0.0068 | 0.0056 |
| 218 | Hasanbay | 25881 | Irano-Caucasian | 0.0029 | 0.0028 | 0.9825 | 0.0056 | 0.0061 |
| 219 | Badami | A1267 | Irano-Caucasian | 0.0030 | 0.9719 | 0.0148 | 0.0023 | 0.0080 |
| 220 | Charhoud | A1406 | Central Asia | 0.0031 | 0.4485 | 0.5373 | 0.0065 | 0.0046 |
| 221 | Nassiri | A1458 | Irano-Caucasian | 0.0032 | 0.0023 | 0.0047 | 0.0054 | 0.9845 |
| 222 | Ordurao | A1868 | Irano-Caucasian | 0.0032 | 0.1020 | 0.4295 | 0.0066 | 0.4588 |
| 223 | Semis | A1871 | Irano-Caucasian | 0.4301 | 0.0044 | 0.0054 | 0.0026 | 0.5575 |
| 224 | Tokaloglu | A2166 | Irano-Caucasian | 0.1249 | 0.2716 | 0.0635 | 0.0114 | 0.5286 |
| 225 | Karakabey | A2292 | Irano-Caucasian | 0.0022 | 0.0019 | 0.0034 | 0.4434 | 0.5492 |
| 226 | Sam | A2294 | Irano-Caucasian | 0.0021 | 0.0024 | 0.0032 | 0.0065 | 0.9860 |
| 227 | Erevani | A2348 | Irano-Caucasian | 0.0032 | 0.0171 | 0.6519 | 0.0176 | 0.3101 |
| 228 | Dorosthe | A2609 | Irano-Caucasian | 0.0027 | 0.0171 | 0.5973 | 0.1708 | 0.2122 |
| 229 | Kermanshah | A2613 | Irano-Caucasian | 0.3959 | 0.1923 | 0.0172 | 0.0031 | 0.3915 |
| 230 | G1 2121 6 | A3508 | Irano-Caucasian | 0.0071 | 0.3521 | 0.6251 | 0.0042 | 0.0115 |
| 231 | G1 2121 7 | A3509 | Irano-Caucasian | 0.0050 | 0.3224 | 0.6610 | 0.0041 | 0.0075 |
| 232 | G1 2122 1 | A3512 | Irano-Caucasian | 0.0138 | 0.2902 | 0.6632 | 0.0178 | 0.0149 |
| 233 | G1 2122 5 | A3515 | Irano-Caucasian | 0.0053 | 0.5118 | 0.4246 | 0.0162 | 0.0421 |
| 234 | G1 2122 6 | A3516 | Irano-Caucasian | 0.0051 | 0.2005 | 0.5921 | 0.1915 | 0.0107 |
| 235 | G1 2122 7 | A3517 | Irano-Caucasian | 0.0026 | 0.3115 | 0.6755 | 0.0048 | 0.0057 |
| 236 | G1 2122 8 | A3518 | Irano-Caucasian | 0.0074 | 0.2332 | 0.7463 | 0.0087 | 0.0044 |
| 237 | G1 2122 11 | A3521 | Irano-Caucasian | 0.0089 | 0.1371 | 0.8261 | 0.0220 | 0.0059 |
| 238 | G1 2124 3 | A3522 | Irano-Caucasian | 0.0081 | 0.3166 | 0.6557 | 0.0054 | 0.0143 |
| 239 | G1 2053 3 | A3523 | Irano-Caucasian | 0.0026 | 0.1695 | 0.7940 | 0.0230 | 0.0109 |
| 240 | Moustakaoui | A578 | Irano-Caucasian | 0.0018 | 0.0020 | 0.0068 | 0.0039 | 0.9855 |
| 241 | Abu Talibu | B1 | Irano-Caucasian | 0.0033 | 0.6525 | 0.3259 | 0.0093 | 0.0089 |
| 242 | Lasgerdi Mashad | E1 | Irano-Caucasian | 0.0055 | 0.9064 | 0.0744 | 0.0084 | 0.0052 |
| 243 | Adilcevaz-5 | Tk1 | Irano-Caucasian | 0.0050 | 0.0151 | 0.9694 | 0.0054 | 0.0052 |
| 244 | Mahmudun Erigi | Tk10 | Irano-Caucasian | 0.0079 | 0.2729 | 0.0235 | 0.0152 | 0.6805 |
| 245 | Sekerpare | Tk11 | Irano-Caucasian | 0.0039 | 0.0105 | 0.9740 | 0.0071 | 0.0046 |
| 246 | Soganci | Tk12 | Irano-Caucasian | 0.0032 | 0.0167 | 0.6994 | 0.0054 | 0.2755 |
| 247 | Tokaloglu-Erzincan | Tk13 | Irano-Caucasian | 0.0036 | 0.2456 | 0.3049 | 0.0656 | 0.3804 |
| 248 | Tokaloglu-Yalova | Tk14 | Irano-Caucasian | 0.0020 | 0.0021 | 0.0034 | 0.0043 | 0.9883 |
| 249 | Inciaz Erigi | Tk15 | Irano-Caucasian | 0.0053 | 0.7254 | 0.2038 | 0.0465 | 0.0190 |
| 250 | Kayisi Erigi | Tk16 | Irano-Caucasian | 0.0033 | 0.9230 | 0.0552 | 0.0122 | 0.0064 |
| 251 | Agerik | Tk17 | Irano-Caucasian | 0.0047 | 0.0091 | 0.9490 | 0.0284 | 0.0088 |
| 252 | Alata Yildizi | Tk18 | Irano-Caucasian | 0.1346 | 0.1404 | 0.2617 | 0.0031 | 0.4602 |
| 253 | Alyanak | Tk19 | Irano-Caucasian | 0.0031 | 0.0028 | 0.0038 | 0.4273 | 0.5631 |
| 254 | Cataloglu | Tk2 | Irano-Caucasian | 0.0020 | 0.0117 | 0.9667 | 0.0083 | 0.0113 |
| 255 | Aprikoz | Tk20 | Irano-Caucasian | 0.0031 | 0.0193 | 0.6856 | 0.0139 | 0.2780 |
| 256 | Cagataybey | Tk21 | Irano-Caucasian | 0.4317 | 0.0040 | 0.5459 | 0.0126 | 0.0058 |
| 257 | Dr. Asma | Tk22 | Irano-Caucasian | 0.0107 | 0.5151 | 0.4619 | 0.0051 | 0.0071 |
| 258 | Guz Aprikozu | Tk23 | Irano-Caucasian | 0.0077 | 0.3229 | 0.6538 | 0.0023 | 0.0133 |
| 259 | Imrahor | Tk24 | Irano-Caucasian | 0.0043 | 0.0123 | 0.9354 | 0.0099 | 0.0381 |
| 260 | Iri Bitirgen | Tk25 | Irano-Caucasian | 0.0060 | 0.0126 | 0.9678 | 0.0034 | 0.0102 |
| 261 | Kamelya | Tk26 | Irano-Caucasian | 0.0084 | 0.3062 | 0.4907 | 0.1478 | 0.0469 |
| 262 | Karacabey | Tk27 | Irano-Caucasian | 0.0046 | 0.0098 | 0.9595 | 0.0181 | 0.0080 |
| 263 | Levent | Tk28 | Irano-Caucasian | 0.0084 | 0.0133 | 0.6772 | 0.1667 | 0.1343 |
| 264 | Sakit-1 | Tk29 | Irano-Caucasian | 0.0180 | 0.0063 | 0.9647 | 0.0057 | 0.0053 |
| 265 | Cologlu | Tk3 | Irano-Caucasian | 0.0019 | 0.0030 | 0.9762 | 0.0082 | 0.0107 |
| 266 | Sakit-2 | Tk30 | Irano-Caucasian | 0.0179 | 0.0062 | 0.9648 | 0.0057 | 0.0053 |
| 267 | Sakit-3 | Tk31 | Irano-Caucasian | 0.0043 | 0.0124 | 0.9350 | 0.0100 | 0.0384 |
| 268 | Sakit-4 | Tk32 | Irano-Caucasian | 0.0180 | 0.0061 | 0.9650 | 0.0057 | 0.0052 |
| 269 | Sakit-6 | Tk33 | Irano-Caucasian | 0.0047 | 0.0197 | 0.5825 | 0.0034 | 0.3896 |
| 270 | Sakit-7 | Tk34 | Irano-Caucasian | 0.0043 | 0.0204 | 0.9104 | 0.0136 | 0.0513 |
| 271 | Sam | Tk35 | Irano-Caucasian | 0.0020 | 0.0021 | 0.0033 | 0.0043 | 0.9882 |
| 272 | Torunoglu | Tk36 | Irano-Caucasian | 0.0108 | 0.1646 | 0.8001 | 0.0179 | 0.0066 |
| 273 | Turfanda Eskimalatya | Tk37 | Irano-Caucasian | 0.0028 | 0.0064 | 0.9447 | 0.0181 | 0.0280 |
| 274 | Ziraat Okulu | Tk38 | Irano-Caucasian | 0.0018 | 0.0022 | 0.0039 | 0.2236 | 0.7684 |
| 275 | Ordubat | Tk39 | Irano-Caucasian | 0.0032 | 0.1330 | 0.7384 | 0.0132 | 0.1122 |
| 276 | Hacihaliloglu | Tk5 | Irano-Caucasian | 0.0024 | 0.0029 | 0.9839 | 0.0062 | 0.0046 |
| 277 | Hacikiz | Tk6 | Irano-Caucasian | 0.0027 | 0.0683 | 0.5540 | 0.0225 | 0.3526 |
| 278 | Hasanbey | Tk7 | Irano-Caucasian | 0.0079 | 0.0811 | 0.8477 | 0.0335 | 0.0298 |
| 279 | Kabaasi | Tk8 | Irano-Caucasian | 0.0023 | 0.0030 | 0.9809 | 0.0077 | 0.0061 |
| 280 | Kurukabuk | Tk9 | Irano-Caucasian | 0.0027 | 0.0684 | 0.5544 | 0.0224 | 0.3521 |
| 281 |  | U1787 | Irano-Caucasian | 0.0026 | 0.0047 | 0.3046 | 0.1063 | 0.5817 |
| 282 |  | U1788 | Irano-Caucasian | 0.0164 | 0.2815 | 0.6341 | 0.0350 | 0.0330 |
| 283 |  | U1847 | Irano-Caucasian | 0.0035 | 0.9878 | 0.0019 | 0.0052 | 0.0017 |
| 284 | Lasgerdi Mashaad | U2065 | Irano-Caucasian | 0.0088 | 0.0406 | 0.9422 | 0.0051 | 0.0033 |
| 285 | Turkish White | U2095 | Irano-Caucasian | 0.0044 | 0.0131 | 0.5715 | 0.3208 | 0.0903 |
| 286 | Daradzhi ek Shabistr | JM67 | Irano-Caucasian | 0.0020 | 0.1050 | 0.0085 | 0.8811 | 0.0034 |
| 287 | Katuni | JM68 | Irano-Caucasian | 0.0019 | 0.0026 | 0.0327 | 0.1382 | 0.8247 |
| 288 | Kurbane Marache | JM69 | Irano-Caucasian | 0.0117 | 0.2268 | 0.7351 | 0.0149 | 0.0116 |
| 289 | Mascat | JM70 | Irano-Caucasian | 0.0031 | 0.0260 | 0.8038 | 0.1319 | 0.0352 |
| 290 | Nakhichevanskii | JM71 | Irano-Caucasian | 0.6284 | 0.3558 | 0.0054 | 0.0064 | 0.0039 |
| 291 | Nasera Tabris | JM72 | Irano-Caucasian | 0.0027 | 0.0126 | 0.9702 | 0.0088 | 0.0056 |
| 292 | Ordubad | JM73 | Irano-Caucasian | 0.0054 | 0.9651 | 0.0106 | 0.0036 | 0.0153 |
| 293 | Shalakh | JM74 | Irano-Caucasian | 0.0027 | 0.0024 | 0.0037 | 0.4859 | 0.5054 |
| 294 | Shekarpara de Semnan | JM75 | Irano-Caucasian | 0.0023 | 0.0812 | 0.8907 | 0.0050 | 0.0208 |
| 295 | Vaagas Vardaguin | JM76 | Irano-Caucasian | 0.0019 | 0.1134 | 0.0046 | 0.2460 | 0.6341 |
| 296 | Luizet | 3682 | Continental Europe | 0.0020 | 0.0019 | 0.0030 | 0.9902 | 0.0029 |
| 297 | Luizet | 3684 | Continental Europe | 0.0018 | 0.0017 | 0.0027 | 0.9911 | 0.0028 |
| 298 | Luizet | 3688 | Continental Europe | 0.0018 | 0.0017 | 0.0029 | 0.9904 | 0.0032 |
| 299 | Luizet | 3703 | Continental Europe | 0.0017 | 0.0017 | 0.0025 | 0.9915 | 0.0025 |
| 300 | Luizet | 3707 | Continental Europe | 0.0019 | 0.0018 | 0.0029 | 0.9905 | 0.0029 |
| 301 | Luizet | 3708 | Continental Europe | 0.0017 | 0.0017 | 0.0025 | 0.9914 | 0.0026 |
| 302 | Luizet | 3711 | Continental Europe | 0.0018 | 0.0017 | 0.0026 | 0.9912 | 0.0027 |
| 303 | Luizet | 3712 | Continental Europe | 0.0018 | 0.0017 | 0.0026 | 0.9913 | 0.0026 |
| 304 | Luizet | 3715 | Continental Europe | 0.0019 | 0.0019 | 0.0029 | 0.9903 | 0.0030 |
| 305 | Luizet | 3718 | Continental Europe | 0.0019 | 0.0018 | 0.0027 | 0.9910 | 0.0027 |
| 306 | Luizet | 3830 | Continental Europe | 0.0018 | 0.0017 | 0.0027 | 0.9908 | 0.0031 |
| 307 | Luizet | 3939 | Continental Europe | 0.0019 | 0.0019 | 0.0026 | 0.9908 | 0.0027 |
| 308 | Luizet | 3941 | Continental Europe | 0.0060 | 0.0032 | 0.0027 | 0.9855 | 0.0026 |
| 309 | Luizet | 3943 | Continental Europe | 0.0018 | 0.0017 | 0.0026 | 0.9912 | 0.0026 |
| 310 | Luizet | 3944 | Continental Europe | 0.0022 | 0.0395 | 0.0076 | 0.9469 | 0.0038 |
| 311 | Luizet | 3947 | Continental Europe | 0.0019 | 0.0162 | 0.0145 | 0.9644 | 0.0029 |
| 312 | Luizet | 3948 | Continental Europe | 0.0017 | 0.0017 | 0.0027 | 0.9911 | 0.0028 |
| 313 | Perrier | 3949 | Continental Europe | 0.0019 | 0.0018 | 0.0027 | 0.9909 | 0.0027 |
| 314 | Luizet | 3951 | Continental Europe | 0.0017 | 0.0017 | 0.0025 | 0.9914 | 0.0026 |
| 315 | Velcopavlovitsca | 25866 | Continental Europe | 0.0021 | 0.0019 | 0.0030 | 0.4139 | 0.5790 |
| 316 | Luizet | 3685a | Continental Europe | 0.0017 | 0.0017 | 0.0025 | 0.9915 | 0.0026 |
| 317 | Luizet | 3686a | Continental Europe | 0.0020 | 0.0019 | 0.0027 | 0.9906 | 0.0028 |
| 318 | Luizet | 3689a | Continental Europe | 0.0017 | 0.0017 | 0.0024 | 0.9917 | 0.0025 |
| 319 | Luizet | 3690b | Continental Europe | 0.0019 | 0.0017 | 0.0027 | 0.9910 | 0.0027 |
| 320 | Luizet | 3691a | Continental Europe | 0.0018 | 0.0018 | 0.0026 | 0.9912 | 0.0026 |
| 321 | Luizet | 3692a | Continental Europe | 0.0019 | 0.0041 | 0.0194 | 0.9520 | 0.0226 |
| 322 | Luizet | 3695a | Continental Europe | 0.0019 | 0.0018 | 0.0027 | 0.9910 | 0.0027 |
| 323 | Luizet | 3696a | Continental Europe | 0.0018 | 0.0018 | 0.0027 | 0.9909 | 0.0028 |
| 324 | Luizet | 3699a | Continental Europe | 0.0018 | 0.0017 | 0.0025 | 0.9914 | 0.0026 |
| 325 | Luizet | 3701a | Continental Europe | 0.0017 | 0.0017 | 0.0024 | 0.9917 | 0.0025 |
| 326 | Luizet | 3702a | Continental Europe | 0.0064 | 0.0034 | 0.0028 | 0.9847 | 0.0027 |
| 327 | Luizet | 3704a | Continental Europe | 0.0023 | 0.0202 | 0.0031 | 0.9716 | 0.0029 |
| 328 | Luizet | 3942a | Continental Europe | 0.0018 | 0.0017 | 0.0026 | 0.9914 | 0.0026 |
| 329 | Luizet | 3945a | Continental Europe | 0.0018 | 0.0017 | 0.0026 | 0.9913 | 0.0026 |
| 330 | Luizet | 3950a | Continental Europe | 0.0018 | 0.0017 | 0.0025 | 0.9914 | 0.0026 |
| 331 | Luizet | 3954a | Continental Europe | 0.0018 | 0.0018 | 0.0028 | 0.9906 | 0.0030 |
| 332 | Luizet | 3955a | Continental Europe | 0.0018 | 0.0018 | 0.0027 | 0.9910 | 0.0027 |
| 333 | Luizet | 3961a | Continental Europe | 0.0019 | 0.0018 | 0.0029 | 0.9907 | 0.0027 |
| 334 | Luizet | 3966a | Continental Europe | 0.0023 | 0.0021 | 0.0060 | 0.4863 | 0.5033 |
| 335 | Luizet | 3971a | Continental Europe | 0.0017 | 0.0017 | 0.0024 | 0.9916 | 0.0025 |
| 336 | Luizet | 3972a | Continental Europe | 0.0019 | 0.0018 | 0.0029 | 0.9908 | 0.0027 |
| 337 | Luizet | 7319a | Continental Europe | 0.0019 | 0.0018 | 0.0027 | 0.9909 | 0.0027 |
| 338 | Kesoi Rozsa | A0144 | Continental Europe | 0.2110 | 0.4890 | 0.0162 | 0.0260 | 0.2578 |
| 339 | Klosterneiburger | A12 | Continental Europe | 0.0022 | 0.3327 | 0.0033 | 0.6587 | 0.0031 |
| 340 | Cinska USL n°2 | A1345 | Continental Europe | 0.0164 | 0.9738 | 0.0039 | 0.0033 | 0.0026 |
| 341 | Cais Trandafiniu | A1663 | Continental Europe | 0.0038 | 0.0033 | 0.0101 | 0.3624 | 0.6203 |
| 342 | Uriase de Pecs | A1690 | Continental Europe | 0.0017 | 0.0017 | 0.0027 | 0.9911 | 0.0029 |
| 343 | Timpuri de Arad | A1700 | Continental Europe | 0.0251 | 0.0069 | 0.0056 | 0.0133 | 0.9490 |
| 344 | Dietrich A1 | A1800 | Continental Europe | 0.2885 | 0.6117 | 0.0782 | 0.0143 | 0.0072 |
| 345 | F1 Abricotier x amandier | A2036 | Interspecific hybrid *P. armeniaca* × *P. dulcis* (outgroup) | 0.0083 | 0.2077 | 0.0576 | 0.0058 | 0.7206 |
| 346 | Stean Rosie | A2147 | Continental Europe | 0.0042 | 0.0035 | 0.0106 | 0.4403 | 0.5414 |
| 347 | De Hollanda | A2339 | Continental Europe | 0.0023 | 0.0043 | 0.0323 | 0.0638 | 0.8973 |
| 348 | Timpurii de Kisinau | A2346 | Continental Europe | 0.0021 | 0.0143 | 0.0056 | 0.1991 | 0.7789 |
| 349 | Moorpark | A2635 | Continental Europe | 0.0026 | 0.0024 | 0.0039 | 0.0072 | 0.9839 |
| 350 | Karlotten | A2655 | Continental Europe | 0.5476 | 0.0029 | 0.0096 | 0.0029 | 0.4370 |
| 351 | Krupna Skopjanka | A2809 | Continental Europe | 0.0036 | 0.0031 | 0.0037 | 0.6470 | 0.3426 |
| 352 | Kec Psar | A3024 | Central Asia | 0.0055 | 0.9692 | 0.0162 | 0.0038 | 0.0053 |
| 353 | Beaugé | A601 | Continental Europe | 0.0018 | 0.0021 | 0.0066 | 0.0039 | 0.9857 |
| 354 | Kostinskij | A7 | European/Irano-Caucasian | 0.0023 | 0.7250 | 0.1163 | 0.1450 | 0.0114 |
| 355 | Rakovsheho | A755 | Continental Europe | 0.0023 | 0.0021 | 0.0038 | 0.4715 | 0.5203 |
| 356 | Madarska Narjlepsia | A882 | Continental Europe | 0.0017 | 0.0016 | 0.0028 | 0.9911 | 0.0028 |
| 357 | Keczke Mete Rosen (Kecskemeti Rozsa) | C11 | Continental Europe | 0.0028 | 0.4626 | 0.0068 | 0.2452 | 0.2826 |
| 358 | Krupnoplodá | C6 | Continental Europe | 0.0552 | 0.6564 | 0.0038 | 0.2655 | 0.0190 |
| 359 | Jubilejnyj | C9 | Continental Europe | 0.0021 | 0.3474 | 0.0035 | 0.6433 | 0.0037 |
| 360 | Chersonskij | D11 | Continental Europe | 0.0019 | 0.4140 | 0.0027 | 0.5789 | 0.0024 |
| 361 | Julskij | D3 | Continental Europe | 0.0082 | 0.8298 | 0.1081 | 0.0293 | 0.0245 |
| 362 | MoranD2 | MoranD2 | Continental Europe | 0.0018 | 0.0017 | 0.0025 | 0.9914 | 0.0026 |
| 363 | Morand N | MorN | Continental Europe | 0.0018 | 0.0017 | 0.0028 | 0.9905 | 0.0032 |
| 364 | Hungarian Best | Tk47 | Continental Europe | 0.0017 | 0.0016 | 0.0028 | 0.9911 | 0.0028 |
| 365 | Silistre de Rona | Tk61 | Continental Europe | 0.0110 | 0.2764 | 0.0454 | 0.3634 | 0.3039 |
| 366 | Cegled De Mamut (cegledi mamut) | U1611 | Continental Europe | 0.0065 | 0.0078 | 0.3367 | 0.4185 | 0.2306 |
| 367 | V/17 | U1638 | Continental Europe | 0.0053 | 0.0052 | 0.0109 | 0.6990 | 0.2795 |
| 368 | UK-1 | U2202 | Continental Europe | 0.0062 | 0.2388 | 0.2472 | 0.2870 | 0.2209 |
| 369 | Rakovsky BU 33 | U2345 | Continental Europe | 0.0028 | 0.0026 | 0.0049 | 0.5577 | 0.4321 |
| 370 | Cacak's gold | U2359 | Continental Europe | 0.0041 | 0.0036 | 0.0131 | 0.4275 | 0.5517 |
| 371 | Alberge de Tur | JM77 | Continental Europe | 0.0016 | 0.0016 | 0.0028 | 0.9911 | 0.0029 |
| 372 | Bergeron | JM78 | Continental Europe | 0.0022 | 0.5331 | 0.0131 | 0.4184 | 0.0331 |
| 373 | De Compot | JM79 | Continental Europe | 0.0020 | 0.3399 | 0.0089 | 0.5985 | 0.0507 |
| 374 | Jubileinyi | JM80 | Continental Europe | 0.0017 | 0.0017 | 0.0028 | 0.9911 | 0.0029 |
| 375 | Krasnoshchekii | JM81 | Continental Europe | 0.0017 | 0.0017 | 0.0028 | 0.9910 | 0.0029 |
| 376 | Luizet Krupnoplodnyi | JM82 | Continental Europe | 0.0028 | 0.0046 | 0.3304 | 0.3728 | 0.2895 |
| 377 | Precoce d'ltalia | JM83 | Continental Europe | 0.0033 | 0.0028 | 0.0046 | 0.0028 | 0.9865 |
| 378 | Real d'Imola | JM84 | Continental Europe | 0.0024 | 0.0046 | 0.0186 | 0.0044 | 0.9700 |
| 379 | Velkopavlovichka | JM85 | Continental Europe | 0.0029 | 0.0022 | 0.0029 | 0.0027 | 0.9893 |
| 380 | Vengerskii Krypnyi | JM86 | Continental Europe | 0.0030 | 0.0032 | 0.0349 | 0.3896 | 0.5694 |
| 381 | Vynoslivyi | JM87 | Continental Europe | 0.0111 | 0.9391 | 0.0294 | 0.0021 | 0.0182 |
| 382 | Currot | 25826 | Mediterranean Europe | 0.0043 | 0.0137 | 0.1005 | 0.4378 | 0.4437 |
| 383 | Saint-Jean de Bruel | 25827 | Mediterranean Europe | 0.0114 | 0.0054 | 0.7152 | 0.0084 | 0.2596 |
| 384 | Précoce de Boulbon | 25828 | Mediterranean Europe | 0.0022 | 0.0019 | 0.0031 | 0.4139 | 0.5789 |
| 385 | Gros Rouge | 25829 | Mediterranean Europe | 0.0022 | 0.0019 | 0.0031 | 0.4139 | 0.5789 |
| 386 | Fleurit Tard | 25830 | Mediterranean Europe | 0.0018 | 0.0023 | 0.0053 | 0.0119 | 0.9786 |
| 387 | Royal | 25831 | Mediterranean Europe | 0.0022 | 0.0025 | 0.0141 | 0.3474 | 0.6338 |
| 388 | Poizat | 25832 | Mediterranean Europe | 0.0020 | 0.0022 | 0.0119 | 0.3336 | 0.6503 |
| 389 | Merveilles du Dauphiné | 25833 | Mediterranean Europe | 0.7066 | 0.0058 | 0.0186 | 0.0039 | 0.2651 |
| 390 | Julin | 25834 | Mediterranean Europe | 0.0016 | 0.0017 | 0.0025 | 0.9918 | 0.0025 |
| 391 | Ampuis | 25835 | Mediterranean Europe | 0.0019 | 0.0022 | 0.0101 | 0.0037 | 0.9820 |
| 392 | Pêche de Nancy | 25837 | Mediterranean Europe | 0.7731 | 0.0029 | 0.0410 | 0.0153 | 0.1677 |
| 393 | Rouge de Mauves | 25838 | Mediterranean Europe | 0.0032 | 0.0133 | 0.0158 | 0.4085 | 0.5593 |
| 394 | Muscat | 25839 | Mediterranean Europe | 0.0023 | 0.0024 | 0.0037 | 0.0077 | 0.9840 |
| 395 | Manri | 25851 | Mediterranean Europe | 0.0021 | 0.0021 | 0.0062 | 0.0048 | 0.9849 |
| 396 | Corbato | 25852 | Mediterranean Europe | 0.0026 | 0.0054 | 0.0864 | 0.0028 | 0.9028 |
| 397 | Rojo de Carlet | 25853 | Mediterranean Europe | 0.0031 | 0.0027 | 0.0054 | 0.0085 | 0.9803 |
| 398 | Palabras | 25854 | Mediterranean Europe | 0.0092 | 0.0101 | 0.0149 | 0.0059 | 0.9599 |
| 399 | Palau | 25855 | Mediterranean Europe | 0.0021 | 0.0021 | 0.0062 | 0.0046 | 0.9850 |
| 400 | Ginesta | 25857 | Mediterranean Europe | 0.0019 | 0.0020 | 0.0059 | 0.0056 | 0.9846 |
| 401 | Martinet | 25858 | Mediterranean Europe | 0.0024 | 0.0026 | 0.0191 | 0.0069 | 0.9691 |
| 402 | Cristali | 25859 | Mediterranean Europe | 0.0043 | 0.0037 | 0.0154 | 0.0120 | 0.9645 |
| 403 | Chirivello | 25860 | Mediterranean Europe | 0.0028 | 0.0044 | 0.0677 | 0.0066 | 0.9185 |
| 404 | Gandia | 25861 | Mediterranean Europe | 0.0021 | 0.0021 | 0.0060 | 0.0047 | 0.9851 |
| 405 | Gabachet | 25862 | Mediterranean Europe | 0.0016 | 0.0023 | 0.0131 | 0.0111 | 0.9719 |
| 406 | Currot | 25863 | Mediterranean Europe | 0.0035 | 0.0029 | 0.0248 | 0.0205 | 0.9483 |
| 407 | Angliers | 25869 | Mediterranean Europe | 0.0080 | 0.0025 | 0.0286 | 0.0085 | 0.9523 |
| 408 | Abricot plat | 25870 | Mediterranean Europe | 0.0134 | 0.5923 | 0.1192 | 0.0450 | 0.2302 |
| 409 | Monaco Bello | 12PB390 | Mediterranean Europe | 0.0027 | 0.0025 | 0.0180 | 0.0040 | 0.9729 |
| 410 | Mandorla Dolce | 12PB391 | Mediterranean Europe | 0.0016 | 0.0017 | 0.0028 | 0.9911 | 0.0029 |
| 411 | Precoce d'Imola | 12PB392 | Mediterranean Europe | 0.5299 | 0.0030 | 0.0093 | 0.0032 | 0.4546 |
| 412 | Rapareddu | 12PB393 | Mediterranean Europe | 0.0022 | 0.0037 | 0.9672 | 0.0111 | 0.0157 |
| 413 | San Castrese | 12PB394 | Mediterranean Europe | 0.6273 | 0.0028 | 0.0163 | 0.0031 | 0.3504 |
| 414 | Boccacia spinosa | 12PB395 | Mediterranean Europe | 0.0028 | 0.0029 | 0.0941 | 0.0052 | 0.8951 |
| 415 | Menace | 12PB396 | Mediterranean Europe | 0.0021 | 0.0036 | 0.9076 | 0.0023 | 0.0844 |
| 416 | Dwarf Darnaud St Gervais | 12PB417 | Mediterranean Europe | 0.3800 | 0.0024 | 0.0036 | 0.0041 | 0.6099 |
| 417 | Ubones | 12PB592 | Mediterranean Europe | 0.0022 | 0.0019 | 0.0030 | 0.4136 | 0.5793 |
| 418 | Pepito grio | 12PB593 | Mediterranean Europe | 0.0633 | 0.0090 | 0.0156 | 0.0056 | 0.9065 |
| 419 | Juliette | 12PB594 | Mediterranean Europe | 0.0030 | 0.0026 | 0.0041 | 0.0047 | 0.9856 |
| 420 | Apr 25 PO | 12PB595 | Mediterranean Europe | 0.0066 | 0.0026 | 0.0284 | 0.0092 | 0.9533 |
| 421 | Apr 26 PO | 12PB596 | Mediterranean Europe | 0.0065 | 0.0033 | 0.0323 | 0.0421 | 0.9158 |
| 422 | Var Luventons | 12PB599 | Mediterranean Europe | 0.0026 | 0.0030 | 0.1272 | 0.0053 | 0.8620 |
| 423 | Morro de Bou | 12PB600 | Mediterranean Europe | 0.0052 | 0.0044 | 0.7412 | 0.0057 | 0.2434 |
| 424 | Bord Capona | 12PB601 | Mediterranean Europe | 0.0022 | 0.0019 | 0.0061 | 0.0075 | 0.9823 |
| 425 | Del Marge | 12PB602 | Mediterranean Europe | 0.0033 | 0.0030 | 0.7626 | 0.0341 | 0.1971 |
| 426 | Domas Blanc | 12PB603 | Mediterranean Europe | 0.0059 | 0.0049 | 0.4446 | 0.0047 | 0.5399 |
| 427 | Moscatell | 12PB604 | Mediterranean Europe | 0.0034 | 0.0031 | 0.7625 | 0.0340 | 0.1971 |
| 428 | Primarenc 4 | 12PB605 | Mediterranean Europe | 0.0020 | 0.0020 | 0.0067 | 0.0055 | 0.9838 |
| 429 | Galta 1 | 12PB606 | Mediterranean Europe | 0.0051 | 0.0039 | 0.8210 | 0.0164 | 0.1535 |
| 430 | Galta 2 | 12PB607 | Mediterranean Europe | 0.0052 | 0.0059 | 0.8980 | 0.0233 | 0.0676 |
| 431 | Galta 4 | 12PB608 | Mediterranean Europe | 0.0061 | 0.0050 | 0.7595 | 0.0129 | 0.2164 |
| 432 | Galta 5 | 12PB609 | Mediterranean Europe | 0.0051 | 0.0039 | 0.8581 | 0.0083 | 0.1246 |
| 433 | Primarenc 1 | 12PB610 | Mediterranean Europe | 0.0091 | 0.0066 | 0.2953 | 0.0211 | 0.6679 |
| 434 | Taronsal | 12PB611 | Mediterranean Europe | 0.0032 | 0.0198 | 0.4631 | 0.0075 | 0.5064 |
| 435 | Primarenc 3 | 12PB612 | Mediterranean Europe | 0.0042 | 0.0570 | 0.2851 | 0.0274 | 0.6263 |
| 436 | Murto 2 | 12PB613 | Mediterranean Europe | 0.0149 | 0.0419 | 0.0233 | 0.0039 | 0.9160 |
| 437 | Primarenc 2 | 12PB614 | Mediterranean Europe | 0.0021 | 0.0022 | 0.0066 | 0.0046 | 0.9845 |
| 438 | Galta 5 (frano) | 12PB615 | Mediterranean Europe | 0.0037 | 0.0042 | 0.9657 | 0.0089 | 0.0175 |
| 439 | Inquero | 12PB616 | Mediterranean Europe | 0.0131 | 0.0159 | 0.0054 | 0.0029 | 0.9627 |
| 440 | Galta 8 (R Brot curt) | 12PB617 | Mediterranean Europe | 0.0035 | 0.0048 | 0.9617 | 0.0087 | 0.0213 |
| 441 | Murto | 12PB619 | Mediterranean Europe | 0.0056 | 0.0031 | 0.0072 | 0.0035 | 0.9807 |
| 442 | Galta 6 (Brot curt) | 12PB620 | Mediterranean Europe | 0.0051 | 0.0044 | 0.7445 | 0.0057 | 0.2402 |
| 443 | Galta (Brot llarg) | 12PB621 | Mediterranean Europe | 0.0051 | 0.0044 | 0.7414 | 0.0059 | 0.2432 |
| 444 | Bulida | A0014 | Mediterranean Europe | 0.0017 | 0.0016 | 0.0025 | 0.9915 | 0.0026 |
| 445 | Ampuis | A0039 | Mediterranean Europe | 0.0042 | 0.1146 | 0.0173 | 0.3019 | 0.5621 |
| 446 | Pêche de Nancy | A0076 | Mediterranean Europe | 0.0024 | 0.0020 | 0.0084 | 0.4841 | 0.5032 |
| 447 | Hatifcolomer | A0082 | Mediterranean Europe | 0.9564 | 0.0026 | 0.0112 | 0.0092 | 0.0206 |
| 448 | kaiska | A0110 | Mediterranean Europe | 0.0038 | 0.0026 | 0.0326 | 0.1408 | 0.8201 |
| 449 | Desfargue n°1 | A0217 | Mediterranean Europe | 0.0022 | 0.1831 | 0.7865 | 0.0124 | 0.0158 |
| 450 | A384n voisin colomer | A0384 | Mediterranean Europe | 0.0025 | 0.0025 | 0.0442 | 0.0054 | 0.9455 |
| 451 | Blanc rosé | A0545 | Mediterranean Europe | 0.0019 | 0.0022 | 0.0040 | 0.2248 | 0.7672 |
| 452 | proche Bergeron | A0571 | Mediterranean Europe | 0.0019 | 0.0021 | 0.0053 | 0.3409 | 0.6498 |
| 453 | Paviot | A0654 | Mediterranean Europe | 0.0019 | 0.0023 | 0.0053 | 0.0117 | 0.9788 |
| 454 | Poizat 20 | A0657 | Mediterranean Europe | 0.0020 | 0.0023 | 0.0187 | 0.2962 | 0.6809 |
| 455 | Canino | A0924 | Mediterranean Europe | 0.0026 | 0.0028 | 0.0038 | 0.6634 | 0.3274 |
| 456 | Sucré de Holub | A10 | Mediterranean Europe | 0.0025 | 0.0021 | 0.0227 | 0.0036 | 0.9690 |
| 457 | Gabriel Bergeron | A1127 | Mediterranean Europe | 0.0024 | 0.0020 | 0.0087 | 0.5245 | 0.4624 |
| 458 | Bergeron | A114 | Mediterranean Europe | 0.0116 | 0.6104 | 0.2004 | 0.0354 | 0.1422 |
| 459 | Alberchigo de confitar | A1176 | Mediterranean Europe | 0.0025 | 0.0023 | 0.4774 | 0.0175 | 0.5004 |
| 460 | Manicot | A1236 | Mediterranean Europe | 0.0048 | 0.0336 | 0.0551 | 0.1892 | 0.7174 |
| 461 | Manicot pied-mere | A1236 | Mediterranean Europe | 0.0035 | 0.0024 | 0.0659 | 0.2212 | 0.7070 |
| 462 | Proche Perla | A1311 | Mediterranean Europe | 0.0031 | 0.0027 | 0.0065 | 0.0048 | 0.9829 |
| 463 | Arrogante | A1314 | Mediterranean Europe | 0.0050 | 0.9878 | 0.0021 | 0.0017 | 0.0033 |
| 464 | Gitano | A1317 | Mediterranean Europe | 0.0024 | 0.0140 | 0.0335 | 0.0042 | 0.9459 |
| 465 | Pepito | A1319 | Mediterranean Europe | 0.0030 | 0.0032 | 0.0067 | 0.0031 | 0.9840 |
| 466 | Polonais | A1352 | Mediterranean Europe | 0.0063 | 0.0030 | 0.0085 | 0.0029 | 0.9793 |
| 467 | Polonais n°2- Polonais Rebeyrolle | A1354 | Mediterranean Europe | 0.0025 | 0.0030 | 0.0384 | 0.0050 | 0.9511 |
| 468 | Palummella | A1356 | Mediterranean Europe | 0.7363 | 0.0088 | 0.0149 | 0.0025 | 0.2375 |
| 469 | Rouge du Roussillon | A157 | Mediterranean Europe | 0.0068 | 0.0025 | 0.0257 | 0.0089 | 0.9561 |
| 470 | San Fernando Biecheler | A1596 | Mediterranean Europe | 0.0057 | 0.0539 | 0.0606 | 0.0176 | 0.8622 |
| 471 | Canino | A1600 | Mediterranean Europe | 0.9559 | 0.0025 | 0.0116 | 0.0092 | 0.0208 |
| 472 | Bulida | A1601 | Mediterranean Europe | 0.0041 | 0.0027 | 0.0180 | 0.0088 | 0.9664 |
| 473 | Jaubert Foulon | A1602 | Mediterranean Europe | 0.7079 | 0.0086 | 0.0189 | 0.0030 | 0.2616 |
| 474 | RDR 377 | A1666 | Mediterranean Europe | 0.0067 | 0.0025 | 0.0257 | 0.0090 | 0.9561 |
| 475 | Précoce de Boulbon | A1685 | Mediterranean Europe | 0.0020 | 0.0024 | 0.0041 | 0.2336 | 0.7580 |
| 476 | Dr Mascle | A1686 | Mediterranean Europe | 0.8484 | 0.0025 | 0.0136 | 0.0089 | 0.1266 |
| 477 | Super Rouge | A1710 | Mediterranean Europe | 0.0430 | 0.0056 | 0.2175 | 0.0485 | 0.6854 |
| 478 | Avikaline | A1711 | Mediterranean Europe | 0.0026 | 0.0021 | 0.0089 | 0.6447 | 0.3418 |
| 479 | Colomer Arbre2 | A1712 | Mediterranean Europe | 0.9607 | 0.0026 | 0.0063 | 0.0055 | 0.0249 |
| 480 | Rouge de Sernhac | A1714 | Mediterranean Europe | 0.0021 | 0.0042 | 0.0837 | 0.0243 | 0.8856 |
| 481 | Précoce de Tyrinthe | A1721 | Mediterranean Europe | 0.0017 | 0.0017 | 0.0040 | 0.9883 | 0.0044 |
| 482 | ABR 882 | A1726 | Mediterranean Europe | 0.9565 | 0.0025 | 0.0114 | 0.0091 | 0.0205 |
| 483 | Prete | A1744 | Mediterranean Europe | 0.0027 | 0.0024 | 0.0151 | 0.0037 | 0.9761 |
| 484 | Albi Precocci n°1 | A1745 | Mediterranean Europe | 0.0030 | 0.0091 | 0.0300 | 0.0087 | 0.9492 |
| 485 | Donato | A1748 | Mediterranean Europe | 0.0055 | 0.0326 | 0.2287 | 0.0100 | 0.7232 |
| 486 | Boccuccia Eboli | A1750 | Mediterranean Europe | 0.0049 | 0.0026 | 0.0359 | 0.0079 | 0.9488 |
| 487 | Tardif de Bordaneil 2 | A1760 | Mediterranean Europe | 0.0031 | 0.0027 | 0.1439 | 0.2003 | 0.6500 |
| 488 | Houcall | A1792 | Mediterranean Europe | 0.8780 | 0.0021 | 0.0077 | 0.0108 | 0.1014 |
| 489 | Tardif de Bordaneil 1 | A1793 | Mediterranean Europe | 0.0031 | 0.0027 | 0.1511 | 0.1727 | 0.6703 |
| 490 | SH50 | A1801 | Mediterranean Europe | 0.6590 | 0.0029 | 0.0082 | 0.0318 | 0.2981 |
| 491 | SH7 | A1802 | Mediterranean Europe | 0.1092 | 0.0118 | 0.0518 | 0.0063 | 0.8209 |
| 492 | Précoce de Tyrinthe | A1809 | Mediterranean Europe | 0.5282 | 0.0028 | 0.0068 | 0.0067 | 0.4555 |
| 493 | San Castrese | A1915 | Mediterranean Europe | 0.0024 | 0.0028 | 0.0254 | 0.0048 | 0.9646 |
| 494 | Pazza | A1916 | Mediterranean Europe | 0.3289 | 0.3078 | 0.0932 | 0.0042 | 0.2660 |
| 495 | Portici 2 | A1925 | Mediterranean Europe | 0.0100 | 0.3279 | 0.2972 | 0.2004 | 0.1646 |
| 496 | Fantasme | A1939 | Mediterranean Europe | 0.0027 | 0.0159 | 0.0263 | 0.4907 | 0.4643 |
| 497 | Susincocco | A1940 | Mediterranean Europe | 0.0030 | 0.0032 | 0.0068 | 0.0030 | 0.9840 |
| 498 | Bebeco n°15 | A2087 | Mediterranean Europe | 0.0025 | 0.0019 | 0.0071 | 0.0070 | 0.9815 |
| 499 | Bebeco n°16 | A2088 | Mediterranean Europe | 0.0031 | 0.0229 | 0.6409 | 0.0392 | 0.2939 |
| 500 | Bebeco | A2089 | Mediterranean Europe | 0.0029 | 0.0025 | 0.0172 | 0.0039 | 0.9736 |
| 501 | Bebeco n°18 | A2090 | Mediterranean Europe | 0.0029 | 0.0020 | 0.0073 | 0.0041 | 0.9837 |
| 502 | Nancy | A2108 | Mediterranean Europe | 0.0025 | 0.0030 | 0.0397 | 0.0050 | 0.9498 |
| 503 | Rouge de Fournes | A2129 | Mediterranean Europe | 0.7265 | 0.0029 | 0.0253 | 0.0037 | 0.2416 |
| 504 | Carrascal | A2131 | Mediterranean Europe | 0.0072 | 0.7159 | 0.1641 | 0.0300 | 0.0828 |
| 505 | Bebeco La2A | A2187 | Mediterranean Europe | 0.0030 | 0.0020 | 0.0054 | 0.0050 | 0.9847 |
| 506 | Bebeco | A2204 | Mediterranean Europe | 0.0030 | 0.0025 | 0.0113 | 0.0048 | 0.9785 |
| 507 | Précoce du Portugal | A2313 | Mediterranean Europe | 0.8732 | 0.0034 | 0.0518 | 0.0359 | 0.0357 |
| 508 | Docteur Mascle | A2314 | Mediterranean Europe | 0.8729 | 0.0034 | 0.0521 | 0.0357 | 0.0359 |
| 509 | Taddeo | A2319 | Mediterranean Europe | 0.0049 | 0.0031 | 0.0067 | 0.0036 | 0.9817 |
| 510 | Monaco Bello | A2330 | Mediterranean Europe | 0.6103 | 0.0034 | 0.0099 | 0.0050 | 0.3714 |
| 511 | Précoce Pugget | A2351 | Mediterranean Europe | 0.9805 | 0.0024 | 0.0044 | 0.0049 | 0.0078 |
| 512 | Precoce Firenze | A2352 | Mediterranean Europe | 0.0032 | 0.0509 | 0.2398 | 0.1116 | 0.5946 |
| 513 | Amabile Vecchioni | A2353 | Mediterranean Europe | 0.5822 | 0.0031 | 0.0135 | 0.0031 | 0.3980 |
| 514 | Sernsniy | A2354 | Mediterranean Europe | 0.5822 | 0.0031 | 0.0144 | 0.0031 | 0.3972 |
| 515 | Bruel | A2645 | Mediterranean Europe | 0.8730 | 0.0034 | 0.0532 | 0.0350 | 0.0354 |
| 516 | Portici | A2653 | Mediterranean Europe | 0.0027 | 0.0029 | 0.1216 | 0.0056 | 0.8673 |
| 517 | Viceroy | A2654 | Mediterranean Europe | 0.0074 | 0.4560 | 0.1539 | 0.0046 | 0.3782 |
| 518 | Vitillo | A2657 | Mediterranean Europe | 0.4713 | 0.0025 | 0.0148 | 0.0026 | 0.5087 |
| 519 | Cruzman | A2719 | Mediterranean Europe | 0.4317 | 0.0022 | 0.0033 | 0.0051 | 0.5577 |
| 520 | Pancin | A2720 | Mediterranean Europe | 0.0021 | 0.0019 | 0.0030 | 0.4139 | 0.5790 |
| 521 | Bulida | A2922 | Mediterranean Europe | 0.0045 | 0.0028 | 0.0134 | 0.0095 | 0.9698 |
| 522 | Ajouc | A3408 | Mediterranean Europe | 0.0037 | 0.1979 | 0.0068 | 0.1833 | 0.6082 |
| 523 | Rouge de Rivesaltes 2 | A380 | Mediterranean Europe | 0.0058 | 0.4187 | 0.5484 | 0.0051 | 0.0220 |
| 524 | Royal | A3837 | Mediterranean Europe | 0.0020 | 0.0030 | 0.0151 | 0.0081 | 0.9718 |
| 525 | Précoce Ampuis | A39 | Mediterranean Europe | 0.1949 | 0.2043 | 0.4777 | 0.0071 | 0.1159 |
| 526 | Precoce d'Italie | A526 | Mediterranean Europe | 0.0021 | 0.0022 | 0.0060 | 0.0034 | 0.9863 |
| 527 | Poizat | A539 | Mediterranean Europe | 0.0021 | 0.0022 | 0.0060 | 0.0035 | 0.9863 |
| 528 | Ampuis | A5403 | Mediterranean Europe | 0.0020 | 0.0028 | 0.0108 | 0.0039 | 0.9805 |
| 529 | Delpierre | A5405 | Mediterranean Europe | 0.8886 | 0.0025 | 0.0138 | 0.0076 | 0.0876 |
| 530 | Delpierre Précoce | A5406 | Mediterranean Europe | 0.9583 | 0.0035 | 0.0109 | 0.0081 | 0.0192 |
| 531 | Delpierre Précoce2 | A5406 | Mediterranean Europe | 0.0023 | 0.0022 | 0.0039 | 0.0049 | 0.9867 |
| 532 | Mathieu Paret | A5407 | Mediterranean Europe | 0.0020 | 0.0020 | 0.0047 | 0.0247 | 0.9666 |
| 533 | Merveille Delpierre | A5408 | Mediterranean Europe | 0.0020 | 0.0022 | 0.0114 | 0.3340 | 0.6505 |
| 534 | Muscat Pêches Nancy | A5409 | Mediterranean Europe | 0.0028 | 0.0038 | 0.0050 | 0.7285 | 0.2599 |
| 535 | Paviot | A5410 | Mediterranean Europe | 0.0074 | 0.0135 | 0.0084 | 0.0071 | 0.9636 |
| 536 | Poman Rosé | A5412 | Mediterranean Europe | 0.0031 | 0.0025 | 0.0239 | 0.1680 | 0.8025 |
| 537 | Précoce de Boulbon | A5414 | Mediterranean Europe | 0.0071 | 0.0139 | 0.0046 | 0.2275 | 0.7468 |
| 538 | Dr Mascle Prospect | A5415 | Mediterranean Europe | 0.7666 | 0.0034 | 0.0528 | 0.0167 | 0.1605 |
| 539 | Précoce Saint Jean de Bruel | A5417 | Mediterranean Europe | 0.0028 | 0.0190 | 0.0558 | 0.0929 | 0.8295 |
| 540 | Pointu de Roqueverre | A5418 | Mediterranean Europe | 0.0025 | 0.0022 | 0.0136 | 0.5474 | 0.4343 |
| 541 | Apr 25 | A5419 | Mediterranean Europe | 0.0068 | 0.0025 | 0.0252 | 0.0088 | 0.9568 |
| 542 | Apr 26 | A5420 | Mediterranean Europe | 0.0071 | 0.0032 | 0.0344 | 0.0408 | 0.9144 |
| 543 | Cafona | A544 | Mediterranean Europe | 0.0021 | 0.0028 | 0.1115 | 0.0076 | 0.8761 |
| 544 | Nanassa | A547 | Mediterranean Europe | 0.0031 | 0.0027 | 0.1502 | 0.1736 | 0.6704 |
| 545 | Bergeron | A660 | Mediterranean Europe | 0.0024 | 0.0020 | 0.0089 | 0.5242 | 0.4625 |
| 546 | Royer | A664 | Mediterranean Europe | 0.0023 | 0.0020 | 0.0086 | 0.5243 | 0.4628 |
| 547 | Luizet | A665 | Mediterranean Europe | 0.0016 | 0.0017 | 0.0028 | 0.9911 | 0.0028 |
| 548 | Mandorlon | A692 | Mediterranean Europe | 0.0060 | 0.6735 | 0.0077 | 0.0133 | 0.2996 |
| 549 | Jaubert Foulon | A74 | Mediterranean Europe | 0.8819 | 0.0042 | 0.0060 | 0.0045 | 0.1034 |
| 550 | Colomer | A8 | Mediterranean Europe | 0.9717 | 0.0024 | 0.0081 | 0.0064 | 0.0113 |
| 551 | Screara | A804 | Mediterranean Europe | 0.0026 | 0.0020 | 0.0058 | 0.0105 | 0.9791 |
| 552 | Reale d' Imola | A873 | Mediterranean Europe | 0.0021 | 0.0022 | 0.0059 | 0.0034 | 0.9864 |
| 553 | Realfino | Es1 | Mediterranean Europe | 0.0037 | 0.0045 | 0.0705 | 0.0036 | 0.9177 |
| 554 | Mauricio | Es2 | Mediterranean Europe | 0.0026 | 0.0020 | 0.0200 | 0.0080 | 0.9673 |
| 555 | Currot | Es3 | Mediterranean Europe | 0.0035 | 0.0028 | 0.0221 | 0.0203 | 0.9514 |
| 556 | Bulida | Es4 | Mediterranean Europe | 0.0040 | 0.0027 | 0.0182 | 0.0090 | 0.9662 |
| 557 | Velazquez | Es5 | Mediterranean Europe | 0.0020 | 0.0024 | 0.0336 | 0.0043 | 0.9578 |
| 558 | Canino | Es6 | Mediterranean Europe | 0.0022 | 0.0019 | 0.0064 | 0.0074 | 0.9821 |
| 559 | Moniqui | Es7 | Mediterranean Europe | 0.0029 | 0.0043 | 0.0142 | 0.0029 | 0.9756 |
| 560 | Pepito del Rubio | Es8 | Mediterranean Europe | 0.0029 | 0.0025 | 0.0039 | 0.0048 | 0.9859 |
| 561 | Acqua del Serino | It01 | Mediterranean Europe | 0.0036 | 0.0054 | 0.0238 | 0.0117 | 0.9555 |
| 562 | Alessandrino | It02 | Mediterranean Europe | 0.0016 | 0.0019 | 0.0050 | 0.0044 | 0.9871 |
| 563 | Amabile Vecchioni | It03 | Mediterranean Europe | 0.5627 | 0.0032 | 0.0143 | 0.0033 | 0.4165 |
| 564 | Baracca | It04 | Mediterranean Europe | 0.0034 | 0.0022 | 0.2060 | 0.0033 | 0.7850 |
| 565 | Bella d'Imola | It05 | Mediterranean Europe | 0.5711 | 0.0027 | 0.0033 | 0.0022 | 0.4207 |
| 566 | Bella si San Giuliano | It06 | Mediterranean Europe | 0.0508 | 0.0252 | 0.0079 | 0.0120 | 0.9041 |
| 567 | Boccuccia | It07 | Mediterranean Europe | 0.8120 | 0.0036 | 0.0332 | 0.0023 | 0.1489 |
| 568 | Boccuccia Liscia | It08 | Mediterranean Europe | 0.0032 | 0.0025 | 0.0343 | 0.0041 | 0.9559 |
| 569 | Boccuccia Spinosa | It09 | Mediterranean Europe | 0.0026 | 0.0027 | 0.1701 | 0.0065 | 0.8180 |
| 570 | Breda (di) | It10 | Mediterranean Europe | 0.0016 | 0.0017 | 0.0027 | 0.9911 | 0.0029 |
| 571 | Cafona MIPAF | It11 | Mediterranean Europe | 0.0021 | 0.0027 | 0.0903 | 0.0064 | 0.8985 |
| 572 | Caldesi 1 | It12 | Mediterranean Europe | 0.3493 | 0.0023 | 0.0034 | 0.0024 | 0.6426 |
| 573 | Caldesi 2 | It13 | Mediterranean Europe | 0.3703 | 0.0023 | 0.0049 | 0.0032 | 0.6194 |
| 574 | Ceccona | It14 | Mediterranean Europe | 0.5828 | 0.0028 | 0.0066 | 0.0035 | 0.4043 |
| 575 | Certosa A 5 | It15 | Mediterranean Europe | 0.0021 | 0.0019 | 0.0031 | 0.4142 | 0.5787 |
| 576 | Certosa A 8 | It16 | Mediterranean Europe | 0.0022 | 0.0019 | 0.0031 | 0.4530 | 0.5397 |
| 577 | Certosa B 5 | It17 | Mediterranean Europe | 0.0021 | 0.0019 | 0.0030 | 0.4140 | 0.5789 |
| 578 | Cibo del Paradiso | It18 | Mediterranean Europe | 0.0019 | 0.0020 | 0.0144 | 0.0050 | 0.9767 |
| 579 | Comune | It19 | Mediterranean Europe | 0.0036 | 0.0113 | 0.1089 | 0.1669 | 0.7095 |
| 580 | Corona Quartucciu | It20 | Mediterranean Europe | 0.0029 | 0.0093 | 0.2018 | 0.0402 | 0.7458 |
| 581 | Mandorlon | It21 | Mediterranean Europe | 0.5713 | 0.0026 | 0.0034 | 0.0022 | 0.4205 |
| 582 | Costa Sciacca Ragana 2 | It22 | Mediterranean Europe | 0.5065 | 0.0531 | 0.0107 | 0.0025 | 0.4272 |
| 583 | Crisommene | It23 | Mediterranean Europe | 0.0071 | 0.0033 | 0.0738 | 0.0032 | 0.9126 |
| 584 | D' Alessandria | It24 | Mediterranean Europe | 0.0021 | 0.0022 | 0.0056 | 0.0031 | 0.9870 |
| 585 | Dasycarpa PI 1 / 1 | It25 | Mediterranean Europe | 0.0464 | 0.5730 | 0.0073 | 0.3666 | 0.0067 |
| 586 | Grossa del Giardino | It26 | Mediterranean Europe | 0.0021 | 0.0022 | 0.0056 | 0.0031 | 0.9870 |
| 587 | Maiolino di Menfi | It27 | Mediterranean Europe | 0.0030 | 0.0025 | 0.0109 | 0.0049 | 0.9787 |
| 588 | Mandorla Dolce | It28 | Mediterranean Europe | 0.0017 | 0.0016 | 0.0028 | 0.9911 | 0.0029 |
| 589 | Menace | It29 | Mediterranean Europe | 0.0020 | 0.0020 | 0.9196 | 0.0022 | 0.0742 |
| 590 | Monaco | It30 | Mediterranean Europe | 0.0027 | 0.0024 | 0.0154 | 0.0036 | 0.9758 |
| 591 | Monaco bello | It31 | Mediterranean Europe | 0.0028 | 0.0024 | 0.0155 | 0.0036 | 0.9757 |
| 592 | Monti Ladak 16 | It32 | Mediterranean Europe | 0.0087 | 0.7620 | 0.0423 | 0.1523 | 0.0346 |
| 593 | Monti Ladak 17 | It33 | Mediterranean Europe | 0.0031 | 0.9683 | 0.0138 | 0.0097 | 0.0052 |
| 594 | O' Pazzo | It34 | Mediterranean Europe | 0.0024 | 0.0027 | 0.0255 | 0.0048 | 0.9647 |
| 595 | Orru Quartu | It35 | Mediterranean Europe | 0.0062 | 0.0097 | 0.0035 | 0.0031 | 0.9774 |
| 596 | Nonno | It36 | Mediterranean Europe | 0.0027 | 0.0029 | 0.0247 | 0.0045 | 0.9652 |
| 597 | Particolare | It37 | Mediterranean Europe | 0.0021 | 0.0022 | 0.0056 | 0.0031 | 0.9870 |
| 598 | Pelese di Giovanniello | It38 | Mediterranean Europe | 0.0028 | 0.0023 | 0.0133 | 0.0063 | 0.9754 |
| 599 | Pelluzzella | It39 | Mediterranean Europe | 0.0052 | 0.0089 | 0.0931 | 0.0073 | 0.8855 |
| 600 | Persicini piccola | It40 | Mediterranean Europe | 0.0036 | 0.0179 | 0.2138 | 0.0090 | 0.7557 |
| 601 | Del Pittore | It41 | Mediterranean Europe | 0.4687 | 0.0760 | 0.0127 | 0.0055 | 0.4371 |
| 602 | Portici | It42 | Mediterranean Europe | 0.0027 | 0.0029 | 0.1214 | 0.0055 | 0.8676 |
| 603 | Portici 2 | It43 | Mediterranean Europe | 0.0027 | 0.0029 | 0.1184 | 0.0056 | 0.8704 |
| 604 | Precoce d' Imola | It44 | Mediterranean Europe | 0.5629 | 0.0032 | 0.0138 | 0.0032 | 0.4169 |
| 605 | Precoce d'Italia | It45 | Mediterranean Europe | 0.0050 | 0.1240 | 0.8431 | 0.0056 | 0.0223 |
| 606 | Precoce di Sicilia | It46 | Mediterranean Europe | 0.0025 | 0.0825 | 0.1222 | 0.0060 | 0.7868 |
| 607 | Rapareddo | It47 | Mediterranean Europe | 0.0031 | 0.0056 | 0.9620 | 0.0115 | 0.0177 |
| 608 | Reale d' Imola | It48 | Mediterranean Europe | 0.0022 | 0.0246 | 0.0060 | 0.0030 | 0.9642 |
| 609 | San Castrese | It49 | Mediterranean Europe | 0.7229 | 0.0029 | 0.0150 | 0.0026 | 0.2568 |
| 610 | San Francesco | It50 | Mediterranean Europe | 0.0025 | 0.0025 | 0.5007 | 0.0054 | 0.4890 |
| 611 | Sant' Ambrogio | It51 | Mediterranean Europe | 0.9635 | 0.0026 | 0.0106 | 0.0082 | 0.0152 |
| 612 | Saritzu I | It52 | Mediterranean Europe | 0.0020 | 0.0024 | 0.4655 | 0.0045 | 0.5256 |
| 613 | Saritzu II | It53 | Mediterranean Europe | 0.0028 | 0.0092 | 0.2026 | 0.0400 | 0.7454 |
| 614 | Tardiva di Menfi | It54 | Mediterranean Europe | 0.0033 | 0.0695 | 0.0463 | 0.0096 | 0.8713 |
| 615 | Tiltonno | It55 | Mediterranean Europe | 0.0059 | 0.0049 | 0.2288 | 0.0087 | 0.7517 |
| 616 | Ungherese gialla | It56 | Mediterranean Europe | 0.0019 | 0.0022 | 0.0243 | 0.0032 | 0.9683 |
| 617 | Ungherese piccola | It57 | Mediterranean Europe | 0.0020 | 0.0021 | 0.0033 | 0.0043 | 0.9883 |
| 618 | Valleggia | It58 | Mediterranean Europe | 0.0025 | 0.0045 | 0.0196 | 0.0045 | 0.9688 |
| 619 | Vitillo | It60 | Mediterranean Europe | 0.4985 | 0.0027 | 0.0162 | 0.0024 | 0.4802 |
| 620 | Bebeco | Tk40 | Mediterranean Europe | 0.0030 | 0.0025 | 0.0110 | 0.0049 | 0.9786 |
| 621 | Canino | Tk42 | Mediterranean Europe | 0.0024 | 0.0049 | 0.2682 | 0.2956 | 0.4290 |
| 622 | Fracasso | Tk44 | Mediterranean Europe | 0.7415 | 0.0028 | 0.0144 | 0.0025 | 0.2389 |
| 623 | Paviot | Tk51 | Mediterranean Europe | 0.0021 | 0.0019 | 0.0030 | 0.4135 | 0.5794 |
| 624 | Polenais | Tk53 | Mediterranean Europe | 0.0031 | 0.0165 | 0.6977 | 0.0055 | 0.2772 |
| 625 | Precoce de Boulbon | Tk54 | Mediterranean Europe | 0.0023 | 0.0029 | 0.9837 | 0.0063 | 0.0048 |
| 626 | Precoce de Thyrinte | Tk55 | Mediterranean Europe | 0.5053 | 0.0028 | 0.0064 | 0.0049 | 0.4806 |
| 627 | Precoce de Colomer | Tk56 | Mediterranean Europe | 0.9698 | 0.0025 | 0.0088 | 0.0071 | 0.0119 |
| 628 | Royal | Tk59 | Mediterranean Europe | 0.0024 | 0.0048 | 0.2675 | 0.2944 | 0.4309 |
| 629 | Sancastrese | Tk60 | Mediterranean Europe | 0.9695 | 0.0025 | 0.0090 | 0.0070 | 0.0120 |
| 630 | Vitillo | Tk63 | Mediterranean Europe | 0.2713 | 0.1487 | 0.0133 | 0.0040 | 0.5628 |
| 631 | Alberge De Montgamet | U0945 | Mediterranean Europe | 0.0018 | 0.0018 | 0.0027 | 0.9910 | 0.0027 |
| 632 | Bou Herra | 3 | North-Africa | 0.0018 | 0.0026 | 0.7722 | 0.0196 | 0.2039 |
| 633 | Chéchi Dhraa Tammar | 9 | North-Africa | 0.0035 | 0.0047 | 0.0236 | 0.0269 | 0.9413 |
| 634 | Addadi Ahmar | 15 | North-Africa | 0.0020 | 0.0023 | 0.4778 | 0.0325 | 0.4854 |
| 635 | Om Younes | 16 | North-Africa | 0.0026 | 0.0193 | 0.7308 | 0.0239 | 0.2234 |
| 636 | Oud Gnaa | 27 | North-Africa | 0.0023 | 0.0040 | 0.8639 | 0.0327 | 0.0971 |
| 637 | Chéchi Horr | 29 | North-Africa | 0.0034 | 0.0047 | 0.0240 | 0.0266 | 0.9412 |
| 638 | Bangui | 31 | North-Africa | 0.0018 | 0.0026 | 0.7660 | 0.0194 | 0.2102 |
| 639 | Variété de Mahdia | 47 | North-Africa | 0.0018 | 0.0021 | 0.0125 | 0.0105 | 0.9731 |
| 640 | Jerba 67 | 67 | North-Africa | 0.0034 | 0.0033 | 0.7490 | 0.0298 | 0.2146 |
| 641 | Jerba 68 | 68 | North-Africa | 0.0021 | 0.0030 | 0.9795 | 0.0068 | 0.0088 |
| 642 | Jerba 69 | 69 | North-Africa | 0.0025 | 0.0028 | 0.9747 | 0.0133 | 0.0068 |
| 643 | Oud El Haj Tahar | 70 | North-Africa | 0.0049 | 0.0407 | 0.8044 | 0.0094 | 0.1405 |
| 644 | Oud Aouicha | 71 | North-Africa | 0.0021 | 0.0037 | 0.4661 | 0.0094 | 0.5187 |
| 645 | Zalouzi | 72 | North-Africa | 0.0028 | 0.0030 | 0.8035 | 0.0061 | 0.1845 |
| 646 | Chechi | 25845 | North-Africa | 0.0018 | 0.0021 | 0.0139 | 0.0107 | 0.9715 |
| 647 | Bayoudhi | 25846 | North-Africa | 0.0022 | 0.0023 | 0.0064 | 0.0050 | 0.9841 |
| 648 | Chéchi Khit El Oued | 10A | North-Africa | 0.0031 | 0.0112 | 0.0184 | 0.0064 | 0.9609 |
| 649 | Bayoudhi | 11B | North-Africa | 0.0026 | 0.0130 | 0.1254 | 0.0027 | 0.8563 |
| 650 | H'midi | 12C | North-Africa | 0.0020 | 0.0023 | 0.4774 | 0.0327 | 0.4855 |
| 651 | Bouk Hmed | 13A | North-Africa | 0.0017 | 0.0045 | 0.8522 | 0.0774 | 0.0642 |
| 652 | Faggoussi | 14C | North-Africa | 0.0032 | 0.0100 | 0.5409 | 0.0075 | 0.4383 |
| 653 | Aranji | 17A | North-Africa | 0.0022 | 0.0026 | 0.9735 | 0.0111 | 0.0105 |
| 654 | Oud Rhayem | 18A | North-Africa | 0.0021 | 0.0023 | 0.2500 | 0.0069 | 0.7387 |
| 655 | Bedri Ahmar | 19A | North-Africa | 0.0028 | 0.0316 | 0.1009 | 0.0843 | 0.7804 |
| 656 | Baccour | 1C | North-Africa | 0.0018 | 0.0022 | 0.9376 | 0.0431 | 0.0153 |
| 657 | Bedri | 1G | North-Africa | 0.0025 | 0.0025 | 0.6200 | 0.0298 | 0.3451 |
| 658 | Bouthani | 20C | North-Africa | 0.0019 | 0.0023 | 0.9621 | 0.0064 | 0.0273 |
| 659 | Oud Hmida | 21A | North-Africa | 0.0023 | 0.0037 | 0.9729 | 0.0072 | 0.0139 |
| 660 | Oud Tijani | 22B | North-Africa | 0.0026 | 0.0046 | 0.4213 | 0.0312 | 0.5403 |
| 661 | Oud Nakhla | 23B | North-Africa | 0.0023 | 0.0040 | 0.8615 | 0.0334 | 0.0988 |
| 662 | Oud Salah Ben Salem | 25B | North-Africa | 0.0028 | 0.0040 | 0.9387 | 0.0048 | 0.0496 |
| 663 | Chechi Bazza | 28D | North-Africa | 0.0044 | 0.0039 | 0.5711 | 0.0120 | 0.4086 |
| 664 | Khad Hlima | 2C | North-Africa | 0.0021 | 0.0021 | 0.0061 | 0.0046 | 0.9850 |
| 665 | Bouk Hmed Akhal | 34B | North-Africa | 0.0023 | 0.0030 | 0.8788 | 0.0265 | 0.0894 |
| 666 | Fourati | 38B | North-Africa | 0.0036 | 0.0022 | 0.0333 | 0.0046 | 0.9564 |
| 667 | Bargoug 40A | 40A | North-Africa | 0.0033 | 0.0054 | 0.9306 | 0.0235 | 0.0372 |
| 668 | Bargoug 40B | 40B | North-Africa | 0.0227 | 0.0027 | 0.9588 | 0.0054 | 0.0104 |
| 669 | Bargoug 40E | 40E | North-Africa | 0.0018 | 0.0028 | 0.9639 | 0.0051 | 0.0264 |
| 670 | Bargoug 40G | 40G | North-Africa | 0.0022 | 0.0055 | 0.9738 | 0.0110 | 0.0075 |
| 671 | Bargoug 40H | 40H | North-Africa | 0.0084 | 0.0030 | 0.9747 | 0.0028 | 0.0111 |
| 672 | Bargoug 40I | 40I | North-Africa | 0.0038 | 0.0058 | 0.8552 | 0.0028 | 0.1324 |
| 673 | Bargoug 40J | 40J | North-Africa | 0.0201 | 0.0036 | 0.9678 | 0.0036 | 0.0049 |
| 674 | Bargoug 40K | 40K | North-Africa | 0.0020 | 0.0023 | 0.9682 | 0.0190 | 0.0085 |
| 675 | Bargoug 40M | 40M | North-Africa | 0.0032 | 0.0060 | 0.8088 | 0.0033 | 0.1788 |
| 676 | Bargoug 40N | 40N | North-Africa | 0.0054 | 0.0214 | 0.7911 | 0.0132 | 0.1690 |
| 677 | Agrégé de Baccour | 41A | North-Africa | 0.0018 | 0.0022 | 0.9417 | 0.0402 | 0.0141 |
| 678 | Bargoug 42A | 42A | North-Africa | 0.0031 | 0.0023 | 0.9786 | 0.0072 | 0.0089 |
| 679 | Bargoug42B | 42B | North-Africa | 0.0025 | 0.0024 | 0.9877 | 0.0033 | 0.0041 |
| 680 | Bargoug 42C | 42C | North-Africa | 0.0039 | 0.0045 | 0.9814 | 0.0046 | 0.0056 |
| 681 | Bargoug 42G | 42G | North-Africa | 0.0029 | 0.0137 | 0.9752 | 0.0035 | 0.0047 |
| 682 | Bargoug 42H | 42H | North-Africa | 0.0031 | 0.0022 | 0.9786 | 0.0072 | 0.0088 |
| 683 | Bargoug 43B | 43B | North-Africa | 0.0132 | 0.1356 | 0.8372 | 0.0039 | 0.0100 |
| 684 | Bargoug 43C | 43C | North-Africa | 0.0027 | 0.0380 | 0.9460 | 0.0056 | 0.0077 |
| 685 | Bargoug 43D | 43D | North-Africa | 0.0037 | 0.0023 | 0.9862 | 0.0027 | 0.0050 |
| 686 | Bargoug 43F | 43F | North-Africa | 0.0063 | 0.0038 | 0.9699 | 0.0120 | 0.0080 |
| 687 | Bargoug 44A | 44A | North-Africa | 0.0129 | 0.0065 | 0.9275 | 0.0375 | 0.0157 |
| 688 | Bargoug 44B | 44B | North-Africa | 0.0040 | 0.0022 | 0.9822 | 0.0051 | 0.0064 |
| 689 | Bargoug 44C | 44C | North-Africa | 0.0083 | 0.0025 | 0.9764 | 0.0062 | 0.0065 |
| 690 | Bargoug 44D | 44D | North-Africa | 0.0025 | 0.0026 | 0.9535 | 0.0075 | 0.0339 |
| 691 | Bargoug 44E | 44E | North-Africa | 0.0123 | 0.0026 | 0.9733 | 0.0063 | 0.0056 |
| 692 | Bargoug 44F | 44F | North-Africa | 0.0028 | 0.0035 | 0.9877 | 0.0030 | 0.0030 |
| 693 | Bargoug 44G | 44G | North-Africa | 0.0039 | 0.0024 | 0.9854 | 0.0043 | 0.0041 |
| 694 | Bargoug 44H | 44H | North-Africa | 0.0037 | 0.0025 | 0.9723 | 0.0147 | 0.0068 |
| 695 | Bargoug 45B | 45B | North-Africa | 0.0034 | 0.0027 | 0.9819 | 0.0082 | 0.0039 |
| 696 | Bargoug 45C | 45C | North-Africa | 0.0024 | 0.0020 | 0.9819 | 0.0082 | 0.0056 |
| 697 | Bargoug 46B | 46B | North-Africa | 0.0018 | 0.0022 | 0.9749 | 0.0047 | 0.0163 |
| 698 | Bargoug 46C | 46C | North-Africa | 0.0040 | 0.0168 | 0.9686 | 0.0061 | 0.0045 |
| 699 | Bargoug 46D | 46D | North-Africa | 0.0040 | 0.0031 | 0.9802 | 0.0072 | 0.0055 |
| 700 | Bargoug 46E | 46E | North-Africa | 0.0029 | 0.0025 | 0.9860 | 0.0051 | 0.0036 |
| 701 | Bedri | 48A | North-Africa | 0.0030 | 0.0028 | 0.6581 | 0.0540 | 0.2821 |
| 702 | Bedri Thani | 48G | North-Africa | 0.0024 | 0.0027 | 0.5469 | 0.0174 | 0.4307 |
| 703 | Louzi Thani | 49A | North-Africa | 0.0022 | 0.0020 | 0.9792 | 0.0049 | 0.0117 |
| 704 | Najjar | 4B | North-Africa | 0.0191 | 0.0025 | 0.9492 | 0.0070 | 0.0222 |
| 705 | Ben Souileh | 53A | North-Africa | 0.0026 | 0.0041 | 0.9458 | 0.0048 | 0.0427 |
| 706 | Bedri Louzi | 57A | North-Africa | 0.0025 | 0.0025 | 0.6257 | 0.0296 | 0.3397 |
| 707 | Thani | 58B | North-Africa | 0.0022 | 0.0021 | 0.9791 | 0.0049 | 0.0117 |
| 708 | Theleth | 59A | North-Africa | 0.0020 | 0.0022 | 0.8893 | 0.0191 | 0.0874 |
| 709 | Amor El Euch | 5C | North-Africa | 0.0033 | 0.0032 | 0.9063 | 0.0034 | 0.0839 |
| 710 | Bou Khobza | 62A | North-Africa | 0.0025 | 0.0078 | 0.9634 | 0.0166 | 0.0097 |
| 711 | Mazouzi | 65B | North-Africa | 0.0032 | 0.0034 | 0.9277 | 0.0033 | 0.0625 |
| 712 | Messelmani | 6A | North-Africa | 0.0022 | 0.0147 | 0.9495 | 0.0162 | 0.0174 |
| 713 | Agrégé de Messelmani | 6B | North-Africa | 0.0028 | 0.0021 | 0.9705 | 0.0052 | 0.0194 |
| 714 | Zbidi | 7C | North-Africa | 0.0066 | 0.0033 | 0.8747 | 0.0980 | 0.0174 |
| 715 | Canino | A0795 | North-Africa | 0.0025 | 0.0020 | 0.0064 | 0.0120 | 0.9771 |
| 716 | kasserine n°2 Bergeron | A1592 | North-Africa | 0.0026 | 0.0021 | 0.0086 | 0.5006 | 0.4861 |
| 717 | kasserine n°1 polonais | A1594 | North-Africa | 0.0066 | 0.0028 | 0.0075 | 0.0036 | 0.9796 |
| 718 | Hatif Colomer | A1598 | North-Africa | 0.9564 | 0.0025 | 0.0112 | 0.0091 | 0.0208 |
| 719 | Marouch 4 | A2065 | North-Africa | 0.0020 | 0.0021 | 0.0035 | 0.4229 | 0.5694 |
| 720 | Marouch 14 | A2067 | North-Africa | 0.0024 | 0.0021 | 0.0076 | 0.0046 | 0.9833 |
| 721 | Marouch 16 | A2102 | North-Africa | 0.0024 | 0.0021 | 0.0081 | 0.0051 | 0.9823 |
| 722 | BAKOUR | A2137 | North-Africa | 0.0018 | 0.0022 | 0.9417 | 0.0403 | 0.0140 |
| 723 | Louzi Local | Al.01 | North-Africa | 0.0017 | 0.0016 | 0.0027 | 0.9911 | 0.0029 |
| 724 | Boulila Rouge | Al.02 | North-Africa | 0.0022 | 0.0019 | 0.0062 | 0.0077 | 0.9821 |
| 725 | Canino | Al.04 | North-Africa | 0.0114 | 0.0278 | 0.0308 | 0.0078 | 0.9223 |
| 726 | Louzi Rouge | Al.05 | North-Africa | 0.0066 | 0.0028 | 0.0075 | 0.0036 | 0.9796 |
| 727 | Louzi blanc | Al.06 | North-Africa | 0.0065 | 0.0028 | 0.0075 | 0.0036 | 0.9796 |
| 728 | Rosé de Corail | Al.07 | North-Africa | 0.0023 | 0.0150 | 0.0324 | 0.2391 | 0.7111 |
| 729 | Rosé de Ménaa | Al.08 | North-Africa | 0.0076 | 0.0046 | 0.7476 | 0.0127 | 0.2275 |
| 730 | Paviot Rouge | Al.09 | North-Africa | 0.0019 | 0.0022 | 0.0057 | 0.2236 | 0.7665 |
| 731 | Paviot blanc | Al.10 | North-Africa | 0.0018 | 0.0023 | 0.0040 | 0.2236 | 0.7683 |
| 732 | Boulida | Al.11 | North-Africa | 0.0061 | 0.0103 | 0.0066 | 0.0079 | 0.9690 |
| 733 | Rouge du Roussillon | Al.12 | North-Africa | 0.8277 | 0.0033 | 0.0499 | 0.0284 | 0.0908 |
| 734 | Pêcher | Al.13 | North-Africa | 0.0024 | 0.0020 | 0.0086 | 0.5145 | 0.4725 |
| 735 | Mechmech Hlou | Al.14 | North-Africa | 0.0444 | 0.0364 | 0.4015 | 0.0141 | 0.5036 |
| 736 | Gros MechMech | Al.15 | North-Africa | 0.0066 | 0.0027 | 0.0075 | 0.0036 | 0.9796 |
| 737 | Mechmech Laghdech | Al.16 | North-Africa | 0.0026 | 0.0040 | 0.4598 | 0.0289 | 0.5048 |
| 738 | Nail | Al.17 | North-Africa | 0.0027 | 0.0026 | 0.9812 | 0.0099 | 0.0037 |
| 739 | Abyad el Imlak | Al.18 | North-Africa | 0.0021 | 0.0022 | 0.9825 | 0.0061 | 0.0071 |
| 740 | Laouzi greffé | Al.19 | North-Africa | 0.0018 | 0.0022 | 0.0039 | 0.2234 | 0.7686 |
| 741 | Nda el Morjane | Al.20 | North-Africa | 0.0021 | 0.0021 | 0.9826 | 0.0060 | 0.0071 |
| 742 | Pêcher Rouge | Al.21 | North-Africa | 0.0030 | 0.0037 | 0.9789 | 0.0069 | 0.0075 |
| 743 | Arbi V1 | Al.22 | North-Africa | 0.0030 | 0.0081 | 0.9442 | 0.0041 | 0.0406 |
| 744 | Arbia Nadir | Al.23 | North-Africa | 0.0027 | 0.0023 | 0.9830 | 0.0076 | 0.0045 |
| 745 | Mahalat el Djoundi | Al.24 | North-Africa | 0.0026 | 0.0025 | 0.9743 | 0.0073 | 0.0133 |
| 746 | Louzia greffé | Al.25 | North-Africa | 0.0062 | 0.0029 | 0.0085 | 0.0030 | 0.9793 |
| 747 | Arbi Kd | Al.26 | North-Africa | 0.0029 | 0.0021 | 0.9696 | 0.0148 | 0.0106 |
| 748 | Pêcher Blanc | Al.27 | North-Africa | 0.0022 | 0.0019 | 0.0062 | 0.0076 | 0.9821 |
| 749 | Mouzemèche | Al.28 | North-Africa | 0.0020 | 0.0025 | 0.4235 | 0.0145 | 0.5575 |
| 750 | Kahf | Al.29 | North-Africa | 0.0020 | 0.0032 | 0.4196 | 0.1732 | 0.4019 |
| 751 | Messaad Greffé | Al.30 | North-Africa | 0.0024 | 0.0020 | 0.0071 | 0.5051 | 0.4834 |
| 752 | Louizi Rouge | Al.31 | North-Africa | 0.0022 | 0.0033 | 0.7828 | 0.2023 | 0.0095 |
| 753 | Arbi Nadir | Al.32 | North-Africa | 0.0021 | 0.0021 | 0.9720 | 0.0070 | 0.0168 |
| 754 | Saafi Arbi | Al.33 | North-Africa | 0.0023 | 0.0070 | 0.5991 | 0.0362 | 0.3555 |
| 755 | El Maghreb | Al.34 | North-Africa | 0.0247 | 0.0040 | 0.9503 | 0.0063 | 0.0147 |
| 756 | Hamrai | Al.35 | North-Africa | 0.0071 | 0.9429 | 0.0267 | 0.0089 | 0.0144 |
| 757 | Moutaakhir | Al.36 | North-Africa | 0.0021 | 0.0042 | 0.4289 | 0.0811 | 0.4837 |
| 758 | Hmarai tardif | Al.37 | North-Africa | 0.0018 | 0.0022 | 0.0039 | 0.2236 | 0.7684 |
| 759 | Pêcher blanc M | Al.38 | North-Africa | 0.0022 | 0.0019 | 0.0063 | 0.0075 | 0.9821 |
| 760 | El Bakria | Al.39 | North-Africa | 0.0023 | 0.0029 | 0.9182 | 0.0701 | 0.0065 |
| 761 | Douk el Kamel | Al.40 | North-Africa | 0.0021 | 0.0021 | 0.9835 | 0.0067 | 0.0056 |
| 762 | Bulida M | Al.41 | North-Africa | 0.0018 | 0.0022 | 0.0040 | 0.2237 | 0.7682 |
| 763 | Mnadir greffé M | Al.42 | North-Africa | 0.0022 | 0.0019 | 0.0062 | 0.0075 | 0.9822 |
| 764 | Hamrai Greffé | Al.43 | North-Africa | 0.0026 | 0.0091 | 0.9508 | 0.0103 | 0.0271 |
| 765 | Percher sur franc | Al.44 | North-Africa | 0.0022 | 0.0019 | 0.0062 | 0.0076 | 0.9820 |
| 766 | Chems el Massa | Al.45 | North-Africa | 0.0025 | 0.0024 | 0.9829 | 0.0062 | 0.0060 |
| 767 | Naila | Al.46 | North-Africa | 0.0020 | 0.0022 | 0.9728 | 0.0176 | 0.0054 |
| 768 | Saib Ennahdha | Al.47 | North-Africa | 0.0020 | 0.0023 | 0.9740 | 0.0133 | 0.0084 |
| 769 | Ikhtiyar ettayeb | Al.48 | North-Africa | 0.0025 | 0.0028 | 0.8680 | 0.0278 | 0.0990 |
| 770 | Kasserine K2 | K2 | North-Africa | 0.0024 | 0.0020 | 0.0086 | 0.5242 | 0.4627 |
| 771 | Goulmima AJG1 | L1V1 | North-Africa | 0.0027 | 0.0030 | 0.9693 | 0.0139 | 0.0112 |
| 772 | Kalaat Meggouna G1 | L1V10 | North-Africa | 0.0058 | 0.0027 | 0.8888 | 0.0065 | 0.0961 |
| 773 | Kalaat Meggouna G2 | L1V11 | North-Africa | 0.0016 | 0.0065 | 0.7737 | 0.0182 | 0.2000 |
| 774 | Jorf 7 | L1V3 | North-Africa | 0.0477 | 0.0699 | 0.8684 | 0.0058 | 0.0083 |
| 775 | Jorf 8 | L1V4 | North-Africa | 0.0020 | 0.0042 | 0.9804 | 0.0084 | 0.0049 |
| 776 | Goulmima AJG2 | L1V5 | North-Africa | 0.0021 | 0.0030 | 0.9838 | 0.0066 | 0.0045 |
| 777 | Boumalen J3 | L1V6 | North-Africa | 0.0233 | 0.0233 | 0.9260 | 0.0079 | 0.0194 |
| 778 | Agdez A7 | L1V7 | North-Africa | 0.0023 | 0.0024 | 0.0062 | 0.0047 | 0.9844 |
| 779 | Agdez A8 | L1V8 | North-Africa | 0.0023 | 0.0026 | 0.0066 | 0.0039 | 0.9846 |
| 780 | Skoura SKH2 | L1V9 | North-Africa | 0.0058 | 0.0026 | 0.9446 | 0.0074 | 0.0397 |
| 781 | Rich RK1 | L2V1 | North-Africa | 0.0041 | 0.0157 | 0.0129 | 0.0038 | 0.9635 |
| 782 | Rich 3 | L2V3 | North-Africa | 0.0019 | 0.0020 | 0.0059 | 0.0035 | 0.9868 |
| 783 | Rich 3 | L2V4 | North-Africa | 0.0022 | 0.0025 | 0.9818 | 0.0041 | 0.0093 |
| 784 | Jorf 6 | L2V5 | North-Africa | 0.0023 | 0.0020 | 0.9827 | 0.0054 | 0.0077 |
| 785 | Kalaat Meggouna G6 | L2V6 | North-Africa | 0.0106 | 0.0407 | 0.2508 | 0.0133 | 0.6845 |
| 786 | Boumalen A3 | L2V8 | North-Africa | 0.0057 | 0.0126 | 0.9596 | 0.0068 | 0.0152 |
| 787 | Rich 4 | L3V1 | North-Africa | 0.0023 | 0.0064 | 0.9823 | 0.0035 | 0.0055 |
| 788 | Agdez C1 | L3V11 | North-Africa | 0.1920 | 0.0025 | 0.7932 | 0.0039 | 0.0084 |
| 789 | Goulmima RG1 | L3V2 | North-Africa | 0.0025 | 0.0184 | 0.9661 | 0.0071 | 0.0058 |
| 790 | Rtil 1 | L3V3 | North-Africa | 0.0027 | 0.0032 | 0.4411 | 0.0028 | 0.5501 |
| 791 | Rtil 2 | L3V4 | North-Africa | 0.0017 | 0.0016 | 0.0028 | 0.9907 | 0.0031 |
| 792 | Can 1 | L3V5 | North-Africa | 0.0022 | 0.0019 | 0.0062 | 0.0077 | 0.9821 |
| 793 | Kalaat Meggouna G5 | L3V7 | North-Africa | 0.0411 | 0.0087 | 0.2273 | 0.0467 | 0.6762 |
| 794 | Skoura SKH3 | L3V8 | North-Africa | 0.0031 | 0.0020 | 0.9868 | 0.0025 | 0.0057 |
| 795 | Boumalen A4 | L3V9 | North-Africa | 0.0036 | 0.0026 | 0.9811 | 0.0054 | 0.0074 |
| 796 | Rtil 4 | L4V1 | North-Africa | 0.0023 | 0.0808 | 0.1527 | 0.0093 | 0.7549 |
| 797 | Agdez A6 | L4V10 | North-Africa | 0.0030 | 0.0023 | 0.9711 | 0.0113 | 0.0123 |
| 798 | Rtil 5 | L4V2 | North-Africa | 0.0023 | 0.0035 | 0.9871 | 0.0031 | 0.0041 |
| 799 | Goulmima GR1 | L4V3 | North-Africa | 0.0022 | 0.0024 | 0.9877 | 0.0033 | 0.0045 |
| 800 | Goulmima GM1 | L4V4 | North-Africa | 0.0044 | 0.0034 | 0.9236 | 0.0122 | 0.0564 |
| 801 | Geli | L4V5 | North-Africa | 0.0097 | 0.0412 | 0.0201 | 0.0039 | 0.9251 |
| 802 | Skoura SKT1 | L4V6 | North-Africa | 0.0056 | 0.0060 | 0.9772 | 0.0031 | 0.0080 |
| 803 | Skoura SKH1 | L4V7 | North-Africa | 0.0034 | 0.0026 | 0.9817 | 0.0053 | 0.0071 |
| 804 | Outat Elhaj 7 | L4V8 | North-Africa | 0.0017 | 0.0016 | 0.0027 | 0.9911 | 0.0029 |
| 805 | Kalaat Meggouna G7 | L4V9 | North-Africa | 0.0023 | 0.0043 | 0.2437 | 0.1054 | 0.6443 |
| 806 | Goulmima GAY1 | L5V1 | North-Africa | 0.0194 | 0.0160 | 0.9493 | 0.0093 | 0.0060 |
| 807 | Outat Elhaj 6 | L5V10 | North-Africa | 0.0023 | 0.0112 | 0.9524 | 0.0220 | 0.0121 |
| 808 | Boumalen A2 | L5V11 | North-Africa | 0.0032 | 0.0208 | 0.9686 | 0.0031 | 0.0043 |
| 809 | Goulmima GAY2 | L5V2 | North-Africa | 0.0047 | 0.0021 | 0.9712 | 0.0169 | 0.0052 |
| 810 | Goulmima G3 | L5V3 | North-Africa | 0.0219 | 0.0049 | 0.9597 | 0.0056 | 0.0079 |
| 811 | Missour V4 | L5V4 | North-Africa | 0.0021 | 0.0023 | 0.5228 | 0.0069 | 0.4658 |
| 812 | Rtil 3 | L5V5 | North-Africa | 0.0025 | 0.0034 | 0.9741 | 0.0092 | 0.0107 |
| 813 | Missour V2 | L5V6 | North-Africa | 0.0290 | 0.0026 | 0.9400 | 0.0154 | 0.0130 |
| 814 | Outat Elhaj 2 | L5V8 | North-Africa | 0.0021 | 0.0021 | 0.0059 | 0.0147 | 0.9751 |
| 815 | Outat Elhaj 3 | L5V9 | North-Africa | 0.0022 | 0.0019 | 0.0063 | 0.0076 | 0.9819 |
| 816 | Marrouch 3 | L6V1 | North-Africa | 0.0020 | 0.0019 | 0.0143 | 0.0351 | 0.9466 |
| 817 | Boumalen A1 | L6V10 | North-Africa | 0.0024 | 0.0023 | 0.9786 | 0.0071 | 0.0097 |
| 818 | Guersif 2 | L6V11 | North-Africa | 0.0154 | 0.0328 | 0.9431 | 0.0042 | 0.0045 |
| 819 | Marrouch 16 | L6V3 | North-Africa | 0.0025 | 0.0021 | 0.0076 | 0.0046 | 0.9833 |
| 820 | Marrouch 4 | L6V4 | North-Africa | 0.0022 | 0.0019 | 0.0063 | 0.0076 | 0.9820 |
| 821 | Khorb | L6V5 | North-Africa | 0.0024 | 0.0023 | 0.9851 | 0.0043 | 0.0059 |
| 822 | Maoui | L6V6 | North-Africa | 0.0023 | 0.0025 | 0.0065 | 0.0040 | 0.9847 |
| 823 | Outat Elhaj1 | L6V8 | North-Africa | 0.0030 | 0.0041 | 0.4657 | 0.0042 | 0.5229 |
| 824 | Kalaat Meggouna G8 | L6V9 | North-Africa | 0.0031 | 0.0023 | 0.8831 | 0.0234 | 0.0881 |
| 825 | Gmat | L7V1 | North-Africa | 0.0023 | 0.0021 | 0.9774 | 0.0048 | 0.0134 |
| 826 | Skoura SKT5 | L7V11 | North-Africa | 0.0023 | 0.0033 | 0.9809 | 0.0045 | 0.0090 |
| 827 | Mans | L7V2 | North-Africa | 0.0024 | 0.0020 | 0.0086 | 0.5153 | 0.4718 |
| 828 | Del Patricia | L7V3 | North-Africa | 0.0023 | 0.0024 | 0.0061 | 0.0046 | 0.9846 |
| 829 | Missour V17 | L7V4 | North-Africa | 0.0022 | 0.0026 | 0.9791 | 0.0049 | 0.0113 |
| 830 | Boumalen KH1 | L7V6 | North-Africa | 0.0024 | 0.0033 | 0.9303 | 0.0064 | 0.0576 |
| 831 | Outat Elhaj 8 | L7V7 | North-Africa | 0.0020 | 0.0019 | 0.0060 | 0.0050 | 0.9851 |
| 832 | Agdez A5 | L7V8 | North-Africa | 0.0030 | 0.0020 | 0.9849 | 0.0032 | 0.0069 |
| 833 | Missour V12 | L8V1 | North-Africa | 0.0025 | 0.0056 | 0.9763 | 0.0043 | 0.0114 |
| 834 | Skoura SKH4 | L8V10 | North-Africa | 0.0043 | 0.0078 | 0.9665 | 0.0072 | 0.0141 |
| 835 | SK1 | L8V11 | North-Africa | 0.0023 | 0.0024 | 0.0060 | 0.0047 | 0.9846 |
| 836 | Agdez A4 | L8V12 | North-Africa | 0.0103 | 0.0060 | 0.8132 | 0.0028 | 0.1677 |
| 837 | Agdez IG1 | L8V15 | North-Africa | 0.0029 | 0.0030 | 0.0560 | 0.0057 | 0.9322 |
| 838 | Rich RT1 | L8V3 | North-Africa | 0.0030 | 0.0078 | 0.0051 | 0.0038 | 0.9803 |
| 839 | Marrouch 1 | L8V4 | North-Africa | 0.0040 | 0.0111 | 0.9634 | 0.0031 | 0.0183 |
| 840 | Missour V3 | L8V5 | North-Africa | 0.0757 | 0.0425 | 0.1217 | 0.0290 | 0.7311 |
| 841 | Missour V15 | L8V7 | North-Africa | 0.0025 | 0.0048 | 0.3262 | 0.0034 | 0.6631 |
| 842 | Kalaat Meggouna G9 | L8V8 | North-Africa | 0.0022 | 0.0021 | 0.9806 | 0.0094 | 0.0056 |
| 843 | Boumalen J2 | L8V9 | North-Africa | 0.0034 | 0.0021 | 0.9782 | 0.0048 | 0.0115 |
| 844 | Missour V22 | L9V1 | North-Africa | 0.0025 | 0.0029 | 0.9778 | 0.0075 | 0.0094 |
| 845 | Kalaat Meggouna G3 | L9V3 | North-Africa | 0.0035 | 0.0023 | 0.9794 | 0.0037 | 0.0111 |
| 846 | Perfection | 12PB407 | America | 0.5266 | 0.0023 | 0.0030 | 0.0026 | 0.4655 |
| 847 | Morden 604 | 12PB408 | America | 0.0967 | 0.8909 | 0.0035 | 0.0034 | 0.0056 |
| 848 | Sun Glo | 12PB409 | America | 0.0034 | 0.9683 | 0.0177 | 0.0033 | 0.0073 |
| 849 | Reliable | 12PB410 | America | 0.0274 | 0.0891 | 0.1420 | 0.1882 | 0.5533 |
| 850 | A157 | 12PB598 | America | 0.0065 | 0.0026 | 0.0280 | 0.0092 | 0.9537 |
| 851 | 1384-2 | 12PB691 | America | 0.0044 | 0.8957 | 0.0644 | 0.0038 | 0.0317 |
| 852 | 1393-5 | 12PB692 | America | 0.0025 | 0.9822 | 0.0059 | 0.0035 | 0.0060 |
| 853 | 1394-1 | 12PB693 | America | 0.0051 | 0.9143 | 0.0632 | 0.0093 | 0.0081 |
| 854 | 1395-1 | 12PB694 | America | 0.0119 | 0.7298 | 0.2198 | 0.0161 | 0.0224 |
| 855 | 1416-4 | 12PB695 | America | 0.1070 | 0.7259 | 0.0169 | 0.0216 | 0.1287 |
| 856 | Seo | A1145 | America | 0.0083 | 0.9531 | 0.0229 | 0.0066 | 0.0092 |
| 857 | Stark Early Orange faux | A1145 | America | 0.0029 | 0.1859 | 0.0142 | 0.4780 | 0.3189 |
| 858 | SR7 | A1170 | America | 0.1593 | 0.0099 | 0.0384 | 0.0058 | 0.7865 |
| 859 | Good Sweet Kernel EM1 | A1608 | America | 0.0028 | 0.0043 | 0.4753 | 0.5081 | 0.0094 |
| 860 | Sophia | A1609 | America | 0.0029 | 0.0043 | 0.4758 | 0.5077 | 0.0093 |
| 861 | Early Orange | A1717 | America | 0.0075 | 0.9357 | 0.0278 | 0.0108 | 0.0181 |
| 862 | King | A1752 | America | 0.0130 | 0.5931 | 0.1234 | 0.0449 | 0.2256 |
| 863 | Stella | A2382 | America | 0.0022 | 0.3229 | 0.6449 | 0.0172 | 0.0128 |
| 864 | Skaha | A2633 | America | 0.3142 | 0.0109 | 0.0665 | 0.1320 | 0.4764 |
| 865 | Perfection | A2634 | America | 0.6183 | 0.0023 | 0.0031 | 0.0025 | 0.3739 |
| 866 | Riland | A2637 | America | 0.0112 | 0.5831 | 0.1002 | 0.1459 | 0.1596 |
| 867 | Royalty | A2848 | America | 0.2067 | 0.4704 | 0.0058 | 0.0133 | 0.3038 |
| 868 | Dwarf | A2853 | America | 0.4214 | 0.0032 | 0.0042 | 0.0028 | 0.5684 |
| 869 | Morocot | A2923 | America | 0.2414 | 0.3137 | 0.0043 | 0.0061 | 0.4345 |
| 870 | Royal | A44 | America | 0.0019 | 0.0022 | 0.0064 | 0.0068 | 0.9827 |
| 871 | Suizo | A519 | America | 0.0020 | 0.0027 | 0.1113 | 0.0076 | 0.8763 |
| 872 | Henderson | A634 | America | 0.0146 | 0.7300 | 0.1294 | 0.0117 | 0.1143 |
| 873 | Pseudo Royal | A862 | America | 0.0027 | 0.2846 | 0.6968 | 0.0044 | 0.0115 |
| 874 | Stark Early Orange | Tk62 | America | 0.0070 | 0.9423 | 0.0275 | 0.0089 | 0.0143 |
| 875 | Brigantina | U0937 | America | 0.0021 | 0.9892 | 0.0023 | 0.0044 | 0.0020 |
| 876 | Hemskirke | U0946 | America | 0.0027 | 0.0024 | 0.0544 | 0.0028 | 0.9377 |
| 877 | Large Early Montgemet | U1504 | America | 0.0017 | 0.0017 | 0.0025 | 0.9917 | 0.0025 |
| 878 | Maxson | U1505 | America | 0.0350 | 0.0034 | 0.0047 | 0.0322 | 0.9247 |
| 879 | Santa Clara Sweet | U2375 | America | 0.2154 | 0.1589 | 0.0084 | 0.0071 | 0.6101 |
| 880 | Canada White | U2376 | America | 0.0077 | 0.3461 | 0.6357 | 0.0064 | 0.0040 |
| 881 | Shalah | U2377 | Irano-Caucasian | 0.0034 | 0.0376 | 0.6295 | 0.0406 | 0.2889 |
| 882 | Casa Soberanes | U2378 | America | 0.0020 | 0.0028 | 0.0061 | 0.0104 | 0.9787 |
| 883 | Stephen's Favorite | U2379 | America | 0.0024 | 0.0026 | 0.0040 | 0.4306 | 0.5604 |
| 884 | Chenton | U2380 | America | 0.0102 | 0.8251 | 0.0325 | 0.0295 | 0.1026 |
| 885 | Nugget | U2381 | America | 0.0035 | 0.5126 | 0.0107 | 0.0123 | 0.4610 |
| 886 | Derby Royal | U2382 | America | 0.0019 | 0.0020 | 0.0037 | 0.0032 | 0.9891 |
| 887 | Basque | U2383 | America | 0.0033 | 0.0039 | 0.0430 | 0.4946 | 0.4553 |
| 888 | San Fernando Supreme | U2384 | America | 0.5290 | 0.0026 | 0.0031 | 0.0026 | 0.4628 |
| 889 | Supkany | U2385 | Central Asia | 0.0084 | 0.7222 | 0.2234 | 0.0299 | 0.0160 |
| 890 | CP 15-1 | U2424 | America | 0.5099 | 0.0120 | 0.4348 | 0.0143 | 0.0290 |
