# Supplementary material for: Genetic Structure of a Worldwide Germplasm Collection of Prunus armeniaca L. Reveals Three Major Diffusion Routes for Varieties Coming From the Species’ Center of Origin
Source: Front Plant Sci. 2020 May 25;11:638. doi: 10.3389/fpls.2020.00638 (PMC7261834; doi:10.3389/fpls.2020.00638)
Supplement: ADDITIONAL FILE S1 — List of the 890 apricot accessions considered in the present study. Accession code, name, site of collection, geographical group of origin, cluster assignment inferred by the STRUCTURE analysis are reported. Accessions with asterisk are core collections members. [file Data_Sheet_1.zip › Additional File 7.doc]

Additional file 7. Number and proportion (in parentheses) of accessions from different geographic groups assigned to the five clusters identified by STRUCURE analysis.

| Group | Total number of accessions | Number of assigned accessions^*^ | Cluster | | | | |
| --- | --- | --- | --- | --- | --- | --- | --- |
|  |  |  | cluster 1 | cluster 2 | cluster 3 | cluster 4 | cluster 5 |
| Group 1 : Eastern Asia | 67 | 58 (86.56) | 23 (39.65) | 33 (56.89) | 0 | 2 (3.44) | 0 |
| Group 2 : Central Asia | 142 | 100 (70.42) | 1 (0.01) | 94 (94) | 2 (0.02) | 1 (0.01) | 2 (0.02) |
| Group 3 : Irano-Caucasian | 86 | 43 (50) | 0 | 5 (11.62) | 31 (72.09) | 1 (2.32) | 6 (13.95) |
| Group 4 : Continental Europe | 86 | 59 (68.60) | 0 | 4 (6.77) | 0 | 48 (81.35) | 7 (11.86) |
| Group 5 : Mediterranean Europe | 250 | 146 (58.40) | 18 (12.32) | 2 (1.36) | 11 (7.53) | 8 (5.47) | 107 (73.28) |
| Group 6 : North Africa | 214 | 166 (77.57) | 2 (0.01) | 1 (0.60) | 119 (71.68) | 3 (1.80) | 41 (24.69) |
| Group 7 : America | 45 | 18 (40) | 0 | 10 (55.55) | 0 | 1 (5.55) | 7 (38.88) |
| Total | 890 | 590 (66.29) | 44 | 149 | 163 | 64 | 170 |

^*^Accessions with proportion ≥0.8 of membership assignment in a cluster
