# Supplementary material for: Genetic Structure of a Worldwide Germplasm Collection of Prunus armeniaca L. Reveals Three Major Diffusion Routes for Varieties Coming From the Species’ Center of Origin
Source: Front Plant Sci. 2020 May 25;11:638. doi: 10.3389/fpls.2020.00638 (PMC7261834; doi:10.3389/fpls.2020.00638)
Supplement: ADDITIONAL FILE S1 — List of the 890 apricot accessions considered in the present study. Accession code, name, site of collection, geographical group of origin, cluster assignment inferred by the STRUCTURE analysis are reported. Accessions with asterisk are core collections members. [file Data_Sheet_1.zip › Additional File 8.doc]

Additional file 8. Parameters and results for bottleneck analyses used to detect significant reductions in effective population size in apricot clusters.

|  |  | Cluster 1 | Cluster 2 | Cluster 3 | Cluster 4 | Cluster 5 |
| --- | --- | --- | --- | --- | --- | --- |
| Wilcoxon signed-rank test^*^ | IAM | 0.0258^**^ | 0.0023^**^ | 0.2371 | 0.1500 | 0.0954 |
|  | TPM | 1.0000 | 1.0000 | 1.0000 | 0.9369 | 1.0000 |
|  | SMM | 1.0000 | 1.0000 | 1.0000 | 0.9599 | 1.0000 |

IAM: Infinite allele model, TPM: Two-phase mutation model, SMM: Stepwise mutation model.

^*^*P*-value for one tail test for heterozygosity excess. ^**^ *P*-value < 0.05
